# Rapid Evolution in a Coral Population Following a Mass Mortality Event

**DOI:** 10.1111/eva.70198

**Published:** 2026-02-01

**Authors:** James E. Fifer, Kelly E. Speare, Sarah E. Leinbach, Stephanie F. Hendricks, Sarah W. Davies, Noah H. Rose, Deron E. Burkepile, Thomas C. Adam, Gretchen E. Hofmann, Marie E. Strader

**Affiliations:** ^1^ Department of Ecology, Behavior, and Evolution University of California San Diego San Diego California USA; ^2^ Department of Biology Boston University Boston Massachusetts USA; ^3^ Department of Biology Texas A&M University College Station Texas USA; ^4^ School of Geographical Sciences and Urban Planning Arizona State University Tempe Arizona USA; ^5^ Department of Ecology, Evolution, and Marine Biology University of California, Santa Barbara Santa Barbara California USA; ^6^ Department of Biological Sciences Auburn University Auburn Alabama USA; ^7^ Department of Marine and Environmental Sciences Nova Southeastern University Fort Lauderdale Florida USA; ^8^ Marine Science Institute, University of California, Santa Barbara Santa Barbara California USA

## Abstract

Globally, corals face an increased frequency of mass mortality events (MMEs) as populations experience repeated marine heatwaves which disrupt their obligate algal symbiosis. Despite greater occurrences of MMEs, the relative roles of the environment, host, and symbiont genetic variation in survival, subsequent recovery, and carry‐over effects to the next generation remain unresolved. High‐resolution temporal and spatial whole genome sequencing of corals before, after, and several years following an MME reveal that host genetics have an impact on bleaching and mortality and that selected alleles important for adaptation persist through the next generation, demonstrating rapid evolution in this coral population. Bleaching resistance and survival following the bleaching event were highly polygenic, and allele frequency shifts show reef habitat specificity, emphasizing the spatial complexity of environmental selection and how it shapes population recovery following an MME. This study reveals how MMEs reshape the genomic landscape and the spatial and temporal distribution of genomic diversity within coral populations facing severe threats from global change.

## 1 Introduction

As a consequence of anthropogenic global change, mass mortality events (MMEs) are increasing in both frequency and magnitude across diverse marine ecosystems (Fey et al. 2015). Rapid decreases in population size are theorized to lower genetic diversity (Nei et al. 1975) and thus reduce the variation upon which selection acts, constraining the adaptability of survivors to future stressors (Du et al. 2016; Radwan et al. 2010). Despite this, MME‐driven reductions in genetic diversity have limited empirical support in marine systems (but see Gurgel et al. 2020). It is unclear if this is due to temporal limitations of many MME genetic diversity studies (e.g., sampling only one generation following an MME) or rooted in population dynamics that protect against a loss of genetic diversity (e.g., large population sizes; Nei et al. 1975). MMEs can also lead to a genomic signature of selection for advantageous alleles (e.g., Auteri and Knowles 2020; Campbell‐Staton et al. 2017; Gignoux‐Wolfsohn et al. 2021; Holland et al. 2022; Schiebelhut et al. 2018). Observed signatures of selection suggest population persistence by survivors may allow future generations to be better equipped against stressors similar to those that triggered the MME. As MMEs shape the evolutionary direction of populations and species, their increased prevalence presents a critical concern for the long‐term viability and resilience of marine ecosystems.

Coral reefs, which are responsible for hosting over a quarter of the ocean's total biodiversity (Knowlton et al. 2010), have faced numerous MMEs globally in recent decades due to sea surface temperatures exceeding their physiological limits (Eakin et al. 2019). MMEs in reef‐building corals typically result from marine heatwaves (MHWs) that cause coral bleaching, the breakdown of the symbiosis between the coral and its endosymbiotic microalgal partner (family Symbiodiniaceae) (Douglas 2003). Despite corals' vulnerability to increased temperatures, variation in response to heat stress is observed within and among coral species (Dixon et al. 2015; Loya et al. 2001). While this variation can be at least partially attributed to the genera or species of symbiont hosted (e.g., Baker et al. 2004; Berkelmans and Van Oppen 2006; Jones et al. 2008; Kemp et al. 2023) and microenvironment differences (Fabricius 2006), there is evidence for a heritable host basis for heat stress resilience (Quigley and van Oppen 2022; and reviewed in Bairos‐Novak et al. 2021; Howells et al. 2022), suggesting there is variation in heat stress tolerance for selection to act upon during MMEs.

How fast a species can adapt to novel environments from standing genetic variation is an outstanding question in evolutionary ecology and critically time‐sensitive, as organisms face rapidly changing environments globally. It has been theorized that long‐term reef resilience relies on natural selection favoring heat‐tolerant alleles that exist in the metapopulation prior to warming (Matz et al. 2020). While advances in DNA sequencing technology have been leveraged to identify genetic loci that evolved in response to natural selection in corals (Cooke et al. 2020; Rose et al. 2021; Smith et al. 2022; Zhang et al. 2022), these studies are inherently limited because they investigate selection indirectly, finding its signature in nearby neutral variation. Temporal sampling across an MME allows for a direct examination of loci under selection, but to date coral genomic research has focused on a single timepoint of an MME (e.g., bleaching timepoint; Fuller et al. 2020) or used too few molecular markers and/or individuals (Thomas et al. 2024) to capture evolutionary changes.

In 2019, a prolonged MHW occurred around the island of Mo'orea, French Polynesia, and the major reef‐building genus of corals, *Acropora*, experienced 50%–80% mortality as a consequence of severe, prolonged bleaching (Speare et al. 2022). We performed whole genome sequencing on *Acropora hyacinthus* colonies from multiple sites around Mo'orea: (1) during the peak of the bleaching event in May 2019 before mortality, (2) after bleaching and thermal stress had subsided in October 2019, and (3) in November 2021, 2 years after the MME, when new recruits naïve to the 2019 bleaching event were collected. High‐resolution sampling of the population before and after the mortality event and in the subsequent generation allowed us to assess temporal genomic signatures of MHW resistance and to answer the following questions: (1) Can host genetics explain survival from a bleaching event and what is the genetic architecture of the trait? (2) Are adaptive alleles maintained in the following generation? (3) Does adaptive genetic variation differ between discrete reef habitats? (4) Do symbionts exert a direct, independent effect on bleaching susceptibility, or are observed patterns primarily a consequence of host‐driven structuring of symbiont assemblages?

## 2 Main

**FIGURE 1:**
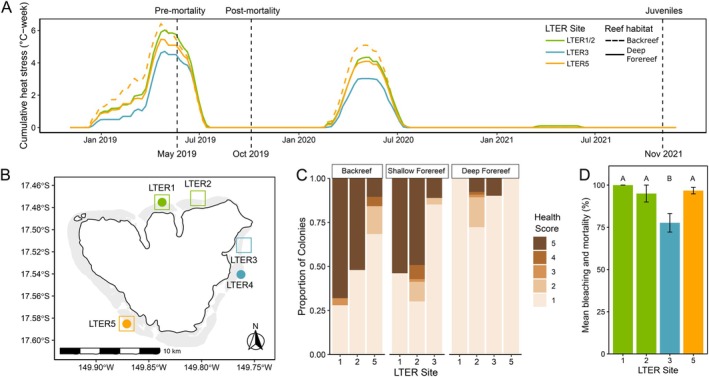
Pervasive bleaching and mortality following an extreme thermal anomaly. (A) Cumulative heat stress across space and time. Vertical lines denote sampling points for sequencing. (B) Mo'orea, French Polynesia. Circles represent locations where temperature data were collected; squares represent locations where samples were collected for sequencing. (C) *Acropora hyacinthus* bleaching severity observed across reef habitats and sites during May 2019 surveys (i.e., pre‐mortality timepoint). A score of 1 indicates complete bleaching and 5 indicates no pigmentation loss (example photos in Figure S12; survey information in Table S1). (D) *Acropora hyacinthus* combined bleaching and mortality data from surveys in July 2019 at deep forereef locations (survey information in Table S1). Letters indicate significance per pairwise Wilcoxon rank sum test.

*Acropora hyacinthus* colonies were sampled for whole genome sequencing (WGS) around the island of Mo'orea as follows: (1) adults during the May 2019 bleaching event (hereafter, pre‐mortality), (2) adults after bleaching had subsided in October 2019 (hereafter, post‐mortality), and (3) juveniles (colonies < 8 cm long diameter) in November 2021 (hereafter, juveniles). For each timepoint, colonies were sampled randomly from four locations around the island that were directly adjacent to Mo'orea Coral Reef Long‐Term Ecological Research (MCR LTER) locations: LTER 1 and 2 on the north shore, LTER 3 on the east shore, and LTER 5 on the west shore (Figure 1B). At each MCR LTER location, colonies were sampled from at least one of three reef habitats (backreef, shallow forereef, and deep forereef), corresponding to three different depths (1–3 m, 3–5 m, 10–14 m, respectively). While adults from May 2019 and October 2019 included samples at all three reef habitats, juveniles from November 2021 were only sampled at the deep forereef due to weather constraints; thus for any comparisons between adults and juveniles, data were subset to look only at the deep forereef (Table S1). Hereafter, each combination of a reef habitat and an associated LTER location is referred to as a “site.” For WGS, individuals were selected from four LTER locations, three reef habitats and three timepoints. Not all LTER locations or reef habitats were sampled at all timepoints. This resulted in 19 unique sampling groups (LTER × habitat × timepoint), with 12–26 individuals each (after filtering for clones) for a total of 349 individuals (Table S1).

### 2.1 2019 Heatwave Triggers Mass Bleaching and Mortality

In 2019, Mo'orea experienced a prolonged marine heatwave (MHW), with sites experiencing 4–6 weeks of cumulative heat stress exceeding 29°C (Figure 1A), a noted bleaching threshold for most corals in Mo'orea (Pratchett et al. 2013). This was the largest thermal stress event recorded in Mo'orea in the last 16 years (Figure S1). *Acropora hyacinthus* experienced bleaching island‐wide, with the deep forereef particularly impacted (Figure 1C; Figure S12). Within the deep forereef, the northern (LTERs 1 and 2) and western (LTER 5) regions experienced the largest cumulative heat stress (Figure 1A) and subsequently displayed higher bleaching in May 2019 (Figure 1C) and higher bleaching and mortality in July 2019 (*p* < 0.01; Figure 1D) compared to the eastern side of the island (LTER 3). As a result of this heat wave, *Acropora* spp. exhibited the highest decrease in percent cover since the 2006–2009 outbreaks of the corallivorous seastar *Acanthaster planci* (Kayal et al. 2012; Figure S2). In 2020, temperature data revealed a second, smaller MHW, where sites experienced 3–5 weeks of cumulative heat stress exceeding 29°C (Figure 1A). The impact of this MHW on bleaching is unknown as COVID restrictions prohibited field observations of bleaching prevalence; however, *Acropora* spp. cover did not show a decrease following the 2020 MHW (Figure S2).

### 2.2 Low Genetic Structure in *A. hyacinthus* Mo'orea Populations

**FIGURE 2:**
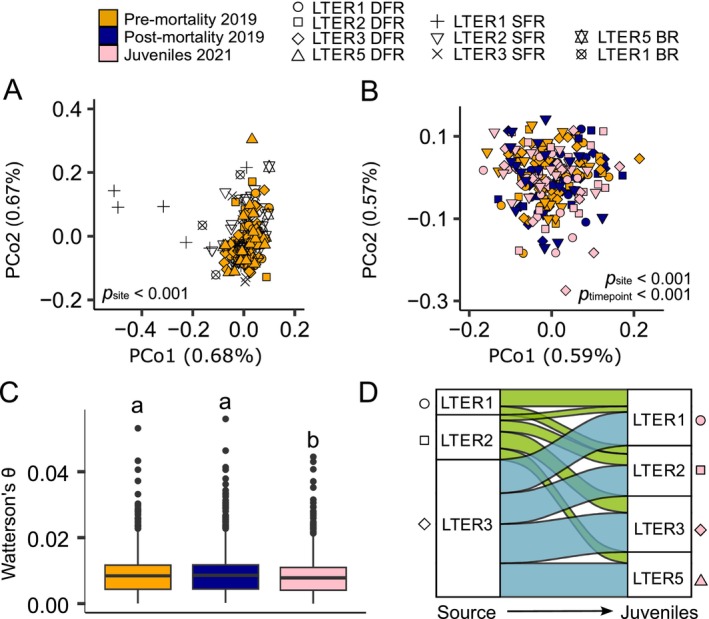
Stability of genetic diversity across adult corals and shifts in recruitment dynamics in a panmictic Mo'orean population. (A) Principal coordinates analysis (PCoA) plot using a 1—correlation transformation on the genetic covariance matrix of pre‐mortality (*N* = 139) samples shows weak population structure. Sites that were sampled across all three timepoints [i.e., those shown in panel (B) are color filled, sites that were sampled pre‐mortality but missing from post‐mortality or juvenile timepoints are open symbols]. (B) PCoA plot using samples from the sites that were sampled at all three timepoints highlighting the genome‐wide similarity of samples before (*N* = 86), after the bleaching event (*N* = 71), and juveniles 2 years later (*N* = 76), except the few juveniles that cluster outside the major grouping. (C) Nucleotide diversity (Watterson's *θ*) between timepoints for all overlapping sites; letters denote significant differences via Dunn's test (*p* < 0.001). (D) Discriminant analysis of principal components (DAPC) assignment between predicted source site (left column) and actual site of the juvenile (right column; *N* = 76) based on similarity to adult samples (all nine sites; *N* = 244), showing the large recruitment from LTER 3 deep forereef and lack of recruitment from backreef and shallow forereef sites (sites with no predicted recruitment are absent from left column). Colors correspond to the different coasts of Mo'orea (green: North; blue: East).

Prior to the heat‐induced mortality event in 2019, backreef and forereef sites around the island of Mo'orea showed evidence of low genetic structure; for example, *F*_ST_ between sites ranged from 0.013 to 0.02 (Figure 2A; Figure S3). Slightly higher differentiation between sites was observed when any site was compared with a backreef site (including backreef–backreef comparisons) (*F*_ST_ between sites ranging from 0.016 to 0.021; Figure S3), compared to deep forereef–deep forereef comparisons (*F*_ST_ between sites ranging from 0.012 to 0.016; Figure S3). Sites were significantly differentiated pre‐mortality event (*p*_permanova_ < 0.001), but the variation explained by site was low (*R* ^2^ = 0.048) (Figure 2B), and there was no observable signature of isolation by distance between pre‐mortality sites (Mantel test *p* = 0.47; Figure S4). Lack of strong population structure was also observed in admixture analyses showing an optimal *K* of 1 and no discernable structuring according to sites at higher *K*s (Figure S5). These results mirror *A. hyacinthus* 2b‐RAD‐seq data for populations in Mo'orea and Tahiti collected 6 years prior in 2013, where pairwise *F*_ST_ ranged from 0.011 to 0.018 (Kriefall et al. 2022). All Mo'orea samples clustered with the American Samoa *A. hyacinthus* lineage HE (now referred to as *A. turbinata* in Rassmussen et al. 2025), formerly “HE” from Rose et al. (2021) (Figure S6), which was the most thermally tolerant among the four cryptic *A. hyacinthus* lineages from Rose et al. (2021).

### 2.3 Genetic Diversity and Differentiation Show Stability Through Bleaching Event

Genetic diversity, measured as nucleotide diversity (Watterson's *θ*; Watterson 1975), showed no difference post‐mortality (October 2019) compared to pre‐mortality (May 2019) (Figure 2C), which was also true when comparing genetic diversity at the site level (Figure S7). We similarly find no differences in π, measured as pairwise *θ* (Figure S8), or individual heterozygosity (Figure S9) between pre‐ and post‐mortality timepoints. However, at the site level, LTER 2 deep forereef exhibited decreased heterozygosity and LTER 2 shallow forereef exhibited increased heterozygosity post‐mortality (Figure S10). Finally, pairwise genetic differentiation (*F*_ST_) between deep forereef sites showed stability across the mortality event with no deviation in site differentiation post‐mortality compared to pre‐mortality (Figure S11). Empirical evidence of loss of genetic diversity directly following MMEs in marine systems is limited (Gurgel et al. 2020), with many instances of no observable decrease in genetic diversity, such as in kelp (Coleman et al. 2020; Klingbeil et al. 2022), eels (Pujolar et al. 2011), sea stars (Schiebelhut et al. 2018), mangroves (Arnaud‐Haond et al. 2009), gorgonians (Pilczynska et al. 2016), and dogwhelk (Colson and Hughes 2004). The stability of genetic diversity following MMEs has occasionally been attributed to additional recruitment between timepoints (e.g., Klingbeil et al. 2022), which is not the case in our system, as 3 months is not enough time for *A. hyacinthus* to recruit and develop into adults. Alternatively, populations with large effective population sizes (*N*_e_) are not expected to exhibit decreases in genetic diversity following population bottlenecks (Frankham 1995; Franklin 1980; Nei et al. 1975). Contemporary estimates of *N*_e_ for these populations are consistent with this theory, with several populations having an estimate of > 4,500,000 *N*_e_ (Figure S12).

### 2.4 Mortality Event Disrupts Genetic Diversity and Differentiation in the Following Generation

In contrast to the observed lack of a change in genetic diversity pre‐ and post‐mortality, genetic diversity and individual heterozygosity island‐wide were lower in juveniles collected 2 years after the MME compared to adult populations from both pre‐ and post‐mortality timepoints (Figure 2C; Figure S9). To further explore differences in genetic variation between pre‐mortality corals and recruiting juveniles as well as potential source populations for juveniles, we ran a discriminant analysis of principal components (DAPC). DAPCs were performed using adult colonies from 2019 (both pre‐ and post‐mortality, all sites) only as training data. DAPCs were conditioned on site (irrespective of pre‐ or post‐mortality timepoint) and the resulting model predicted juvenile site assignment. Putative sources for juveniles collected post‐mortality event in 2021 were not evenly split between sites (*χ* ^2^ = 48.185, *p* < 0.0001), with LTER 3 deep forereef serving as the dominant source (Figure 2D). Furthermore, only deep forereef sites (LTERs 1, 2, and 3 deep forereef) were predicted sources of deep forereef juveniles, with a low likelihood of being sourced from either backreef, shallow forereef, or LTER 5 deep forereef locations (Figure 2D). Given visualizations of population clustering using principal correspondence analyses (PCoA) and DAPC, several juveniles fall outside the central genetic cluster (Figure 2B). These individuals likely represent immigrants from other islands or reefs not sampled here.

*Acropora hyacinthus* has shown a propensity to exhibit “sweepstakes reproductive success” (SRS; Hedgecock 1994) dynamics, where a single recruitment year of surviving offspring comes from a subset of adults (Barfield et al. 2022), however the high recruitment from LTER 3 appears to be mediated by the MHW rather than normal SRS dynamics. LTERs 1 and 5 exhibited the greatest mortality following the 2019 MHW and were similarly the only sites to show a decrease in juvenile genetic diversity (Figure S7). In contrast, LTER 3 experienced the lowest heat stress (Figure 1A), bleaching, and mortality (Figure 1C,D), and exhibited no decrease in juvenile diversity compared to adults and was also the major source of recruits in 2021. As *A. hyacinthus* colonies that escaped bleaching demonstrate higher fecundity than those that bleached and recovered in Mo'orea (Leinbach et al. 2023), it seems likely that the observed recruitment dynamics were a result of the less intense stress experienced at LTER 3, leading to both higher colony survival and fecundity compared to the other sites. Information on the hydrodynamic processes around the island is limited; however, Leichter et al. (2013) inferred biological particles stay in relatively close proximity to the island over periods of days to weeks, via a current that moves over the entire island in a counterclockwise fashion. This suggests the recruitment patterns observed here (e.g., heavy recruitment from LTER 3 deep forereef and no recruitment from LTER 5 deep forereef; Figure 2D) are more a function of gamete production rather than oceanographic barriers and corridors. Our study does not include a non‐disturbance recruitment baseline, thus limiting our ability to determine whether the decrease of genetic diversity and individual heterozygosity in 2021 juveniles is reflective of normal recruitment patterns or a consequence of the MHWs in 2019 and 2020. Despite this, we show that spatially varying selection can strongly contribute to post‐disturbance recruitment dynamics and population reestablishment, emphasizing the importance of connectivity and sublethal effects on the ability of corals to rebound from MHWs.

#### 2.4.1 Host Shows Highly Polygenic Response to the Bleaching Event

To identify major effect loci that might be driving heat stress tolerance, two different genome‐wide association studies (GWAS) were performed: (1) for pre‐mortality samples taken during the bleaching event (*N* = 172) using health score (1–5) as a measurement of bleaching resistance (Figure 1C; Figure S13) and (2) for pre‐ and post‐mortality samples using timepoint (May 2019 or October 2019) as a binary trait for survival (*N* = 231) (see File S1 for all sample numbers and loci). General linear models (GLMs) were used to test for additive effects of SNPs on the quantile‐normalized health score (Zhou 2017; Zhou and Stephens 2012), including as covariates the first two genetic PCos, non‐genetic/environmental variables (surface area of the colony and the collection depth as continuous variables), and the proportion of *Symbiodinium* and *Cladocopium* reads (as two separate terms) relative to all symbiont reads. We find no significant differences in GWAS summary statistics when including versus excluding PCo1 and PCo2 (from a PCoA performed on all genomic loci with pre‐mortality and post‐mortality samples) as covariates (Figure S14), so we elected to keep these in our model. Through shuffled permutation tests, we determined conservative genome‐wide significance thresholds of *p* = 2.02e‐9 for health score in the pre‐mortality (May 2019) samples and *p* = 5.24e‐7 for survival in the pre‐mortality and post‐mortality samples (Figure S15). Using this approach, we failed to identify a single significant genetic locus (Figures S16 and S17). To verify if this result was driven by reef‐habitat specific responses, we also repeated this approach using only our most heavily sampled reef habitat—the deep forereef—yet still failed to identify significant loci (Figures S18 and S19). It is possible that with sampling more colonies or, for the survival GWAS, monitoring colonies through the bleaching event and scoring colonies as survivors/non‐survivors, we would be able to better detect large effect loci. Alternatively, bleaching resistance and survival might be polygenic traits driven by many loci of small effect.

Despite the stability in total genetic diversity across the MME, we noticed that within sites, samples were shifting slightly on PCos 1 and 2 (Figure S20) and global *F*_ST_ showed a small amount of structure between pre‐ and post‐mortality timepoints (Figure S39). If these shifts were occurring due to drift or sampling bias, we would expect these shifts to be completely random and thus have no temporal covariance between sites (Buffalo and Coop 2019). However, instead we identified a significant convergent shift in genome‐wide allele frequencies following the mortality event between most sites (Figure 3A) in support of a polygenic basis for bleaching survival. A polygenic signature of heat tolerance in *A. hyacinthus* here aligns with previous research on bleaching susceptibility in *A. hyacinthus* from American Samoa (Rose et al. 2018) and *A. millepora* in the GBR (Fuller et al. 2020). This signature of parallel polygenic selection suggests that there was a consistent selection pressure across many sites, with exceptions discussed in the subsequent section. The examples of convergence indicate many populations in Mo'orea share adaptive variants that are present at high frequencies (Barghi et al. 2020) or that there is background selection on shared deleterious variation (Buffalo and Coop 2020). The absence of convergence with comparisons involving LTER 2 deep forereef can be explained by the sampling design—a complete lack of live colonies at LTER 2 in October 2019 at 10 m necessitated sampling 5 m deeper than had been sampled in May 2019 (Table S1). Given this, our results suggest LTER 2 colonies at deeper depths either had different standing genetic variation and/or experienced differential selection pressures from the other reef sites. To account for this unique discrepancy, we removed these deeper samples from all GWAS analyses.

**FIGURE 3:**
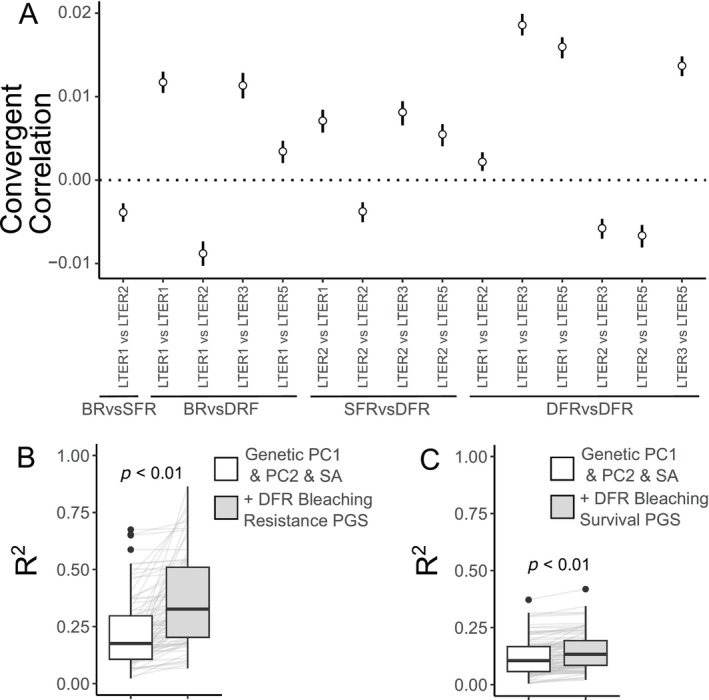
Bleaching resistance and survival have a host genetic basis. (A) Convergent correlation statistic for allele frequency shifts between pre‐ and post‐ mortality timepoints. Vertical lines are 97.5% confidence intervals from 100 bootstraps. (B) Distributions of test set *R* ^2^ values across 100 replicates (grey lines), comparing models with only genetic PCos 1 and 2 and SA (white) to models that also include the respective PGS (light grey): Deep forereef bleaching resistance PGS and (C) deep forereef bleaching survival PGS. BR, backreef; DFR, deep forereef; SA, surface area; SFR, shallow forereef.

#### 2.4.2 Reef‐Habitat‐Specific Responses to the Bleaching Event

Given our findings suggesting a polygenic basis for bleaching resistance and survival, we created a polygenic score (PGS) for both traits to test the feasibility of using a genomics‐based approach to predict individual responses to an MHW. Despite the strong convergence between many sites, there was a pattern suggesting corals from different reef habitats had unique responses. Reef‐habitat specific responses to a heat wave could be a major limitation on the use of a PGS for predicting resilient corals. We thus explored the impact of reef‐habitat specific responses by calculating PGSs that estimate the genetic basis of bleaching resistance (i.e., pre‐mortality samples taken during the bleaching event using health score [1–5] as a measurement of bleaching resistance) or survival (i.e., using timepoint [May 2019 or October 2019] as a binary trait for surviving the MME). We used either samples from all three reef habitats or those only from the deep forereef habitat, as it was the reef habitat with the most sites and individuals. This results in four PGSs: (1) bleaching resistance PGS, (2) bleaching survival PGS, (3) deep forereef bleaching resistance PGS, and (4) deep forereef bleaching survival PGS (see File S1 for samples included in each GWAS). For each PGS, we test predictability on a subset of samples held out from the training set used to generate the PGS (see “Methods” in Supporting Information). We use these four PGSs to demonstrate reef‐habitat specific responses to the bleaching event through three main approaches:

First, we assessed each of the four PGSs' power to predict bleaching resistance or survival. On their own, all PGSs offer very little predictability (mean *R* ^2^s: 0.01–0.04; Figure S21). This small but significant predictability offered by the PGS mirrors previous work on bleaching resistance PGSs in *Acropora* (Fuller et al. 2020) and while this predictability should increase with a higher sample size and depth of coverage, it echoes the many studies that have revealed the influence of factors outside of host genetics, for example, environmental variability, symbiont community etc. (reviewed in Dellaert and Putnam 2023), that can dictate variability in bleaching. While the deep forereef PGSs offered significant increases in predictability compared to randomly selected loci, the PGSs calculated using samples from all reef‐habitats did not (Figure S22). Given that both environmental and genetic factors contribute to variation in bleaching, we also explored whether the PGSs increase predictability of bleaching resistance and/or survival when accounting for other non‐genetic factors. To do so we combined each PGS with the same variables included as covariates in the GWAS, to assess whether including these parameters improved model prediction. We also ensure that any improvement in model prediction offered by the PGS is not due to overfitting by comparing the model's performance when we substitute the real PGS with a null PGS (i.e., randomly selected loci). We found that adding the respective PGS (bleaching resistance or bleaching survival) to a model with genetic PCos (PCo1 and PCo2), colony size, and depth significantly improved the prediction of both health score (*p* < 0.05) and bleaching survival (*p* < 0.05) but crucially, only for the deep forereef PGSs (Figure 3B,C). This increase in predictive ability was greater than that offered by null PGSs (Figure S23). We also ensured that these results were not driven by missing data artifacts (see “Results” in Supporting Information; Figure S24) and explored adding symbiont communities to the model (see “Results” in Supporting Information; Figures S25 and S26).

Second, we observe a lack of convergent evolution between backreef and shallow forereef populations both genome‐wide (Figure 3A) and at the loci for the deep forereef bleaching survival PGS (Figure S27), suggesting that selection varied spatially across these distinct reef zones. There is also a much stronger signal of convergent evolution among deep forereef populations than between deep forereef and non‐deep forereef populations. This is true both genome‐wide (Figure 3A) and at the loci for the deep forereef bleaching survival PGS (Figure S27). Finally, a deep forereef bleaching survival PGS appears to offer no increase in predictive power for determining survivorship in backreef individuals (i.e., a PGS built using deep forereef individuals only for the training set with survival as the trait and then using backreef individuals as the test set to assess predictability; see “Methods” in Supporting Information) compared to PGSs built from randomly selected loci (Figure S28).

Our identification of reef‐habitat specificity is likely due to spatially divergent selection pressures across reef habitats, further supported by the divergence in symbiont populations between these environments (Figure 5A,B). Thus, while there is a parallel signature within the deep forereef sites, the 2019 MHW did not impose a single homogenous selective pressure across corals spanning backreef to forereef habitats. Many other abiotic and biotic factors covary to varying degrees with temperature; for example, the shallow forereef experiences higher UV irradiance in comparison to the deeper forereef habitat (Dubé et al. 2023), which can impact Symbiodiniaceae physiology by different mechanisms compared to heat stress (Downs et al. 2013). These diverse environments likely result in variable selective pressures across habitats, each imposing unique influences on the coral genome. It is also possible that there is a high degree of genetic redundancy for bleaching resistance and survival, and reef‐habitat specific shifts are due to differences in standing genetic variation. This standing genetic variation hypothesis is supported in part by the lack of a convergent shift in allele frequencies between backreef and shallow forereef habitats, which also happens to be the comparison with the greatest degree of genetic structure (Figure S3). These results suggest a genomic basis for MHW responses and build on previous genomic predictions of bleaching (Fuller et al. 2020) by extending them to MHW survival. However, our findings also demonstrate the challenges in applying a PGS across reef‐habitats.

#### 2.4.3 Signatures of Selection and Identification of Adaptive Variants in the Next Generation Following an MME Suggest Rapid Evolution

**FIGURE 4:**
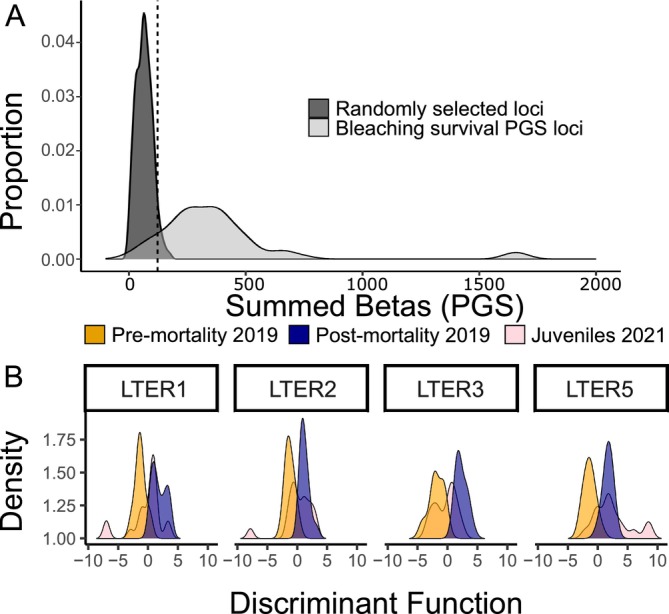
Juveniles maintain divergence in loci that predict bleaching survival. (A) Distribution of summed beta scores from pre‐mortality versus juveniles GWAS for both 500 sets of randomly selected loci (dark grey) and the loci used for the deep forereef bleaching survival PGS (Figure 3C; light grey). Vertical line shows 95th percentile for the randomly selected loci. (B) DAPC results for the loci significantly differentiated between timepoints per GWAS, demonstrating the intermediate status of juvenile samples.

To test whether allele frequency shifts resulting from the MME are maintained in subsequent generations, we examined the effect sizes of a pre‐mortality (deep forereef only) versus juvenile GWAS at the roughly 150,000 loci from the deep forereef bleaching survival PGS in Figure 3C. We use the deep forereef bleaching survival PGS because of its predictive power and because we only sampled juveniles from deep forereef locations. We find the sum of effect sizes at these loci was higher than randomly selected loci (Figure 4A), demonstrating these loci were also important for explaining pre‐mortality vs. juvenile genetic differentiation. We then used DAPC analyses for the ~150,000 loci in the bleaching survival PGS to investigate whether juveniles maintain the degree of divergence at these loci or if allele frequencies return towards pre‐mortality levels. DAPC results using pre‐mortality and post‐mortality samples as a training dataset show that juveniles represented an intermediate point between the two timepoints on LD1 (Figure 4B; Figure S29). When naively assigning juveniles to either pre‐ or post‐mortality groupings, 32/74 juveniles were assigned to the pre‐mortality group and 42/74 assigned to the post‐mortality group (Figure S29).

One possible interpretation of this result is that the allele frequency shifts caused by the MME are mitigated in the following generation, either through genetic drift or an opposing selective pressure (e.g., competition for space). Alternatively, it is possible that this “intermediate frequency” in the following generation is actually the expected outcome under a scenario of rapid adaptive evolution if we consider the quantitative genetics model used here to capture “outlier loci” between pre‐ and post‐ mortality timepoints will contain false positives due to sampling bias from not capturing the entire population. To test this, we ran adaptive evolution simulations in a population under our sampling scheme and show that we can indeed recreate this intermediate frequency in outlier loci in the generation after an MME (Figure S30). This provides evidence for rapid adaptive evolution in this population and that the shift in juveniles occurs at the subset of “outlier loci” that likely represent true adaptive loci for surviving the MME.

Another possible interpretation is that these putatively adaptive loci are only shifting due to the high recruitment from LTER 3, the location that experienced the least heat stress (Figure 2D). In other words, under this interpretation, allele frequency shifts could be driven by an influx of genotypes not exposed to the same severity of heat stress. This hypothesis is refuted by our data in two ways: (1) these adaptive genetic loci exhibit a convergent shift among all deep forereef sites following the MME (Figure S27) before there is a chance of recruitment, which we would not expect if shifts were due to genetic drift (Buffalo and Coop 2020) and (2) LTER 3 at the pre‐mortality timepoint should also show the intermediate frequency observed in juveniles (i.e., cluster with juveniles rather than other pre‐mortality timepoint samples from other sites) if shifts are simply due to recruitment from LTER 3, but this pattern was not observed (Figure S31).

As *A. hyacinthus* spawns in November, age estimates from tabular *Acropora* growth data (Wallace 1985; Yap et al. 2013) indicate these juveniles are too small to be 3+ years old and thus we believe they would not have experienced the 2019 MHW. However, while these juveniles likely represent recruitment from survivors of the 2019 MHW, the rapid evolution observed here could be a combination of selective pressure from the 2019 MHW on the previous generation and selection pressure from the 2020 MHW on the juveniles. Regardless of the mechanism, we find evidence that MHWs impose allele frequency shifts that persist across generations, suggestive of directional selection favoring variants that enhance thermal tolerance.

Our adaptive evolution simulations (Figure S30) suggest that the loci exhibiting divergence in the juveniles compared to the pre‐mortality timepoint may provide a better representation of the true adaptive loci for this MME. Given that GWAS effect sizes are obtained as a noisy combination of the unobserved true SNP effect sizes (Hormozdiari et al. 2014; Zhu and Stephens 2017), identifying true adaptive loci by taking the overlap of a GWAS between pre‐ and post‐mortality and a second GWAS between pre‐mortality and juveniles (both using deep forereef sites only) is likely overly conservative. However, this approach should capture the most important putative loci for adaptation. Given this approach and setting a 0.01 *p*‐value threshold resulted in 316 loci (File S1), none of them projected to be of major effect. Of the overlapping loci with the highest signal (i.e., *p* < 0.001) in the pre‐mortality versus juvenile GWAS, we found several loci within gene regions with previously described functions in cnidarians including Transcription factor AP2 (TFAP2A), Notch receptor 2 (NOTCH2), and NF‐kappa‐B inhibitor‐interacting Ras 2 (NKIRAS2). TFAP2A is essential for germ cell commitment and gonad development in cnidarians (DuBuc et al. 2020). NOTCH2 is important during development for cell fate determination, neurogenesis, and for establishing tissue boundaries during the budding of new polyps in cnidarians (Käsbauer et al. 2007; Marlow et al. 2012; Münder et al. 2010). NKIRAS2 inhibits NFkB activation (Tago et al. 2010) which is a transcription factor involved in stress response and innate immunity across the metazoan tree of life (Gilmore and Wolenski 2012; Weis 2019). These genes are candidates for future functional research on coral bleaching.

#### 2.4.4 Local Adaptation of *Symbiodinium* and *Cladocopium* Between Shallow and Deep Forereef Sites

**FIGURE 5:**
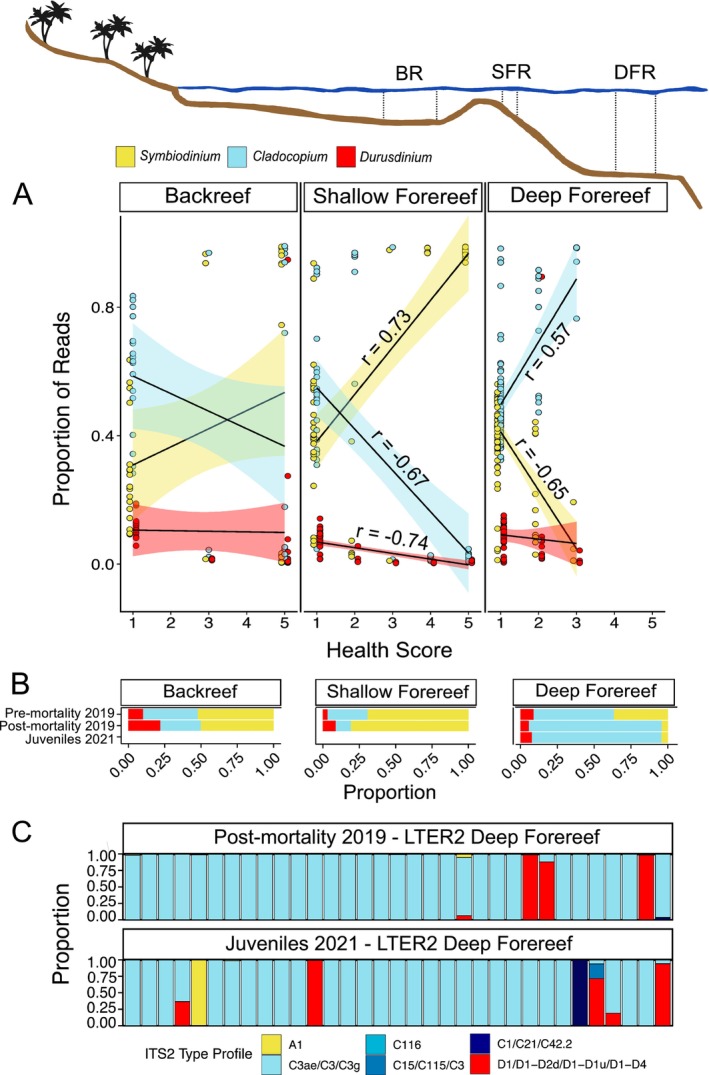
Algal symbiont divergence between reef zones contributes to bleaching response. (A) Relationships between health score (1 indicates complete bleaching and 5 indicates no pigmentation loss) (Photo examples in Figure S13) and Symbiodiniaceae genera during the bleaching event (i.e., pre‐mortality May 2019) at backreef, shallow forereef and deep forereef habitats. Each dot represents the relative proportion of mapped reads to the three Symbiodiniaceae genera. Black lines show fitted linear regression with 95% confidence intervals. For significant regressions, *r* values show Pearson correlation coefficient. (B) Proportion of WGS reads mapped to each of the three Symbiodiniaceae genera (*Symbiodinium*, *Cladocopium*, and *Durusdinium*) for each site across timepoints at backreef, shallow forereef, and deep forereef habitats. (C) Proportion of collapsed ITS2 type profiles for individual colonies at LTER 2 deep forereef for both 2019 post‐mortality (Leinbach et al. 2023) and 2021 timepoints.

WGS reads mapping to the genomes of three Symbiodiniaceae genera (*Symbiodinium*, *Cladocopium*, and *Durusdinium*) were used to approximate the proportion of symbionts in each sample. We found no evidence for any associations with *Breviolum* through additional ITS2 sequencing (Leinbach et al. 2023) so it was left out of our analysis. It is important to note that our “pre‐mortality” timepoint occurs during the bleaching event and therefore does not reflect the symbiont community prior to heat stress. This has two major implications for interpreting our results: (1) It is unclear whether the environmental structuring of symbiont communities described below is observed year‐round or is specific to the heat stress event (see Leinbach et al. 2023 for a detailed discussion), (2) bleached corals across habitats generally exhibited more mixed communities compared to healthy corals (Figure 5A). However, the cause‐effect relationship for the bleached corals (i.e., low‐health corals) is not clear—in other words, it is uncertain whether the observed symbiont community structure caused the bleaching or is a symptom of dysbiosis. Despite this uncertainty, bleached corals still provide valuable insights into the presence of specific symbiont genera at each reef habitat. Pairing this information alongside symbiont communities sampled from healthy corals during the bleaching event and from surviving corals post‐bleaching allows us to investigate whether a particular symbiont genus might be locally adaptive.

We find evidence for locally adaptive symbiont genera between the shallow and deep forereefs. At shallow forereef sites (3–5 m), increased *Symbiodinium* reads were associated with lower bleaching susceptibility (i.e., higher health score; *p* < 0.0001; *r* = 0.73), whereas increased *Cladocopium* reads were associated with higher bleaching susceptibility (i.e., lower health score; *p* < 0.0001; *r* = −0.67; Figure 5A). These results are consistent with characterizations of symbiont communities using ITS2 amplicon sequencing from recovered and resistant colonies at the LTER 2 location (Leinbach et al. 2023). However, the deep forereef showed the opposite trend, where an increased abundance of *Cladocopium* reads was associated with lower bleaching susceptibility (*p* < 0.0001; *r* = 0.57), while an increased abundance of *Symbiodinium* reads was associated with higher bleaching susceptibility (*p* < 0.0001; *r* = −0.65; Figure 5A). The deep forereef showed changes in the proportion of Symbiodiniaceae genera between timepoints, with an increase in the proportion of *Cladocopium* post‐mortality (October 2019) compared to pre‐mortality (May 2019), and a corresponding decrease of both *Symbiodinium* and *Durusdinium* (*p* < 0.0001; Figure 5B). GLMs looking at the interaction between reef habitat and symbiont proportions, while controlling for the effect of colony size and LTER location, also show a positive relationship between *Cladocopium* reads and health score (*p* < 0.0001; File S1) at the deep forereef relative to the shallow forereef and a negative relationship between *Symbiodinium* reads and health score (*p* < 0.0001; File S1) at the deep forereef relative to shallow forereef.

It is possible that symbiont zonation could be related to host genetic structuring (Bongaerts et al. 2010; Brazeau et al. 2013), however, this reef system has high gene flow between habitats and little population structure, as shown in this study (Figure 2A) and in Kriefall et al. (2022) so this explanation seems unlikely. This is further supported by results from a redundancy analysis (RDA) where host genetic structure was not explained by symbiont dominance (*Symbiodinium: p* = 0.614, *R* ^2^ < 0.01; *Cladocopium*: *p* = 0.638, *R* ^2^ < 0.01; *Durusdinium*: *p* = 0.434, *R* ^2^ < 0.01; Table S2). We also show the reverse, that symbiont dominance is not explained by host genetic structure (*p* = 0.78, *R* ^2^ < 0.01), pointing to a lack of host‐driven symbiont structuring. Alternatively, reef habitat is predictive of symbiont structuring (*p* < 0.05, *R* ^2^ = 0.14; Table S3), consistent with the results from our GLMs. Strong environmental structuring of symbiont communities, rather than the host, might be expected here because *A. hyacinthus* is a horizontally transmitting broadcast spawner. However, we also note that, to our knowledge, no study has demonstrated intraspecific host genetic structuring of symbiont communities, except when the coral host has highly diverged populations (Bongaerts et al. 2010; Brazeau et al. 2013).

#### 2.4.5 Maintenance of Symbiont Community Structure 2 Years After the Heat Stress Event

The increase of *Cladocopium* dominance on the deep forereef was maintained in 2019 juveniles, which exhibited no significant shifts in *Cladocopium* dominance from the post‐bleaching October 2019 timepoint (*p* = 0.09, Figure 5B). As there has never been information collected on the symbiont communities of *A. hyacinthus* from the deep forereef prior to the bleaching event in May 2019, we do not know if this structure maintains a post‐bleaching shift or reflects historical structure. ITS2 amplicon sequencing was carried out on a subset of samples (see Table S1) to offer finer taxonomic resolution to supplement the broader genus‐level classifications from the WGS data. ITS2 data from collected juveniles in this study show that *A. hyacinthus* at all deep forereef sites are dominated by *Cladocopium* C3ae, with occasional exceptions of certain colonies with multiple symbiont associations and a few dominated by A1, C1, and D1 (Figure 5C; Figure S32). We observe a negative correlation between juvenile colony size and ITS2 type richness (*p* < 0.05; Figure S33), indicative of symbiont winnowing (Abrego et al. 2009). However, the vast majority of juveniles sampled (96/115, 83.48%) were dominated by C3ae and we observed no differences in symbiont community composition and diversity between post mortality and juvenile timepoints (*p* = 0.825, Figure S34). *Cladocopium* C3ae is not prevalent in *A. hyacinthus* outside of Mo'orea, with the C3ae variant only appearing once in the Cook Islands (Lewis et al. 2022). However, without more comprehensive sampling at other islands, it is difficult to know whether the C3ae variant here is truly endemic to Mo'orea.

#### 2.4.6 No Evidence for an Adaptive Role of *Durusdinium*

Despite the presence of *Durusdinium* in corals from all reef habitats, we find no evidence that corals escaped or recovered from bleaching by shifting to *Durusdinium* dominance. This result contrasts the large body of observations that *Durusdinium* (formerly *Symbiodinium* Clade D) is associated with higher thermal tolerance across diverse coral genera (Berkelmans and Van Oppen 2006; Fuller et al. 2020; Klueter et al. 2017; LaJeunesse et al. 2018; Morikawa and Palumbi 2019). In our study, *Durusdinium* was only correlated with health score at the shallow forereef, where a higher proportion was associated with more bleaching (*p* < 0.05, *r* = −0.74; Figure 5A). It is unlikely that the *Durusdinium* we observe in *A. hyacinthus* is a novel, heat‐susceptible species of *Durusdinium*, as ITS2 symbiont profiles for juvenile coral samples (Figure 5C) and a subset of adult samples (Leinbach et al. 2023) contain both the D1 and D4 sequences characteristic of *D. trenchii* (Hume et al. 2020), which has previously been linked to bleaching resistance (Silverstein et al. 2017). Rather, it seems that we have found another rare exception to the rule of *Durusdinium* conferring holobiont heat tolerance (Abrego et al. 2008; Howe‐Kerr et al. 2020; Howells et al. 2020). As stated previously, because data were generated from some severely bleached colonies, it is possible that the symbiont structure we observed pre‐mortality (May 2019) is not indicative of the structure during or prior to the heat stress, but rather reflects symbionts that remained or were taken up once the colony bleached. However, if hosting *Durusdinium* was adaptive here, we would expect *Durusdinium* to increase in relative abundance from pre‐ to post‐mortality timepoints, which we did not observe. In fact, at the deep forereef, we record a decrease in *Durusdinium* from pre‐ to post‐mortality timepoints (*p* < 0.0001, Figure 5B).

## 3 Discussion

Since the 1970s, coral reef populations in Mo'orea have experienced multiple cycles of disturbance and recovery (Adjeroud et al. 2009; Holbrook et al. 2018). These disturbance regimes, which include outbreaks of the corallivorous seastar *Acanthaster planci* in 1980–1982 (Berumen and Pratchett 2006) and 2006–2009 (Kayal et al. 2012), cyclones in 1983 (Harmelin‐Vivien and Laboute 1986), 1991 (Adjeroud et al. 2002), and 2010 (Kayal et al. 2012), and several heat stress events (Hoegh‐Guldberg et al. 2007; Penin et al. 2007), were then followed by the return of coral cover to pre‐disturbance levels within 8–10 years (Adjeroud et al. 2009; Holbrook et al. 2018).

The Mo'orean *A. hyacinthus* populations allow us to address an important question facing coral reefs in the Anthropocene: how will coral populations rapidly adapt to disturbances when they have already exhausted conventional sources of adaptive variation? In other locations in the South Pacific, recovery of *A. hyacinthus* is thought to depend on a particularly heat resilient genetic lineage within the *A. hyacinthus* species complex (Rose et al. 2021), or availability of the most heat resilient symbionts (e.g., *Durusdinium*; Oliver and Palumbi 2011). However, in Mo'orea this *A. hyacinthus* population only consists of corals from the “heat resilient” HE *A. hyacinthus* genetic lineage (Rose et al. 2021) and although the symbiont *Durusdinium* is present, it fails to impact survival. We find instead that survival can be predicted through the small effect of multiple alleles that are maintained in the following generation, providing evidence for contemporary adaptation to rising temperatures.

Our data show there is a polygenic basis for bleaching in the host, in line with previous work (Dixon et al. 2015; Drury et al. 2022; Fuller et al. 2020; Kirk et al. 2018; Rose et al. 2018, 2021). Additionally, we find a polygenic basis for survival from the bleaching event and that the genetic variation that predicts bleaching survival is maintained in juveniles 2 years later. This provides evidence for rapid evolution in response to the 2019 MHW and may in part explain why there was no further decline in *Acropora* coral cover following the 2020 MHW. While the contribution to bleaching predictability might be small, the finding that intraspecific host genetic variation impacts variation in mortality at all is significant. It is important to note that parameters such as size, depth and symbiont genera are relatively static compared to host genetic variation. If a population's survival is limited to protection offered by non‐genetic factors, this imposes a lower ceiling on the ability to persist in the face of continuous stressors. Coral populations are not predicted to survive without adaptation driven by the host (Matz et al. 2020).

This MME did not result in decreased genetic diversity in the short‐term and the stability of genetic diversity, in combination with high local larval retention (Leichter et al. 2013), could explain the rapid returns to baseline coral cover following disturbances consistently observed in Mo'orea. There are many different biological factors that can explain a lag in observing a decrease in genetic diversity following environmental deterioration (reviewed in Gargiulo et al. 2025). Most of these mechanisms rely on short‐term persistence of most individuals, which did not occur here as evidenced by the high (~50%–80%) mortality rates. One remaining mitigating factor is large *N*_e_, which is observed in our populations of *A. hyacinthus* , likely due to their extensive Indo‐Pacific range (Ladner and Palumbi 2012). While large *N*_e_ seems to have mitigated genetic diversity loss in the short term here, it remains to be seen if genetic diversity will be maintained if subsequent generations experience increased frequency of disturbance events. This is an extremely limited area of study in coral. The only other study to date reported stable genetic diversity in *A. spicifera* following a large disturbance, attributing this pattern to spatial variability in disturbance intensity (Thomas et al. 2024). To some extent, we also see spatial variability of disturbance intensity in Mo'orea. We find the sites with the highest mortality show lower genetic diversity in 2021 juveniles, while the site that experienced the lowest heat stress, bleaching, and mortality (LTER 3) served as the largest source for juvenile recruitment and had similar levels of genetic diversity in 2021 juveniles compared to pre‐ and post‐mortality adult populations. This suggests that MMEs can alter recruitment dynamics simply through spatial variation in stressor severity and coral genetic diversity can be robust to single instances of large disturbances, but studies are needed across a broader diversity of Scleractinians to extrapolate these findings to coral populations more generally.

Despite the encouraging evidence of a population evolving rather than perishing in the face of MHWs, there are two points that should temper expectations. First, it is important to note that even if adaptive responses occur in response to MMEs, population extinction or depletion to the point of extinction debt may still result. As environmental changes continue and accelerate, mean population phenotypes can increasingly lag behind the optimal phenotype, leading to an increasing burden of selective deaths (Hansen et al. 2012). Second, while a PGS has previously been proposed to provide a means to develop predictive models of bleaching for distinguishing tolerant individuals across different shelf positions, latitudes, and environmental conditions (Fuller et al. 2020), we find evidence for localized selective pressures having disparate impacts on allele frequency shifts. The habitat‐specific accuracy of our PGS and the breakdown in paralellism that occurs from sampling just a few meters deeper on the deep forereef demonstrates the difficulty in applying a PGS across diverse datasets and populations. This finding is consistent with existing evidence for microgeographical adaptation in corals (i.e., local adaptation occurring at the fine spatial scales at which populations should experience high gene flow based on expected levels of dispersal; Richardson et al. 2014), as different taxa have repeatedly shown adaptive divergence between different depths or reef zones well within their dispersal limits (Grupstra et al. 2024). We also note that host genetic variation offers relatively small increases in predictability for bleaching and survival compared to symbiont associations and other non‐genetic factors. This is not to say that this approach cannot lead to a robust predictive model, but rather emphasizes the need to thoroughly sample across habitats and populations and that models should not substitute genetic variation for important non‐genetic factors when attempting to optimize bleaching predictions.

We confirm previous findings of *A. hyacinthus* bleaching resistance mediated through hosting environment‐specific symbiont types (Leinbach et al. 2023) and further document a complete role reversal of resilient symbiont partners between shallow forereef and deep forereef sites. This stark difference in environment‐specific responses to a heat stress event between different algal partners in a panmictic host population suggests symbionts are adaptive in one environment and maladaptive in the other. It is possible that the local specialization is driven in part by the differences in light intensity between habitats (Dubé et al. 2021), as this depth‐related relationship between *Symbiodinium* and *Cladocopium* has previously been shown (Varasteh et al. 2022). The benefit of *Symbiodinium* at the shallower forereef might be due to their ability to efficiently synthesize photoprotectors as mycosporine‐like amino acids (MAAs; Silva‐Lima et al. 2015) and low amounts of hydrogen peroxide, a putative agent of coral bleaching, at elevated temperatures (Banaszak et al. 2000). While symbiont tradeoffs are often framed as one symbiont species being beneficial under environmental stress and another more beneficial when the stress has dissipated (e.g., Jones and Berkelmans 2010), here we show trade‐offs do not have to be limited in this way. Selection on symbiont‐environment associations can occur in opposing directions during the same heat stress event in different reef habitats.

Our temporal genomic approach reveals key insights into resilience mechanisms within coral populations amidst MMEs. We show evidence for discrete adaptive roles of symbiont types in distinct reef habitats, and the interplay between host and symbiont genetics, highlighting the complexity of coral survival strategies. Our PGS and sampling approach provides novel insight into the genetic architecture of MME survival and demonstrates that selective pressures vary both spatially (i.e., across habitats) and temporally (i.e., during stressor and recovery phases of the MME). Lastly, we document adaptation to warming temperatures from standing within‐species genetic variation. These findings highlight the intricate nature of selective pressures of MMEs, offering crucial insights into coral resilience and adaptation under ongoing, unprecedented warming.

## 4 Methods

### 4.1 Coral Collections

Samples were collected via SCUBA from *A. hyacinthus* colonies surrounding the island of Mo'orea as follows: (1) adults during the May 2019 bleaching event (pre‐mortality; *N* = 172), (2) adults after bleaching and thermal stress had subsided in October 2019 (post‐mortality; *N* = 103), and (3) juveniles in November 2021 (*N* = 115). Sampling in May was carried out to maximally capture the entire bleaching phenotypic landscape, with an effort to sample bleached and unbleached colonies at sites whenever possible. However, bleaching varied across sites, so it was not possible to sample equal numbers of corals across the bleaching spectrum. At the time of sampling in May, we did not observe the onset of mortality in any of the corals, despite many in a severely bleached state. Therefore, we strongly suspect we sampled prior to the onset of any mortality. Ten colonies were repeatedly sampled at both May 2019 and October 2019 timepoints.

Colony collections within each LTER spanned three habitat types where possible—backreef, shallow forereef, and deep forereef—distributed across depths of 1–3 m, 3–5 m, 10–14 m, respectively. Both May and October 2019 adult samples included all habitats, whereas the November 2021 juvenile cohort was sampled exclusively from the deep forereef. Following clone removal, this yielded 19 LTER × habitat × timepoint groupings for WGS analyses, each containing between 12 and 26 individuals (Table S1). See Supporting Information for information on temperature data (Figures S35 and S36).

### 4.2 Whole Genome Sequencing (WGS)

DNA was isolated using a modified phenol‐chloroform extraction method (Davies et al. 2013) and then cleaned with DNA Clean and Concentrator kits (Zymo, Irvine, CA, USA). Cleaned genomic DNA was sent to the University of California Davis Genome Center for library preparations using pooled Super‐High‐Throughput Shotgun sequencing for shallow WGS. Libraries were sequenced on Illumina NovaSeq 6000 S4 flow cells using two lanes of paired‐end 150‐bp and one lane using paired‐end 100‐bp. Adapters were trimmed and reads were filtered using *fastp* (Chen et al. 2018) (Phred scores ≥ *Q*20 and 40% unqualified reads threshold). Contamination from symbiotic DNA was filtered out by mapping trimmed reads to a concatenated genome of four Symbiodiniaceae genera: *Symbiodinium* (Aranda et al. 2016), *Breviolum* (Shoguchi et al. 2013), *Cladocopium* (H. Liu et al. 2018), and *Durusdinium* (Dougan et al. 2022) via bowtie2's (Langmead and Salzberg 2013) ‐‐un‐conc option. Symbiont‐free paired‐end reads were mapped to the *A. hyacinthus* genome (López‐Nandam et al. 2023) using bowtie2 (parameters ‐I 0 ‐X 1500 ‐‐no‐unal –fr), resulting in an average mapping rate of 88.4% to the coral genome (File S1). Aligned reads were then merged across the three lanes using samtools (Li et al. 2009). The *clipOverlap* function of bamUtil (Jun et al. 2015) clipped overlapping read pairs and PCR duplicates were removed using *MarkDuplicates* in Picard (2019), resulting in mean 6× coverage (minimum 1.7× coverage; File S1). Genotyping and filtering of SNPs (“Methods” in File S1 for analysis specific filtering) were performed using ANGSD (Korneliussen et al. 2014).

### 4.3 Clone Identification and Genetic Structure Between Sites, Habitats, and Timepoints

Clones were detected using hierarchical clustering of samples based on pairwise identity by state (IBS) distances calculated in ANGSD. Technical replicates were used to identify appropriate height cutoffs for clone identification (see “Results” in Supporting Information; Figure S38). We used ngsRelate (Korneliussen et al. 2014) to identify putative siblings and half‐siblings (using a < 0.25 relatedness coefficient cutoff), which we removed from population structure analyses to avoid related pairs driving variation along PCos (see Table S1 for individuals removed). To compare population structure between sites, habitats, and timepoints, PCoAs and DAPCs were performed via a covariance matrix based on single‐read resampling calculated in ANGSD. Additionally, DAPCs were used to identify potential source populations for juveniles. To do so, DAPCs were performed using adult colonies from 2019 (both pre‐ and post‐mortality) as training data. DAPCs were conditioned on site (irrespective of year) and the resulting model was used to predict juvenile site assignment. Population structure was also assessed using NGSadmix (Skotte et al. 2013) and optimal *K* using the Evanno method (Evanno et al. 2005). PCoAs incorporating WGS data from Rose et al. (2021) were used to confirm that all samples were *A. hyacinthus*; genotypes were called using the same set of ANGSD filters as those used for the Mo'orea samples (described in “Methods” in Supporting Information).

Expected heterozygosity, nucleotide diversity (Watterson's *θ*) and *π* were calculated from each individual's site frequency spectrum (SFS) (dividing singletons by all loci) generated by first calculating the site allele frequency (SAF) in ANGSD with no MAF filter and then the unfolded (using the *A. hyacinthus* reference genome; López‐Nandam et al. 2023 as an ancestral reference) SFS in winsfs (Rassmussen et al. 2025). Differences in expected heterozygosity, nucleotide diversity and *π* between sites and timepoints were calculated via Dunn's test (1964) with a Benjamini–Hochberg multiple test correction. To examine genetic differentiation between sites, SFSs were used as priors with the SAF to calculate genome‐wide *F*_ST_. To determine how large *A. hyacinthus* *N*_e_ was compared to other *A. hyacinthus* populations, GONE (Santiago et al. 2020) was used to model *N*_e_ through time. Unfolded SFSs for *N*_e_ estimates were generated by first identifying a set of putatively unlinked loci through linkage disequilibrium (LD) pruning in PLINK2 and using a per generation mutation rate of 2e‐8 and a generation time of 5 (Fifer et al. 2022).

### 4.4 Genome‐Wide Association Study (GWAS) on Variation in Bleaching Resistance in Pre‐Mortality Samples and Bleaching Survival in Pre‐ and Post‐Mortality Samples

A GWAS for bleaching resistance was performed for pre‐mortality samples taken during the bleaching event (*N* = 172), with health score as the trait, serving as a proxy for bleaching resistance (Figure 1C). General linear models (GLMs) were used to test for additive effects of SNPs on the quantile‐normalized health score (Zhou 2017; Zhou and Stephens 2012), including as covariates the first two genetic PCos, non‐genetic/environmental variables (surface area of the colony and the collection depth as continuous variables), and the proportion of *Symbiodinium* and *Cladocopium* reads (as two separate terms) relative to all symbiont reads. Reads mapping to three Symbiodiniaceae genera genomes (i.e., *Symbiodinium*, *Cladocopium*, and *Durusdinium*) were used as an approximation of the proportion of symbionts in each sample following Fuller et al. (2020). For the GLM, a GWAS was performed using a standard linear regression as implemented in PLINK2. Because PLINK2 requires hard calling genotypes we added additional filters (“Methods” in Supporting Information). Genome‐wide cutoffs were determined through 10,000 permutations of the GLM, randomly shuffling the trait values, extracting the minimum *p*‐value for each run, and then taking the value of the 95th percentile of this distribution (Fuller et al. 2020).

A GWAS was also carried out assigning the pre‐ and post‐mortality timepoints as a binary trait (i.e., bleaching survival) to calculate a GWAS for bleaching survival. We repeated both the bleaching resistance and bleaching survival GWAS, subsetting the data to include adults from the deep forereef only. We were unable to perform GWAS on other reef habitat subsets due to low sample size (Table S1). For subsets, depth was removed as a covariate as depth was constant within subsets. Information on which samples were included in each GWAS is available in File S1.

### 4.5 Investigating Convergent Signature of Selection Following Mortality Event

We leveraged the temporal sampling at multiple sites to examine covariance in allele frequency shifts following the mortality event. If shifts are caused by a common selective pressure, we expect to observe concordance between sites, whereas discordance between sites is reflective of genetic drift, opposing selection pressures, or differences in standing genetic variation (Buffalo and Coop 2020). To assess evidence for parallel adaptation, we used the “convergent correlation” statistic described by Buffalo and Coop (2020). This takes allele frequency shifts between timepoints at each site throughout the entire genome and assesses correlation using a Pearson correlation test. We estimated 95% bootstrap confidence intervals by resampling the loci used to calculate each convergent correlation statistic 1000 times with replacement following Reid et al. (2023).

### 4.6 Calculation of Bleaching Resistance and Bleaching Survival Polygenic Scores (PGSs)

We calculated four polygenic scores (PGSs) in total: bleaching resistance PGS, bleaching survival PGS, deep forereef bleaching resistance PGS and deep forereef bleaching survival PGS (details of data partitioning and sample sizes included in Table S1). For the deep forereef PGSs we only used samples from the deep forereef. All PGSs were calculated using a jackknife cross validation procedure (Fuller et al. 2020). Samples were subset into 100 partitions of training and test sets, withholding 15% of individuals for the test and using the rest (85%) as the training set. For each training set, a GWAS (using either the GLM or LM as described above) was performed. *p*‐value thresholding was used to build the PGS followed by LD‐clumping via PLINK to find approximately independent SNPs. For each test set, the PGS was calculated by summing the allelic dosages weighted by the estimated effect size. PGS accuracy of different *p*‐value thresholds was measured by taking *R* ^2^ from a linear model run with PGS as the sole parameter and then proceeding with the *p*‐value that maximized *R* ^2^ (Figure S16). We further assessed the performance of the PGS on its own by comparing the distribution of *R* ^2^s in the 100 test sets with *R* ^2^s from a null PGS calculated by randomly selecting loci. We then tested whether the real PGS added significant predictability compared to the null PGS using Mann–Whitney *U* tests with an FDR correction to account for our subset comparisons.

Given that bleaching resistance and survival are likely influenced by a combination of genetic and non‐genetic factors (Fuller et al. 2020) we do not necessarily expect the PGS to increase predictability on its own. Instead, it is more meaningful to assess whether a model with important non‐genetic predictors of bleaching is improved when adding the PGS. We did so by assessing the change in *R* ^2^ between linear models with different combinations of the PGS and covariates using Mann–Whitney *U* tests (with an FDR correction to account for the multiple PGSs subsetting the same data), with the expectation that the PGS would increase the predictability of health score and survival. In instances where the inclusion of the PGS increased predictability, we again tested whether including the PGS added significant predictability compared to including a corresponding null PGS.

To follow up on habitat‐specific differences we examined whether the deep forereef bleaching survival PGS could predict survival at the backreef. We took beta values calculated from the deep forereef bleaching survival PGS and applied them in an additive model to generate a score for backreef samples. We then calculated the *R* ^2^ using a linear model to see how well the PGS predicted survival at the backreef and compared this *R* ^2^ to the distribution of *R* ^2^s from 500 sets of randomly selected loci. We repeated this with several different sets of loci and associated beta values for three different *p*‐value thresholds for the deep forereef bleaching survival PGS (0.01, 0.001, 0.0001).

### 4.7 Assessing the Distribution of Adaptive Variants in the Next Generation Following a Mortality Event

To test whether allele frequency shifts resulting from the MME were maintained in subsequent generations, we first examined the effect sizes of a pre‐mortality (using deep forereef sites only) versus juvenile GWAS at the ~150,000 loci (before LD clumping) from the deep forereef bleaching survival PGS described above. The effect sizes at these loci were compared to 500 sets of 150,000 randomly selected loci and a 95th percentile cutoff was used to determine significant deviation from this null distribution. To determine if allele frequencies after selection were maintained in the following generation (2021 juveniles), we performed PCoAs using the set of high signal loci identified by the deep forereef bleaching survival PGS. For these PCoAs, we generated covariance matrices using samples from all three timepoints across the sites that were common between all timepoints (i.e., the deep forereef sites). We also performed DAPCs on the PCoA table conditioned on site. For the DAPC analysis, juveniles were excluded from the training data and then assigned to either timepoint using the model to measure greater proximity to either pre‐ or post‐mortality groups.

To determine the expected distribution of allele frequency shifts in high signal loci from the deep forereef bleaching survival GWAS between timepoints (and compare with DAPCs above), simulations were performed, iterating over several values of population size and false positive rates. To simulate genotypes, random numbers were drawn from a binomial distribution with a probability of 0.1 per alternative allele for both simulated neutral and adaptive loci. Individuals in the top 30% of polygenic scores (to reflect ~30% survival rates; Figure 1D) were then randomly selected (to reflect subsampling of the true population) and a logistic regression between the simulated pre‐ and post‐mortality timepoints (as carried out in the GWAS) was conducted to identify putative outlier loci. These outlier loci contain true adaptive loci (i.e., adaptive in the whole population) or false positives (identified as outliers due to subsampling of the whole population). For each individual, a binomial distribution was again used to randomly assign alleles from the surviving genotypes for the next generation. We then visualized the distribution of these loci across both pre‐, post‐mortality, and juvenile timepoints.

To identify putatively adaptive loci that maintained post‐mortality allele frequencies in the subsequent generation we carried out another GWAS, this time assigning the pre‐mortality (but using only deep forereef sites) and juvenile timepoints as a binary trait and using the same covariates described in the GWASs outlined above. Loci that passed the *p*‐value cutoff of 1e‐2 here and also in the deep forereef bleaching survival GWAS were identified as “overlapping loci” and annotated genes were identified ±500 kb from the overlapping loci.

### 4.8 Role of Symbiodiniaceae in Bleaching Response

Pearson's correlation test (Freedman 2007) was used to examine the relationship between colony health score and proportion of symbiont genus (determined using mapped reads to Symbiodiniaceae genera genomes as described above; read counts available in File S1). Mann–Whitney *U* tests validated changes in proportion of each Symbiodiniaceae genus between the three timepoints. Reads were mapped to only three Symbiodiniaceae genera genomes because of their consistent presence in ITS2 data, with a noticeable lack of any detectable *Breviolum* symbiont types (Leinbach et al. 2023, Figure S27). GLMs were also performed with main effects of reef habitat, reads mapping to each symbiont genus, colony size, and LTER location to examine the interaction between reef habitat and symbiont genus on health score.

Juveniles collected in 2021 were also sequenced for ITS2 (*N* = 115, Table S1). ITS2 amplicons were generated via PCR using Symbiodiniaceae‐specific primers SYM_VAR_5.8S2 and SYM_VAR_REV (Hume et al. 2018). Libraries were sequenced on the Illumina MiSeq platform with 300‐bp paired‐end reads at the Georgia Genomics and Bioinformatics Core at the University of Georgia. Paired forward and reverse ITS2 sequences from juveniles combined with ITS2 sequences from post‐mortality timepoints (August 2019 [*N* = 16] and October 2019 [*N* = 58]; Leinbach et al. 2023) were submitted to SymPortal (Hume et al. 2019) to predict ITS2 type profiles (see Figure S37), which represent putatively different taxa.

## Conflicts of Interest

The authors declare no conflicts of interest.

## Supporting information


**Data S1:** eva70198‐sup‐0001‐DataS1.docx.

**Data S2:** eva70198‐sup‐0002‐DataS2.xlsx.

## Data Availability

All analysis pipelines are open source and can be found at https://github.com/jamesfifer/MooreaWGS. Raw read data is available on NCBI's sequence read archive (SRA) under PRJNA1250174.
