# Supplementary material for: Rapid Evolution in a Coral Population Following a Mass Mortality Event
Source: Evol Appl. 2026 Feb 1;19(2):e70198. doi: 10.1111/eva.70198 (PMC12862241; doi:10.1111/eva.70198)
Supplement: Supplementary file 1 — Data S1: eva70198‐sup‐0001‐DataS1.docx. [file EVA-19-e70198-s002.docx]

**Supplementary Information for Genomic signatures of coral adaptation and recovery following a mass mortality event**

**Supplementary Methods**

*Coral collections*

Samples were collected via SCUBA from *A. hyacinthus* colonies surrounding the island of Mo’orea as follows: 1) adults during the May 2019 bleaching event (pre-mortality; N = 172), 2) adults after bleaching and thermal stress had subsided in October 2019 (post-mortality; N = 103), and 3) juveniles in November 2021 (N = 115). Sampling in May was carried out to maximally capture the entire bleaching phenotypic landscape, with an effort to sample bleached and unbleached colonies at sites whenever possible. However, bleaching varied across sites, so it was not possible to sample equal numbers of corals across the bleaching spectrum. At the time of sampling in May, we did not observe the onset of mortality in any of the corals, despite many in a severely bleached state. Therefore, we strongly suspect we sampled prior to the onset of any mortality. Ten colonies were repeatedly sampled at both May 2019 and October 2019 timepoints.

At each LTER location, colonies were sampled from at least one of three reef habitats (backreef, shallow forereef, and deep forereef), corresponding to three different depths (1-3 m, 3-5 m, 10-14 m, respectively). While adults from May 2019 and October 2019 included sampling at all three reef habitats, juveniles from November 2021 were only sampled at the deep forereef (Supp. Table 1). Juveniles were characterized as small colonies < 8 cm long diameter, and care was taken to ensure they were not remnants of surviving adults. Most juveniles were observed and sampled on recently deceased *Pocillopora spp.* skeletons or coral rubble. Deceased *A. hyacinthus* skeletons are easily differentiated in this environment by their broad tabular morphology and any juvenile or live coral sample found on *A.* *hyacinthus* skeletons was not sampled to avoid potentially sampling surviving colony fragments*.* Photographs of sampled juveniles are available in data repository linked at https://github.com/jamesfifer/MooreaWGS. Each sample consisted of a 2-3 cm fragment, collected with bone cutters, immediately preserved in 200 proof ethanol, and stored at -80°C. For each colony sampled, photographs containing size and color standards were taken and colony area and diameters of short and long sides were calculated using ImageJ (Schneider et al., 2012). Health score was estimated visually by using a Coral Color Reference Card (Siebeck et al., 2006). Representative colonies for each health score are presented in Supplementary Figure 12. To better represent the impacts of thermal stress on bleaching severity, additional colonies (N = 226) were photographed only (*i.e.*, not sampled for sequencing) and assigned a health score (Supp. Table 1).

*Coral mortality and bleaching surveys*

Coral bleaching and mortality surveys were conducted at 10 m on the forereef of Mo’orea between July 9-15, 2019. Divers on SCUBA conducted two 50-m transects at each site, in which they quantified a binned bleaching and mortality measurement for all *A. hyacinthus* along a 1-m belt along the transect. Divers assessed bleaching and mortality for all *A. hyacinthus* greater than 5 cm in diameter. To avoid counting older mortality unrelated to the bleaching event, our “mortality” measure only included recently dead portions of the colony- *i.e.* if they had been colonized by turf algae but not yet colonized by macroalgae. For each colony, divers estimated the percentage of each colony that was bleached or recently dead (*i.e.* if a colony had 25% mortality and 25% bleaching of the colony area then that colony would have 50% bleaching and mortality). Divers used this binned measurement to allow for greater survey coverage during limited dive time and because each measurement separately underestimates the impact of the MHW.

*Temperature data*

Water temperature data were collected as part of the MCR LTER core time series data collection (Leichter et al., 2019). At four LTER locations (Fig. 1A), a bottom-mounted thermistor attached at 2 m (backreef) and 10 m (deep forereef) depth recorded water temperatures every 20 minutes. High wave energy prevents logger deployment at the shallow forereef. Cumulative heat stress was calculated from November 1, 2018 to December 1, 2021 as the 12-week running sum of weekly average temperatures exceeding 29°C, a noted bleaching threshold for corals in Mo’orea (Pratchett et al., 2013). The cumulative heat stress metric, as opposed to degree heating weeks (DHWs), is more sensitive and allows quantification of prolonged low level heating (*i.e.* does not require a full 1

$^{\circ}C$ above the maximum monthly mean to accumulate). This allows for higher resolution when comparing reef habitats that experienced the same MHW. Because LTERs 1, 2, and 3 did not record thermistor data for the entire desired timeframe, LTER 0 was substituted for temperature data at LTERs 1 and 2, and LTER 4 was substituted for LTER 3. As LTER 0 is only a few hundred meters away from LTER 1 and our sampling location fell between the two thermistor locations, we use the label “LTER 1” to refer to temperature data that came from the thermistor at LTER 0 in an effort to simplify figures. Correlations during time intervals with overlapping available data between sites suggest temperatures at substituted sites were extremely similar (Supp. Fig. 35). To address the concern that similarities between thermistor readings may decrease during heat stress events, we used available data from the subsequent 2020 heat stress event to show that cumulative heat stress was not different between substituted sites (Supp. Fig. 36).

*Whole Genome Sequencing (WGS)*

Genotyping and identification of SNPs were performed using ANGSD (Korneliussen et al., 2014). Standard filtering that was used across all analyses unless otherwise specified included loci present in at least 80% of individuals, minimum mapping quality score of 20, minimum quality score of 25, strand bias *p*-value > 0.05, heterozygosity bias > 0.05, minor allele frequency (MAF) > 0.05, a SNP *p*-value of 1×10^-5^, removing all triallelic loci, removing reads having multiple best hits and lumped paralogs filter. Samples that had more than 10% missing data were removed from downstream analyses (Supplemental File 1). A set of unlinked loci were identified using ngsLD (Fox et al., 2019), with LD pruning parameters genetic distance <= 50000 and r^2^ >= 0.2, this set of SNPs were used for population structure analyses (*i.e.* PCoAs and NGSadmix) and N_e_ estimates. For the number of SNPs and samples used for each analysis see Supplemental File 1.

*Clone identification and genetic structure between sites, habitats, and timepoints*

Clones were detected using hierarchical clustering of samples based on pairwise identity by state (IBS) distances calculated in ANGSD. Technical replicates were used to identify appropriate height cutoffs for clone identification revealing three pairs of clones total across all timepoints (Supp. Fig. 38). We also identified putative half siblings with (Korneliussen & Moltke, 2015) and randomly removed one individual from each pair for population structure analyses. To compare population structure between sites, habitats, and timepoints, our data were subset into the following datasets: 1) pre-mortality only (May 2019 samples), 2) post-mortality only (October 2019 samples), 3) juveniles only (November 2021 samples), 4) pre-mortality (May 2019) and post-mortality (October 2019) across only the common sites between the two timepoints (LTER 1 backreef and deep forereef, LTER 2 shallow forereef and deep forereef, LTER 3 deep forereef, and LTER 5 deep forereef), and 5) all three timepoints across the sites common between all timepoints (LTER 1 deep forereef, LTER 2 deep forereef, LTER 3 deep forereef, and LTER 5 deep forereef). For each of the five datasets, genetic structuring between sites (datasets 1-3) or timepoints (datasets 4 and 5) was compared using PCoA via a covariance matrix based on single-read resampling calculated in ANGSD. DAPC was also performed from the PCoA table. DAPCs were carried out using the *lda* function from the R (R Core Team, 2023) package MASS (Ripley et al., 2013), where the number of PCs to retain was determined using scree plots and CV (checking 1: N/3 PCs, where N is the total number of eigenvalues). Additionally, DAPCs were used to identify potential source populations for juveniles. To do so, DAPCs were performed using adult colonies from 2019 (both pre- and post-mortality) only as training data. DAPCs were conditioned on site (all sites, irrespective of year, for which there were adult colonies; Supp. Table 1) and the resulting model was used to predict juvenile site assignment. Population structure was also assessed using NGSadmix (Skotte et al., 2013) and optimal K using the Evanno method (Evanno et al., 2005). PCoAs incorporating WGS data from Rose et al. (2021) were used to confirm that all samples were *A. hyacinthus*, genotypes were called using the same set of ANGSD filters as those used for the Moorea samples (described in section *Whole Genome Sequencing (WGS)* above).

Expected heterozygosity was calculated from each individual’s site frequency spectrum (SFS) (dividing singletons by all loci) generated by first calculating the site allele frequency (SAF) in ANGSD with no MAF filter and then the unfolded (using the *A. hyacinthus* reference genome (López-Nandam et al., 2023) as an ancestral reference) SFS in winsfs (Rasmussen et al., 2022). Differences in individual heterozygosities between sites and timepoints were calculated via Dunn’s test (1964) with a Benjamini-Hochberg multiple test correction. As this only assesses the increase or decrease of heterozygous individuals, we also looked at genetic diversity dynamics of the population by calculating nucleotide diversity (Watterson’s θ) for each site at each timepoint using ANGSD’s *thetastat* function on the SFS for each population (randomly downsized to the minimum number of individuals for each site across timepoints). A similar method was also used to calculate π, using the pairwise θ from ANGSD’s *thetastat* function as described in Adams et al., (2023). To examine genetic differentiation between sites, SFSs were used as priors with the SAF to calculate genome-wide *F*_ST_. Here, only weighted genome-wide *F*_ST_ values between populations are reported. To determine how large *A. hyacinthus* N_e_ are compared to other *A. hyacinthus* populations, StairwayPlot V2 (Liu & Fu, 2020) was used to model N_e_ through time. Unfolded SFSs for N_e_ estimates were generated by first identifying a set of putatively unlinked loci through linkage disequilibrium (LD) pruning in ngsLD and using a per generation mutation rate of 2*e*-8 and a generation time of 5 (Fifer et al., 2022).

*Genome-wide association study (GWAS) on variation in bleaching resistance in pre-mortality samples and bleaching survival in pre- and post-mortality samples*

A GWAS for bleaching resistance was performed for pre-mortality samples taken during the bleaching event (N = 172), with health score as the trait, serving as a proxy for bleaching resistance (Fig. 1C). General linear models (GLMs) were used to test for additive effects of SNPs (with minor allele frequencies > 5%) on the quantile-normalized health score (Zhou et al., 2017; Zhou & Stephens, 2012), including as covariates the first two genetic PCos, non-genetic/environmental variables (surface area of the colony and the collection depth), and the proportion of *Symbiodinium* and *Cladocopium* reads (as two separate terms) relative to all symbiont reads. Reads mapping to three Symbiodiniaceae genera genomes (*i.e.*, *Symbiodinium, Cladocopium,* and *Durusdinium*) were used as an approximation of the proportion of symbionts in each sample following Fuller et al. (2020). We only included *Symbiodinium* and *Cladocopium* proportions terms in the model as *Durusdinium* is still accounted for as 1 – *Symbiodinium* and *Cladocopium* proportions and inclusion of *Durusdinium* would result in perfect collinearity. Under a scenario of low to no population structure it might be unnecessary to include the whole-genome PCo1 and PCo2 as covariates, to test whether the addition of these covariates was significantly altering results GWAS p-value and beta-values were compared between a model including versus excluding the PCos with a Pearson correlation test. Additional filtering included removing loci with more than 10% individuals missing and filtering out SNPs with Hardy-Weinberg *p*-values below 1*e*-7 and because PLINK2 uses hardcalls instead of genotype likelihoods, genotypes were only called if the posterior was above 0.95. For the GLM, a GWAS was performed using a standard linear regression as implemented in PLINK2. Genome-wide cutoffs were determined through 10,000 permutations of the GLM, randomly shuffling the trait values, extracting the minimum *p*-value for each run, and then taking the value of the 95^th^ percentile of this distribution (Fuller et al., 2020).

A GWAS was also carried out assigning the pre- and post-mortality timepoints as a binary trait (*i.e.*, bleaching survival) to calculate a GWAS for bleaching survival. An alternative design here would have been to follow samples and score whether they survived or not, however it is not necessary to follow the same samples to detect selection in a population following a mortality event (*e.g.*, Schiebelhut et al., 2018). Additionally, following a set of samples is not necessarily less biased, as it would be biased towards that set of individuals instead of the larger population. Here we use a logistical regression model in PLINK to accommodate the binary trait and repeat all covariates listed above. For the bleaching survival GWAS we also omitted colonies from the pre-mortality timepoint that were also present at the post-mortality timepoint (10 colonies). We scored these colonies only as post-mortality given all colonies from the post-mortality timepoint were also technically present at the pre-mortality timepoint and marking these colonies in both timepoints would not accurately score them as survivors. We also exclude all LTER 2 colonies from the post-mortality timepoint as complete mortality at the original site at 10 m necessitated sampling from 5 m deeper (*i.e.* at 15 m).

We also repeated both the bleaching resistance and bleaching survival GWAS subsetting the data into deep forereef only to investigate reef habitat specific effects. Information on which samples were included in each GWAS is available in Supplemental File 1.

*Calculation of bleaching resistance and bleaching survival PGSs*

Given that bleaching resistance and survival are likely influenced by a combination of genetic and non-genetic factors (Fuller et al., 2020) we do not necessarily expect the PGS to increase predictability on its own. Instead, it is more meaningful to assess whether a model with important non-genetic predictors of bleaching is improved when adding the PGS. We do so by assessing the change in R^2^ using Mann-Whitney U tests between linear models with different combinations of predictors (1. Genetic PCos, 2. Genetic PCos + Non-genetic/Environmental variables, 3. Genetic PCos + Non-genetic/Environmental variables + PGS, 4. Genetic PCos + Non-genetic/Environmental variables + Symbiont proportions, 5. Genetic PCos + Non-genetic/Environmental variables + Symbiont proportions + PGS). Specifically, comparisons were made between the predictability of model 5 versus model 4 and model 3 versus model 2, with the expectation that the PGS would increase the predictability of health score and survival. In instances where model 5 or 3 increased predictability relative to model 4 or 2, we also compared whether the respective model increased predictability when replacing the real PGS with a null PGS generated from randomly selecting the same number of loci and recalculating the PGS using the exact same method (including LD clumping) as the real PGS. Lastly, because we are not able to impute genotypes at missing data (due to the lack of a high coverage reference panel) it is possible that if the 10% missing data is distributed non randomly it could artificially inflate the predictability of the PGS. To address this possibility for the significantly predictive PGSs, we also tested the predictability of a PGS created from loci with no missing data.

*Role of Symbiodiniaceae in bleaching response*

To identify if host genetic variation structures symbiont communities, we performed an RDA from the symbiont dominance information gathered from WGS reads. RDA models were conducted with individual-based genotypes, where we imputed the most common genotype at each SNP across all individuals for missing data. A host genotype matrix (012 allele coding system) was created and designated as the response variable whereas symbiont dominance (*i.e.*, which symbiont had the highest proportion of reads) was designated as the explanatory variable; then significance was examined with an ANOVA. We also performed the reverse, where the proportion of symbiont genera was designated as the response variable and then either reef habitat or host genetic variation were explanatory variables. Because of the RDAs limitations in handling multidimensionality (*i.e.* millions of SNPs) for explanatory variables, for the reversed RDA we transformed the host data from SNP matrices to PCos that cumulatively explain 80% of the host genetic data variation. After combining the 2019 and 2021 juvenile ITS2 data, a total of 78 ITS2 type profiles were produced from SymPortal. ITS2 type profile reads were normalized using the trimmed mean of M-values (TMM) method, using function *calcNormFactors* in the edgeR package (Anders et al., 2010) to adjust for differences in sequencing depth. The 78 ITS2 type profiles were collapsed into nine distinct profiles based on PCoAs of their Bray-Curtis indices (Supp. Fig. 37), then used in subsequent analyses. One profile (F3x) was excluded from analyses due to low read counts in a single sample (N2F57J). To explore how timepoint impacted symbiont community structure at the site for which there was data at both timepoints (LTER 2 deep forereef; six distinct ITS2 profiles), a NMDS plot was generated. Bray-Curtis dissimilarities were calculated from a matrix of ITS2 profile abundance for each sample. Multivariate dispersion was measured using the function *betadisper* on Bray-Curtis dissimilarities and permutational analysis of variances (PERMANOVAs), using the function *adonis* in the vegan package (Okansen et al., 2020), to assess changes in symbiont community structure between post-mortality and juvenile timepoints. We employed a linear mixed-effects model (LME) to analyze the relationship between ITS2 type profile richness and colony size and a linear model (LM) to determine if colony size differed by site.

**Supplementary Results**

To ensure that the predictive ability of the deep forereef PGSs was not driven by missing data artifacts, we repeated this analysis with a subset of individuals and loci that contained no missing data to determine whether missing data was distributed non-randomly and inflating predictability of the PGS. A low sample number for the deep forereef bleaching resistance PGS training set for this particular no-missing-data subset (N=40) limited our assessment of missing data to the deep forereef bleaching survival PGS only, however, we do find the predictability of the deep forereef bleaching survival PGS is maintained even when calculating with the no-missing-data subsets (Supp. Fig. 24). This emphasizes the predictive power of the deep forereef PGS and highlights this power is not limited by missing data.

While the strong correlation between having a mixed symbiont community and showing a bleached phenotype (Fig. 5A), suggests the predictive power of symbiont genera here is heavily inflated and thus we would not necessarily expect the PGS to add predictive power for this comparison, we were still interested in comparing the PGS with the predictive power gained from inclusion of symbiont communities to follow methods from Fuller et al (2020). We found it that it increases the predictability for the deep forereef bleaching resistance PGS but not for the other PGSs (Supp. Fig. 25; Supp. Fig. 26). Given the aforementioned confounding effect of the symbiont community with the phenotype we are trying to predict, we caution against over-interpreting this particular analysis. That said, our findings on bleaching resistance are consistent with Fuller et al. (2020). However, this type of analysis appears less informative for predicting survival, emphasizing the importance of collecting pre-bleaching symbiont data for models that incorporate symbiont composition.

**
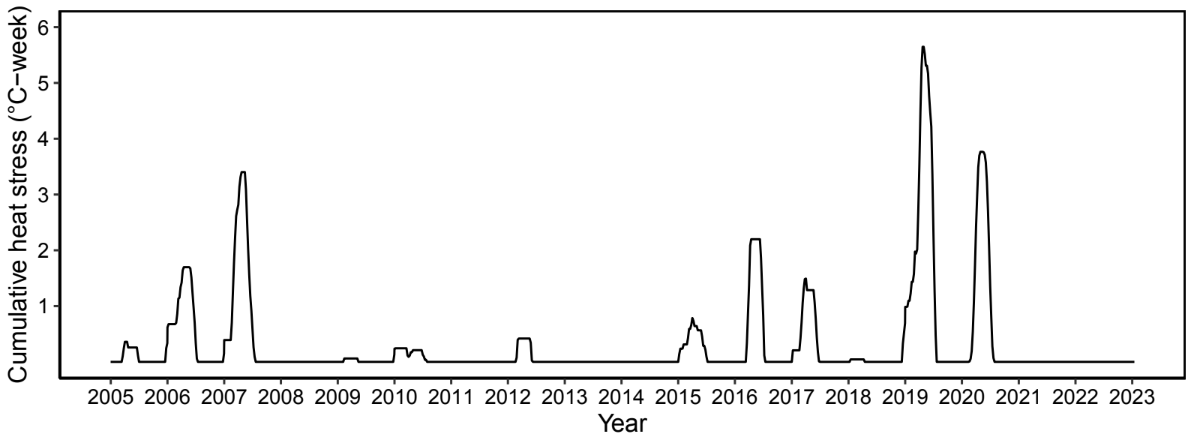
Supplementary Figures and Tables**

Supplementary Figure 1. Cumulative heat stress experienced across all MCR LTER sites (0-6) at 10 m depth on the forereef of Mo’orea over the prior 18 years. Cumulative heat stress was calculated as the 12-week running sum of all man 29°C. Data were collected as part of the MCR LTER core time series data collection (Edmunds & Moorea Coral Reef LTER, 2022).


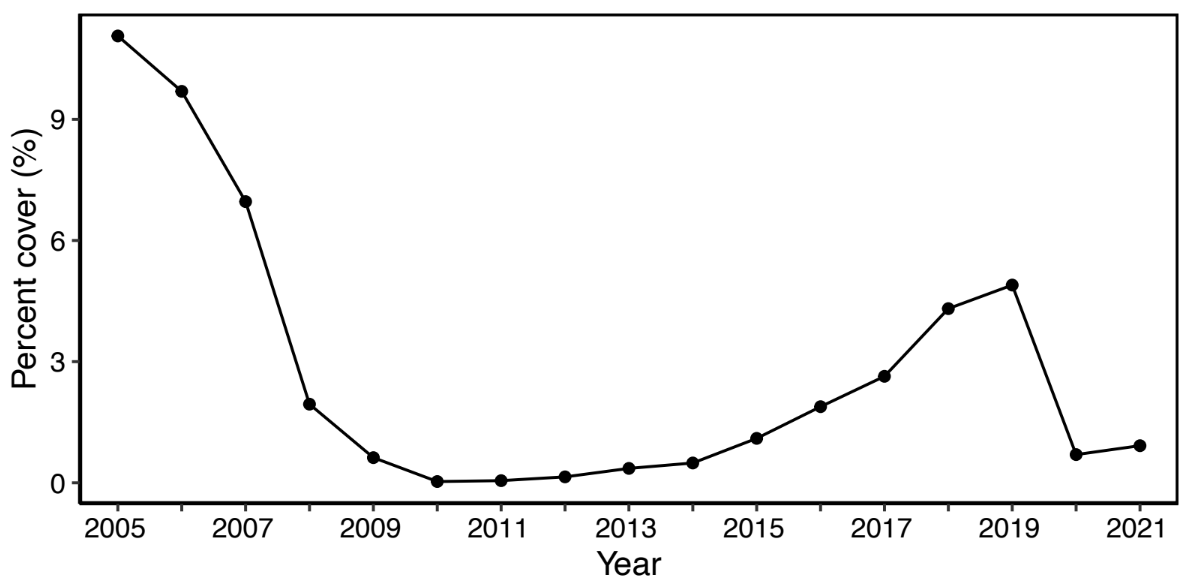
Supplementary Figure 2. *Acropora* spp. percent cover on the deep forereef (10 m depth) of Mo’orea, French Polynesia. Coverage surveys were conducted at MCR LTER sites 1-6 at 10 m water depth in April of each year. Each point represents the average across all sites per year. Data were collected as part of the MCR LTER core time series data collection (Edmunds & Moorea Coral Reef LTER, 2022).


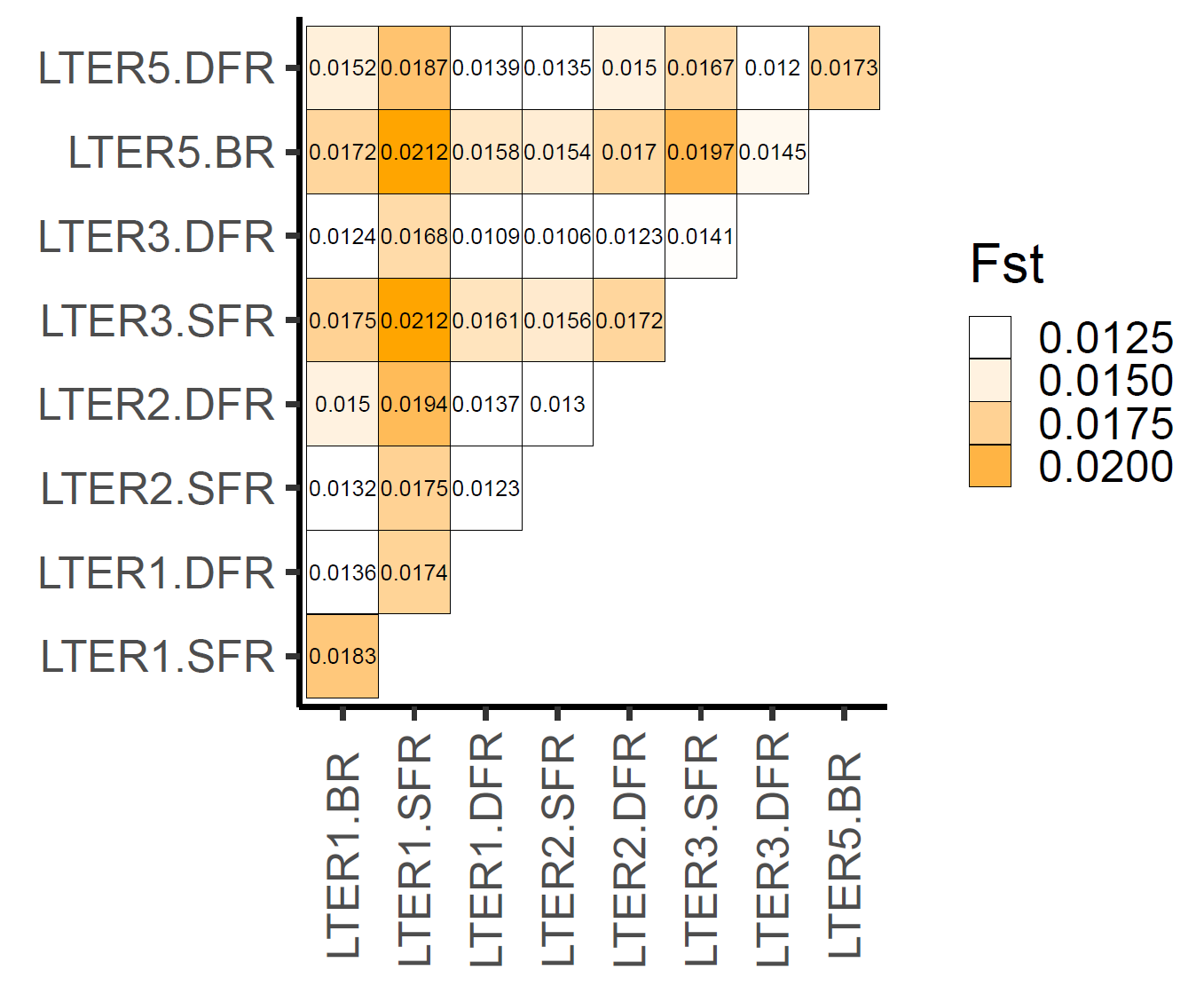
Supplemental Figure 3. Pre-mortality comparisons of pairwise *F*_ST_ between all sites, showing limited population structure. BR: Backreef; SFR: Shallow forereef; DFR: Deep forereef.


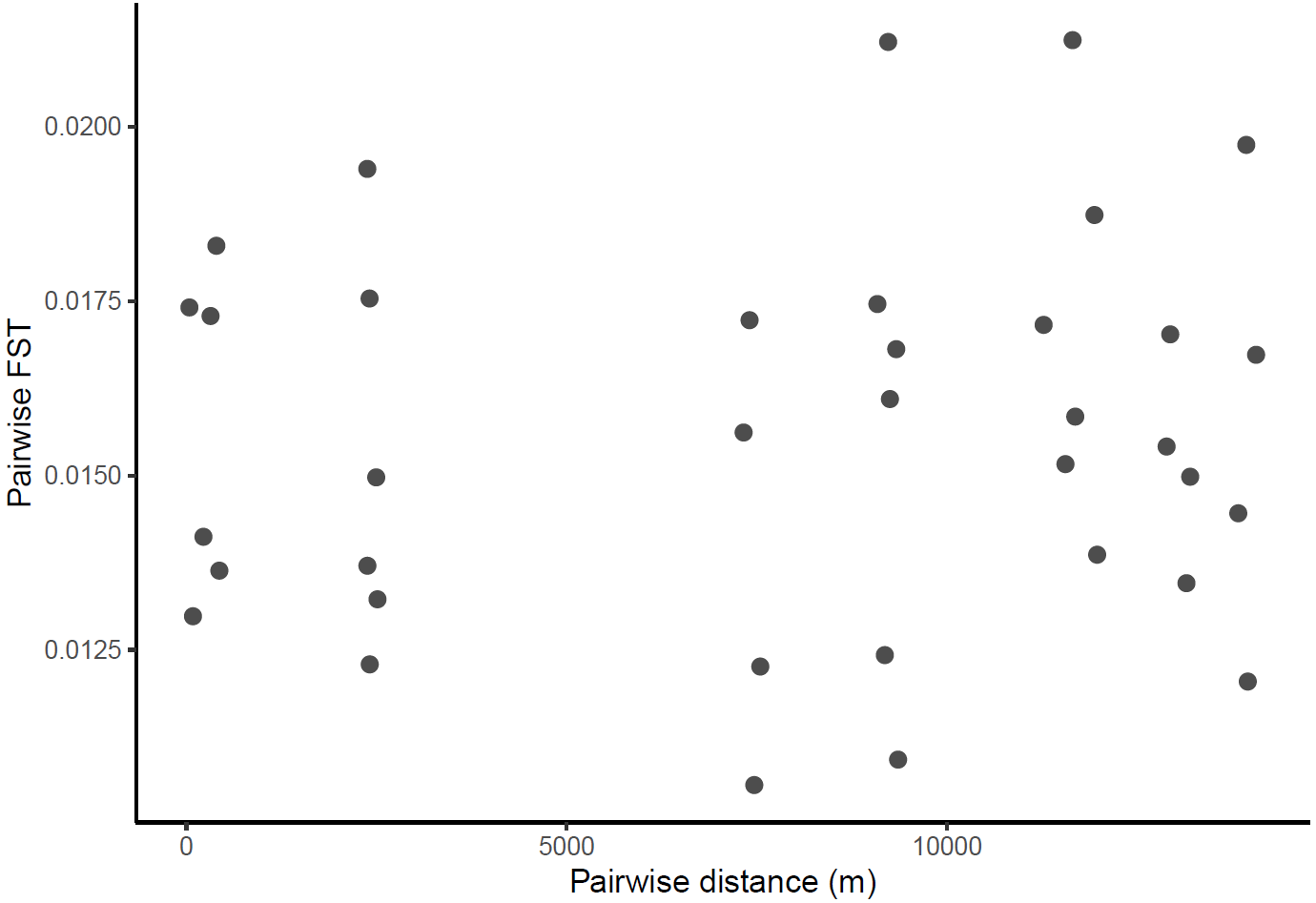


Supplemental Figure 4. Pre-mortality comparisons of pairwise *F*_ST_ between all sites, showing no signature of isolation by distance. Pairwise distance is based on euclidean distance calculated from Universal Transverse Mercator (UTM) coordinates.


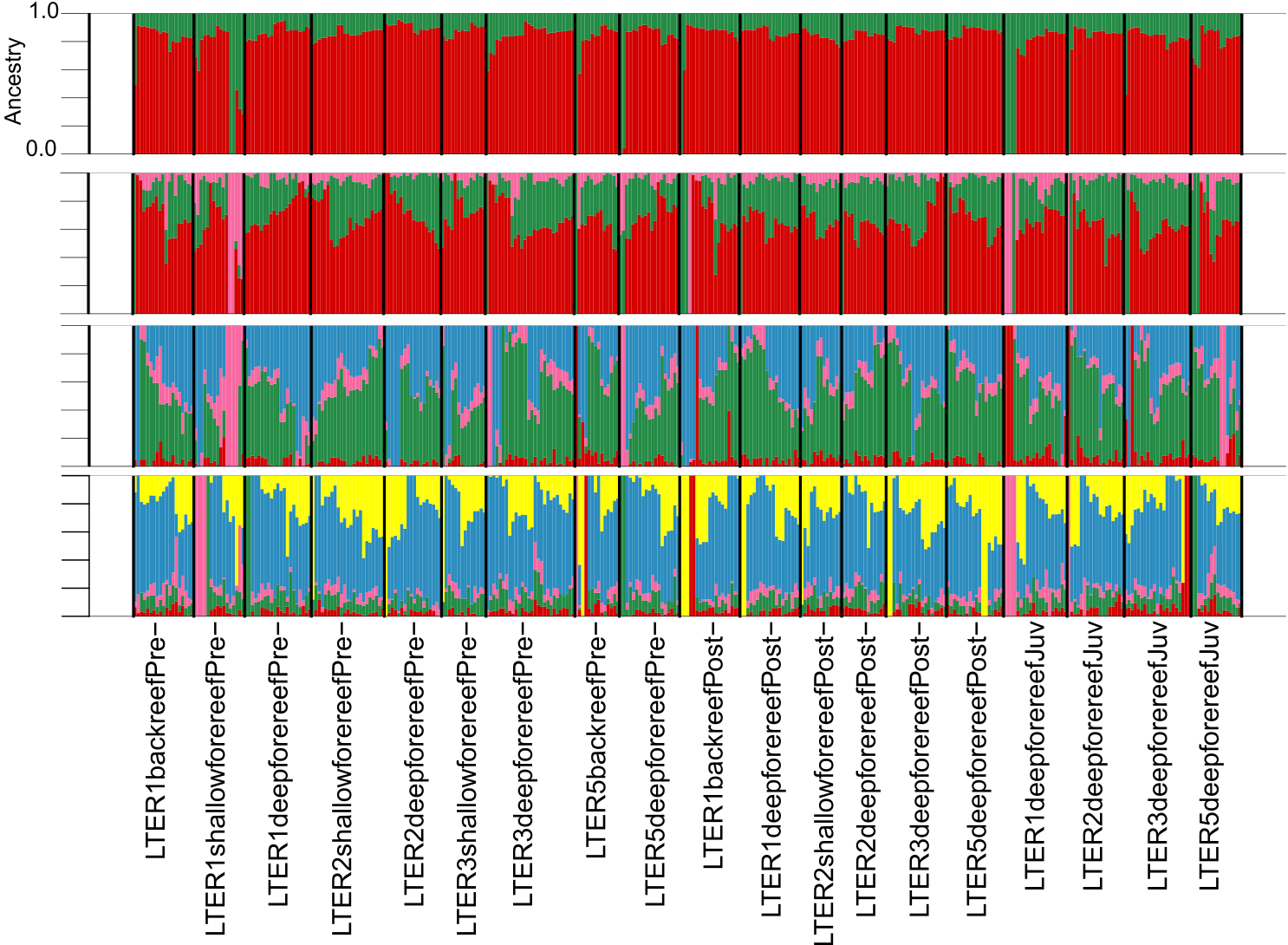
Supplemental Figure 5. Admixture plot showing all loci, Ks 2:5.


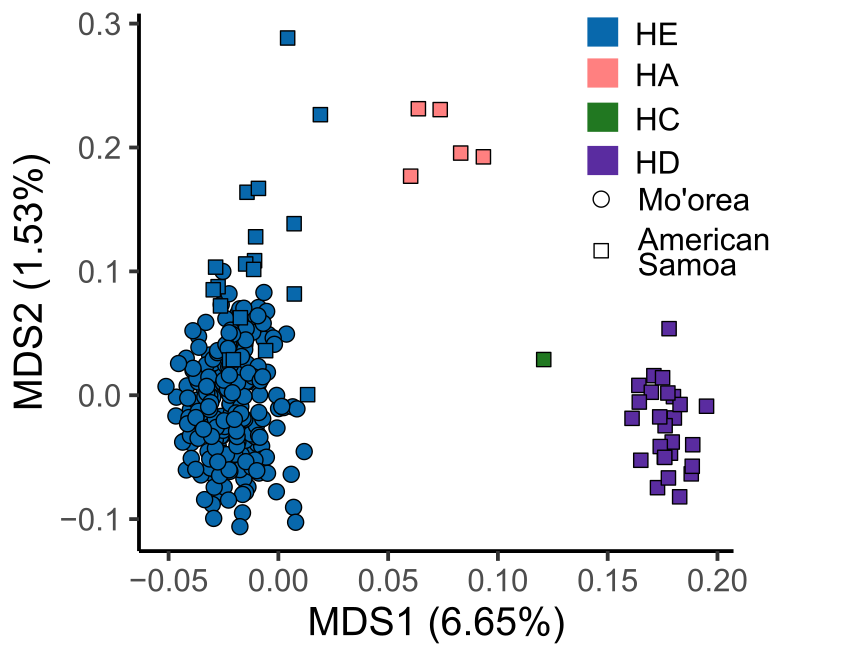
Supplemental Figure 6. MDS plot with *A. hyacinthus* WGS data from Rose et al. (2021), showing Mo’orea (including all adult and juvenile samples) clustering with HE lineage on
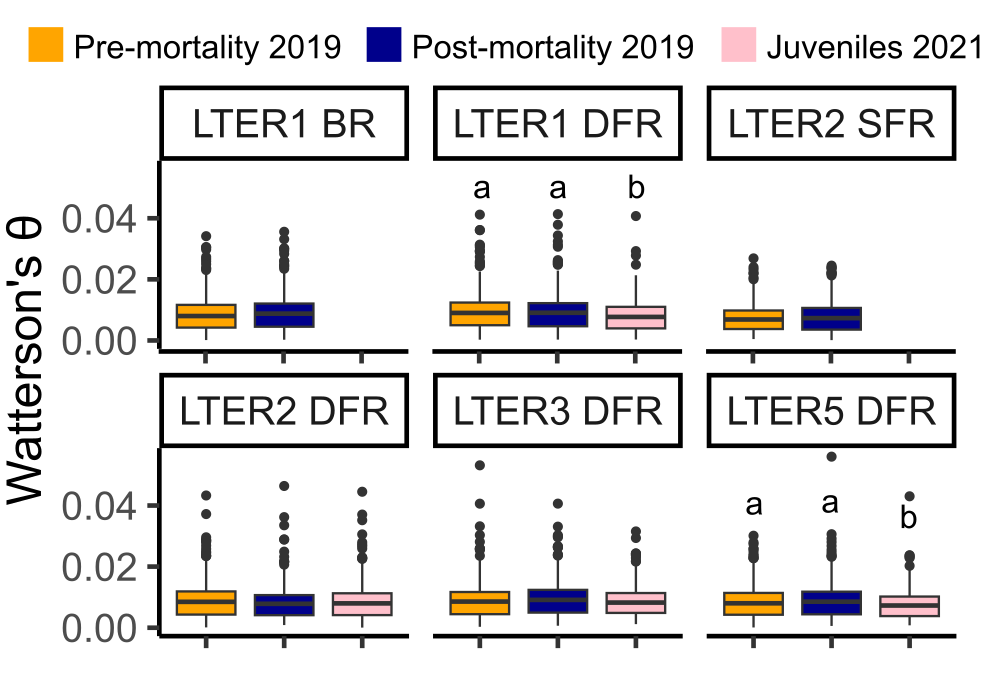
MDS1.

Supplemental Figure 7. Comparisons of nucleotide diversity (Watterson’s θ) between the overlapping sites for the three timepoints (pre- and post- mortality and juveniles). Letter denotes significance per Dunn’s test (1964) with a Benjamini-Hochberg multiple test correction (p < 0.001). BR: Backreef; SFR: Shallow forereef; DFR: Deep forereef.


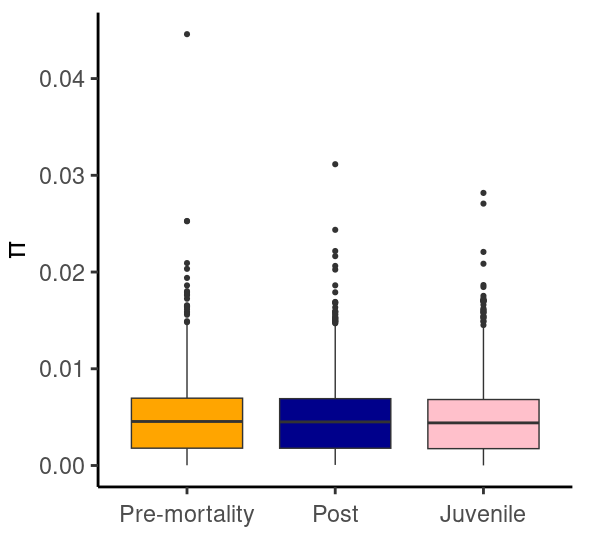


Supplemental Figure 8. π (pairwise theta in ANGSD) across scaffolds compared across the three timepoints (overlapping sites only). No letters denotes lack of significance per Dunn’s test (1964) with a Benjamini-Hochberg multiple test correction (p <0.001).


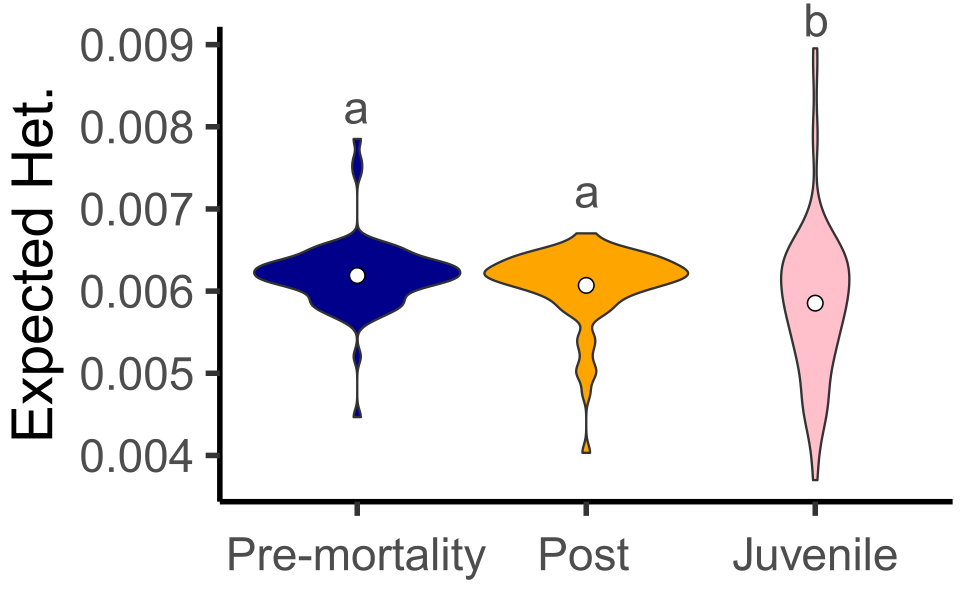


Supplemental Figure 9. Individual expected heterozygosity compared across the three timepoints (overlapping sites only). Letter denotes significance per Dunn’s test (1964) with a Benjamini-Hochberg multiple test correction (p <0.001).


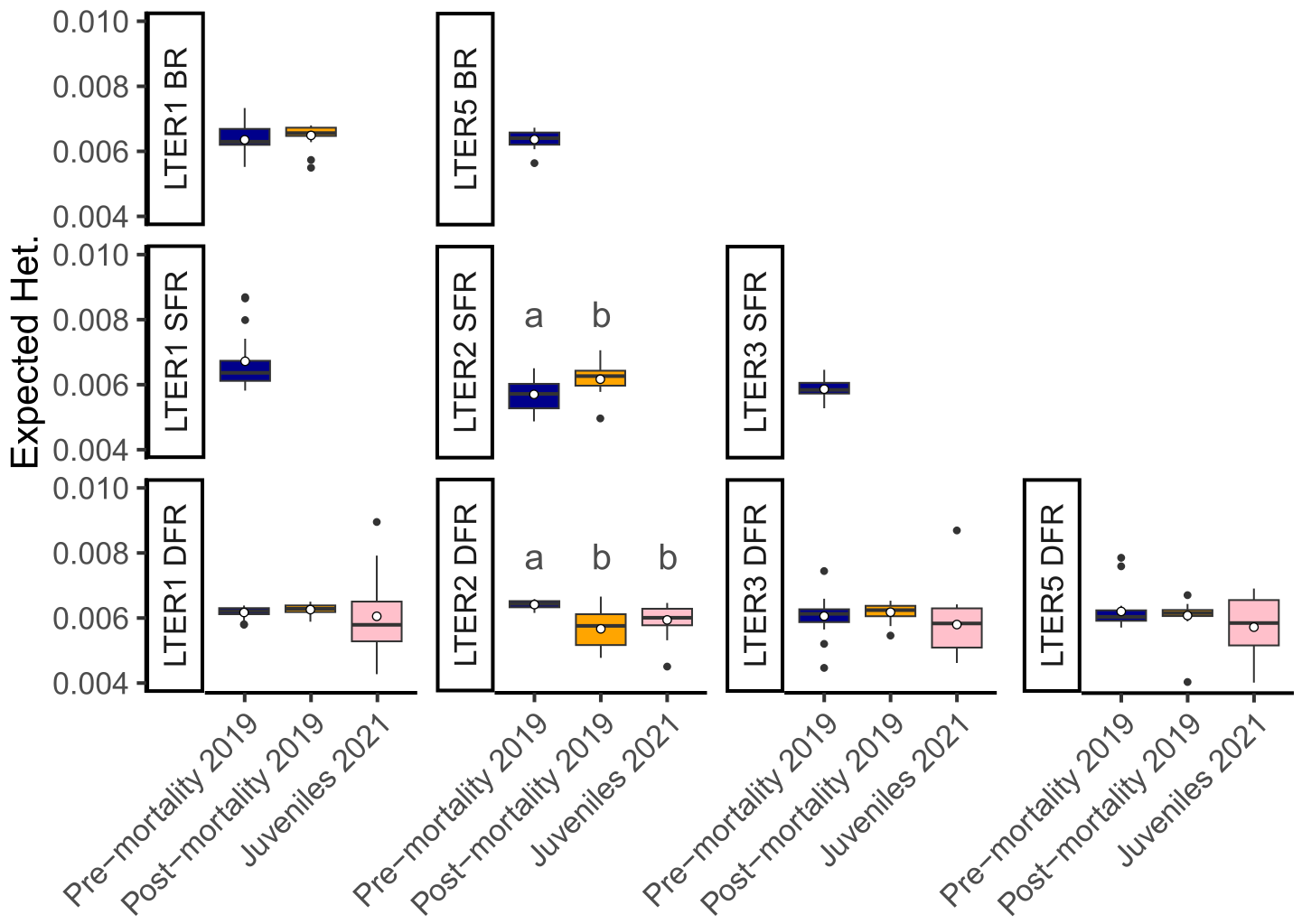


Supplemental Figure 10. Individual expected heterozygosity between all timepoints and site combinations sampled. Letters denote significance within each site per Dunn’s test (1964) with a Benjamini-Hochberg multiple test correction (p <0.001). Each facet that contains no letters had no significant differences.  BR: Backreef; SFR: Shallow forereef; DFR: Deep forereef.


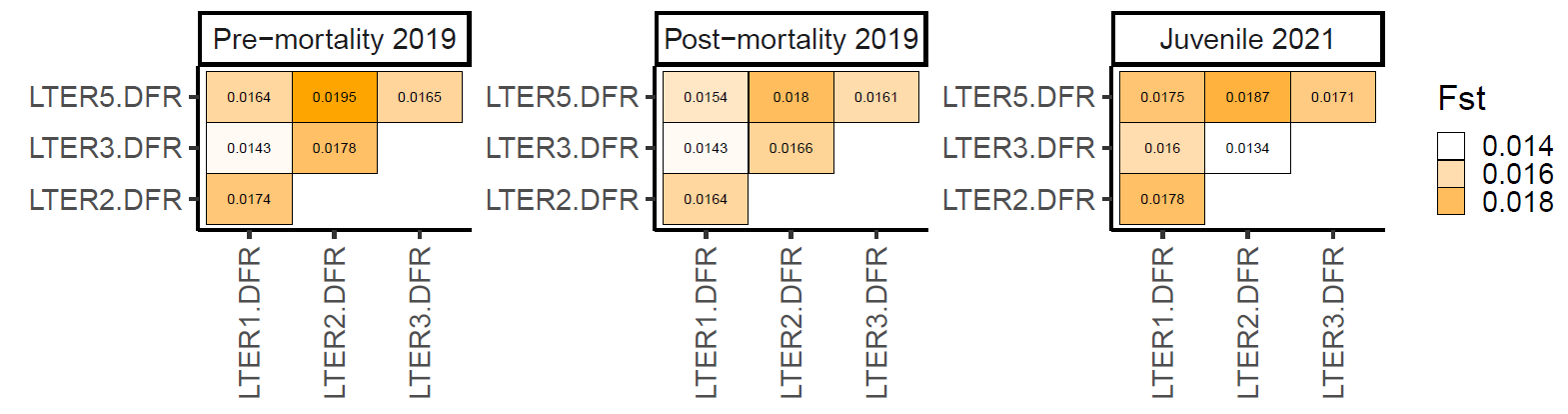


Supplemental Figure 11. Pairwise *F*_ST_ between deep forereef sites (DFR) for each timepoint.


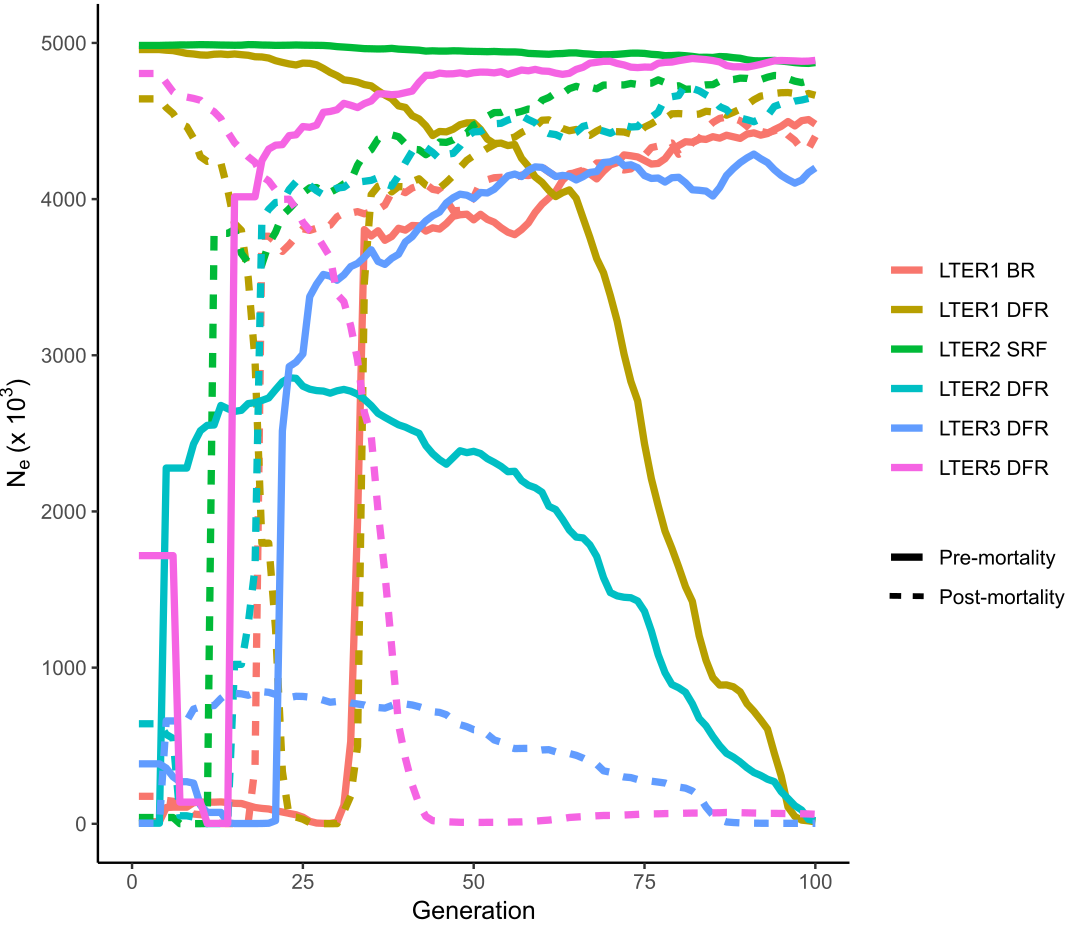


Supplemental Figure 12. GONE effective population size (Ne) estimates showing large estimates (*i.e.* > 1,000,000) for several Mo’orea *A. hyacinthus* populations. BR: Backreef; SFR: Shallow forereef; DFR: Deep forereef.


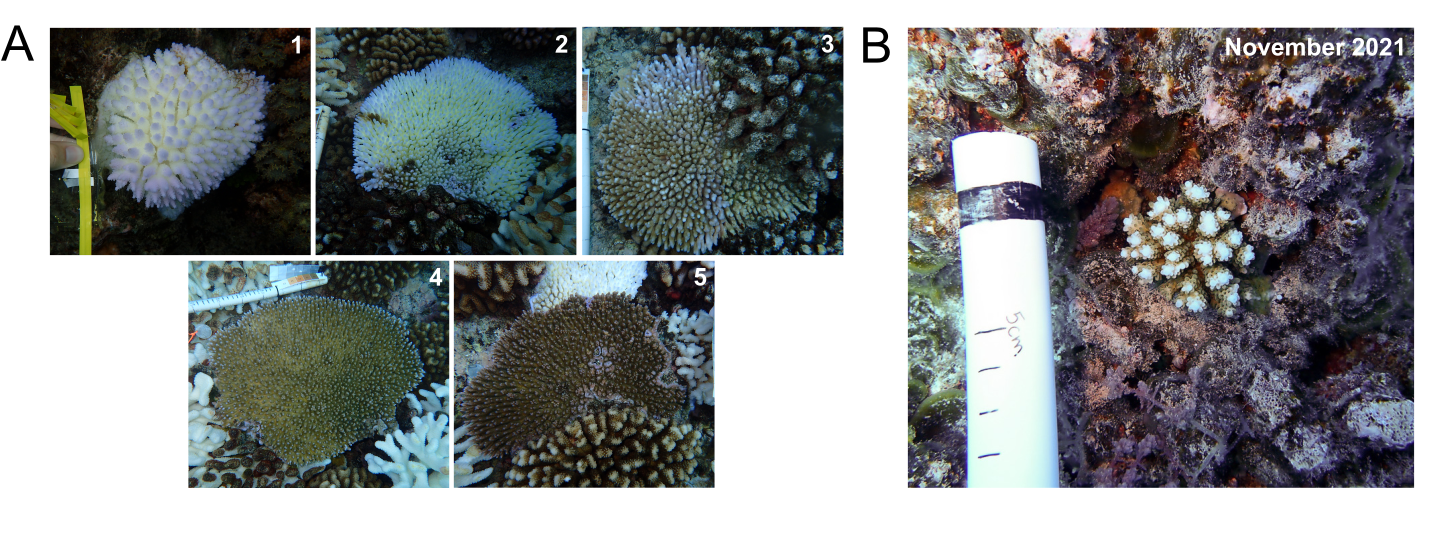

Supplemental Figure 13. Observed *A. hyacinthus* colonies during and after the 2019 thermal anomaly. A) Representative colonies illustrating the scale used to categorize bleaching intensity. Each colony was assigned an integer score from 1 to 5, with 1 indicating stark white bleaching and 5 indicating dark pigmentation and no bleaching. B) A juvenile *A. hyacinthus* colony settled after the 2019 MME, photographed in November 2021. Figure adapted from Leinbach et al. (2023).
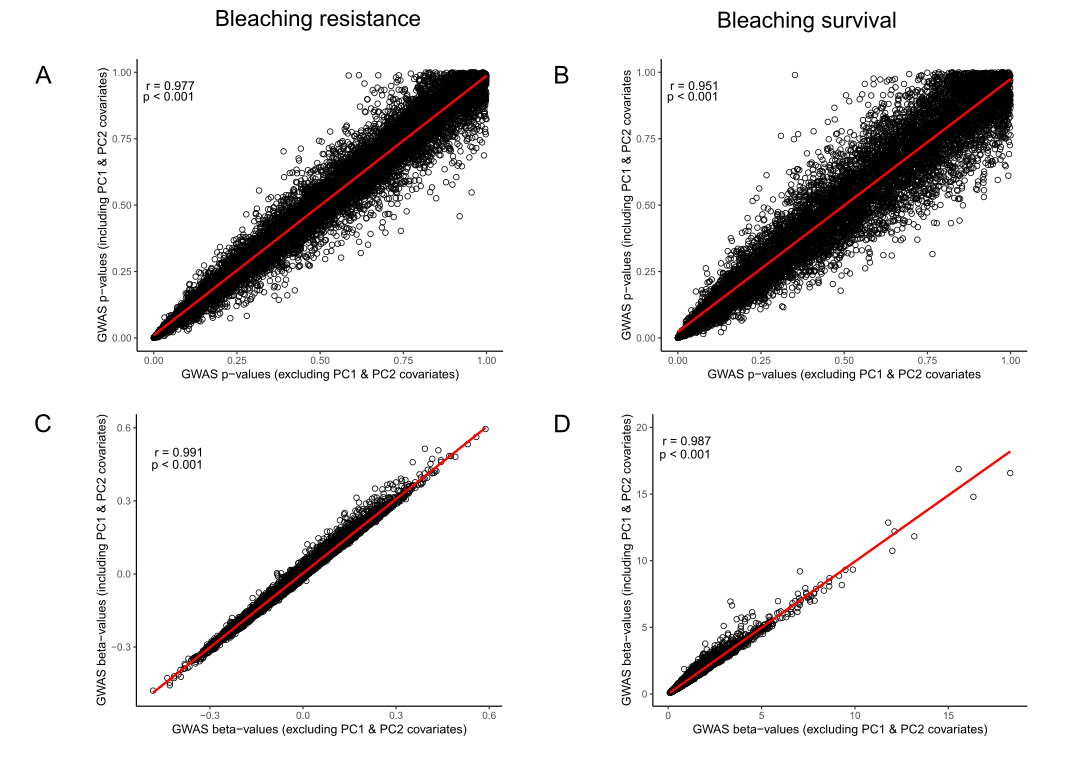


Supplementary Figure 14. Correlation between GWAS summary statistics A,B) p-values and C,D) beta values, for bleaching resistance and bleaching survival GWASs including or excluding PCo1 and PCo2 (from a PCoA performed on the genetic covariance with the relevant subset of samples) as covariates. Correlation coefficient is reported for all loci, randomly selected 50,000 loci are used for plotting purposes.


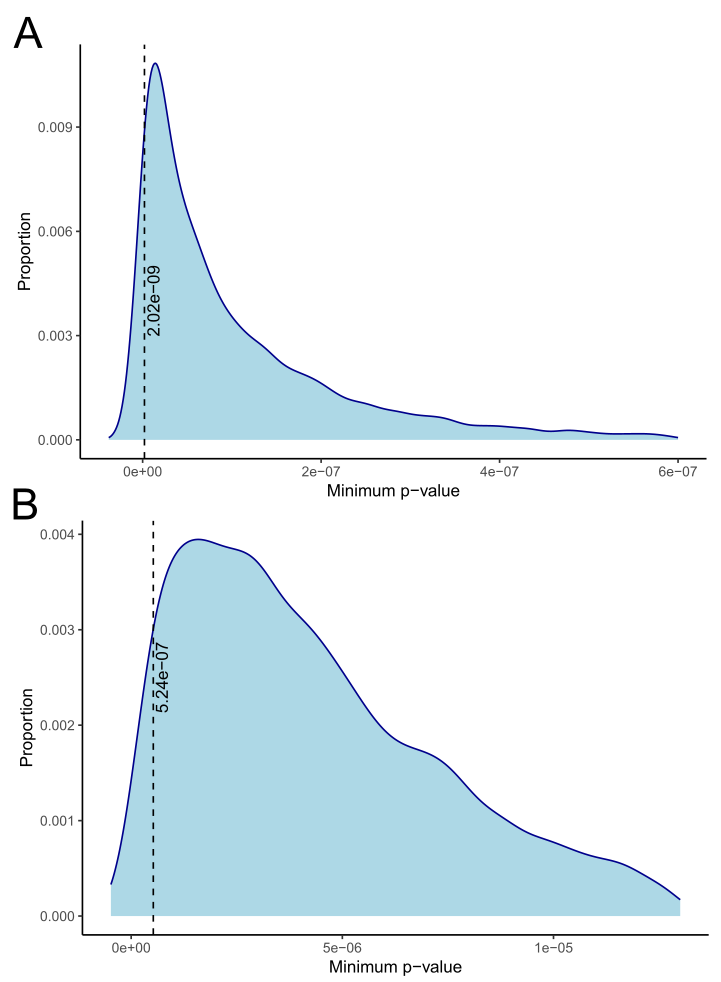

Supplemental Figure 15. Distribution of minimal *p*-values determined from a permutation test to estimate a threshold of genome-wide significance for bleaching resilience (A) and bleaching survival (B). The 95^th^ percentile for each is indicated with a vertical line and was used as the genome-wide significance threshold.


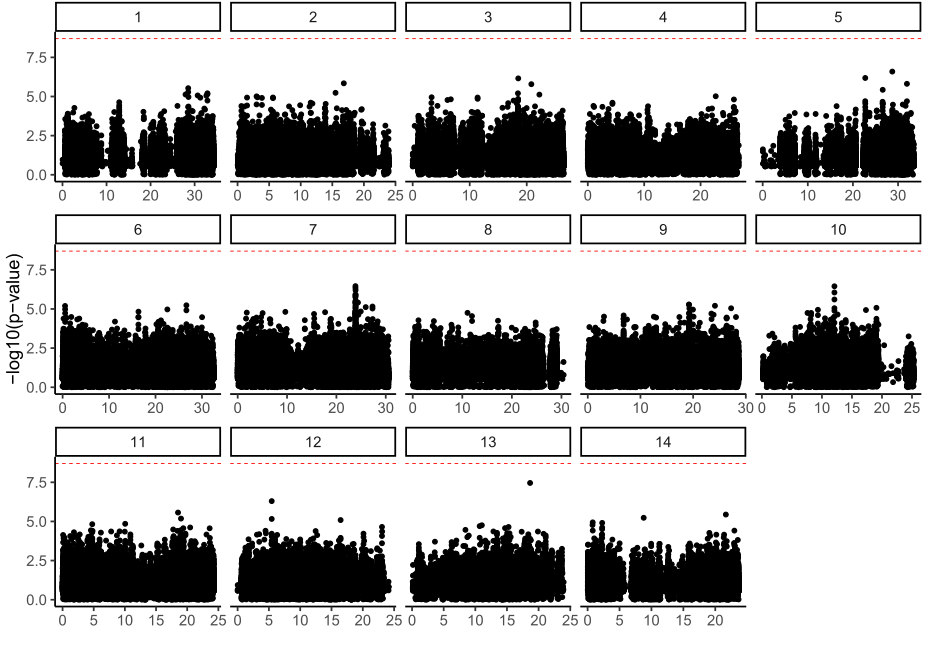

Supplemental Figure 16. Manhattan plot subsetted by chromosomes for bleaching resistance GWAS shows no significant loci. Dotted horizontal red line shows genome-wide cutoffs determined through 10,000 permutations of the GLM, randomly shuffling the trait values, extracting the minimum *p*-value for each run, and then taking the value of the 95^th^ percentile of this distribution (Fuller et al., 2020).


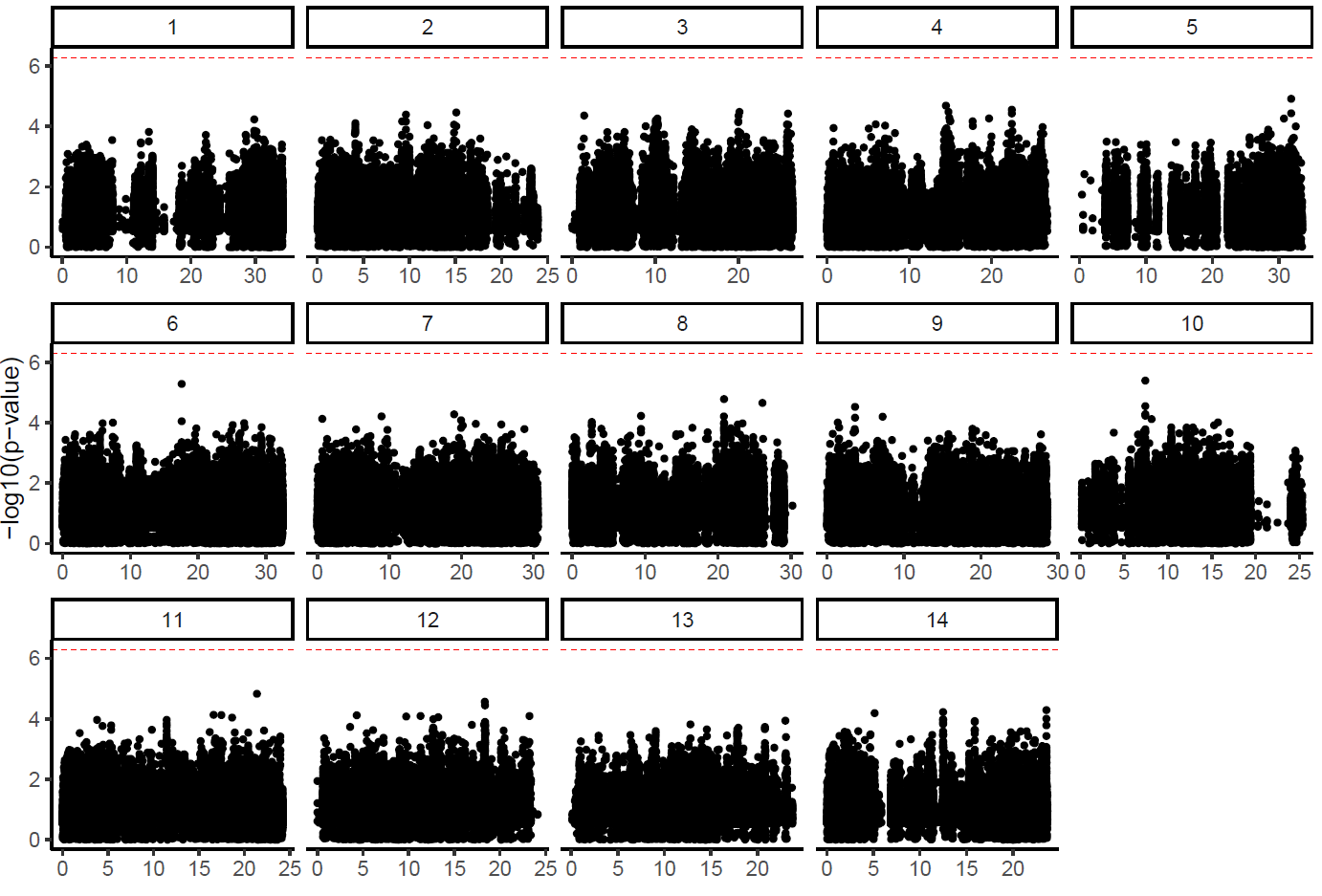


Supplemental Figure 17. Manhattan plot subsetted by chromosomes for bleaching survival GWAS shows no significant loci. Dotted horizontal red line shows genome-wide cutoffs determined through 10,000 permutations of the GLM, randomly shuffling the trait values, extracting the minimum *p*-value for each run, and then taking the value of the 95^th^ percentile of this distribution (Fuller et al., 2020).

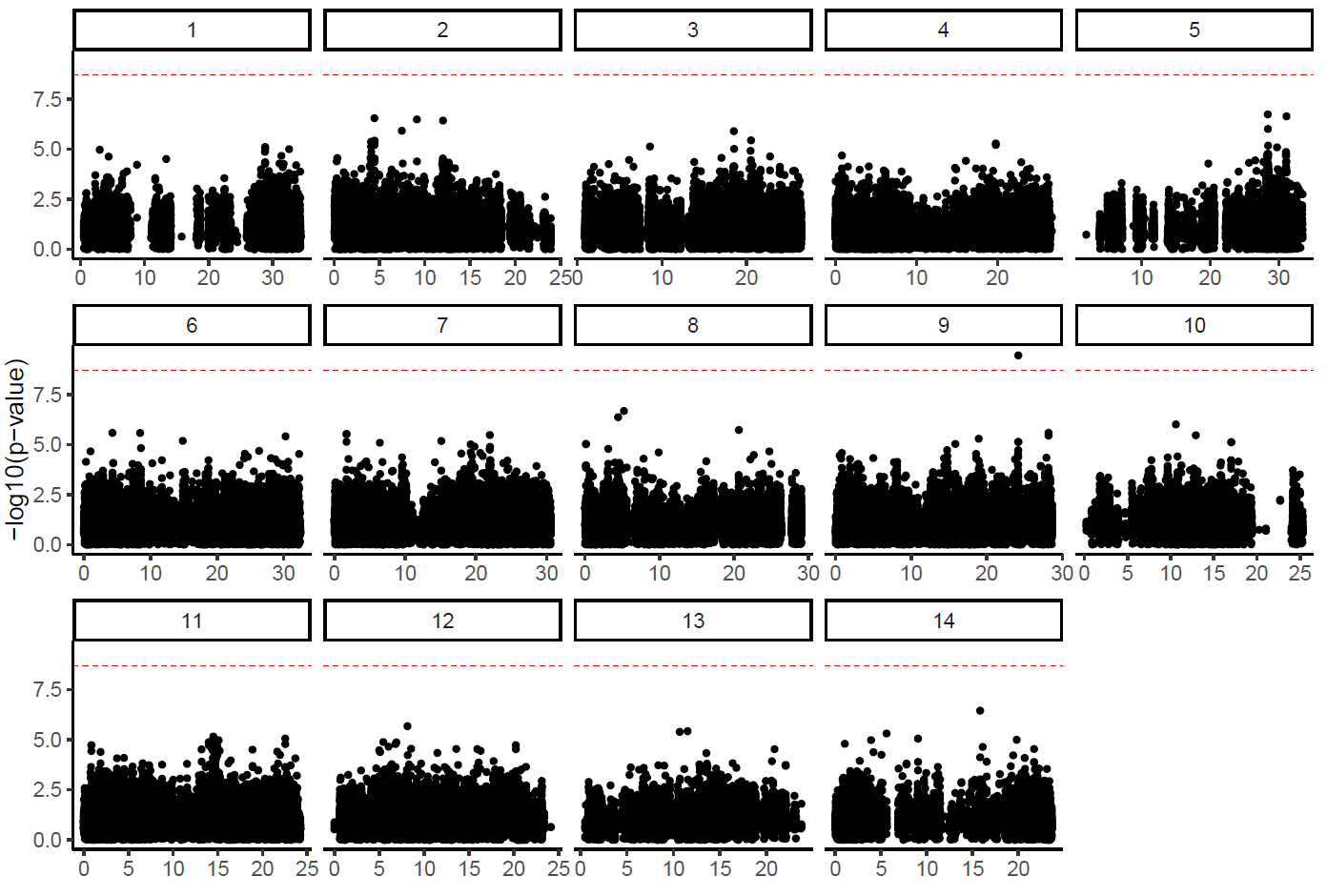

Supplemental Figure 18. Manhattan plot subsetted by chromosomes for bleaching resistance GWAS calculated using deep forereef samples only shows no significant loci. Dotted horizontal red line shows genome-wide cutoffs determined through 10,000 permutations of the GLM, randomly shuffling the trait values, extracting the minimum *p*-value for each run, and then taking the value of the 95^th^ percentile of this distribution (Fuller et al., 2020).

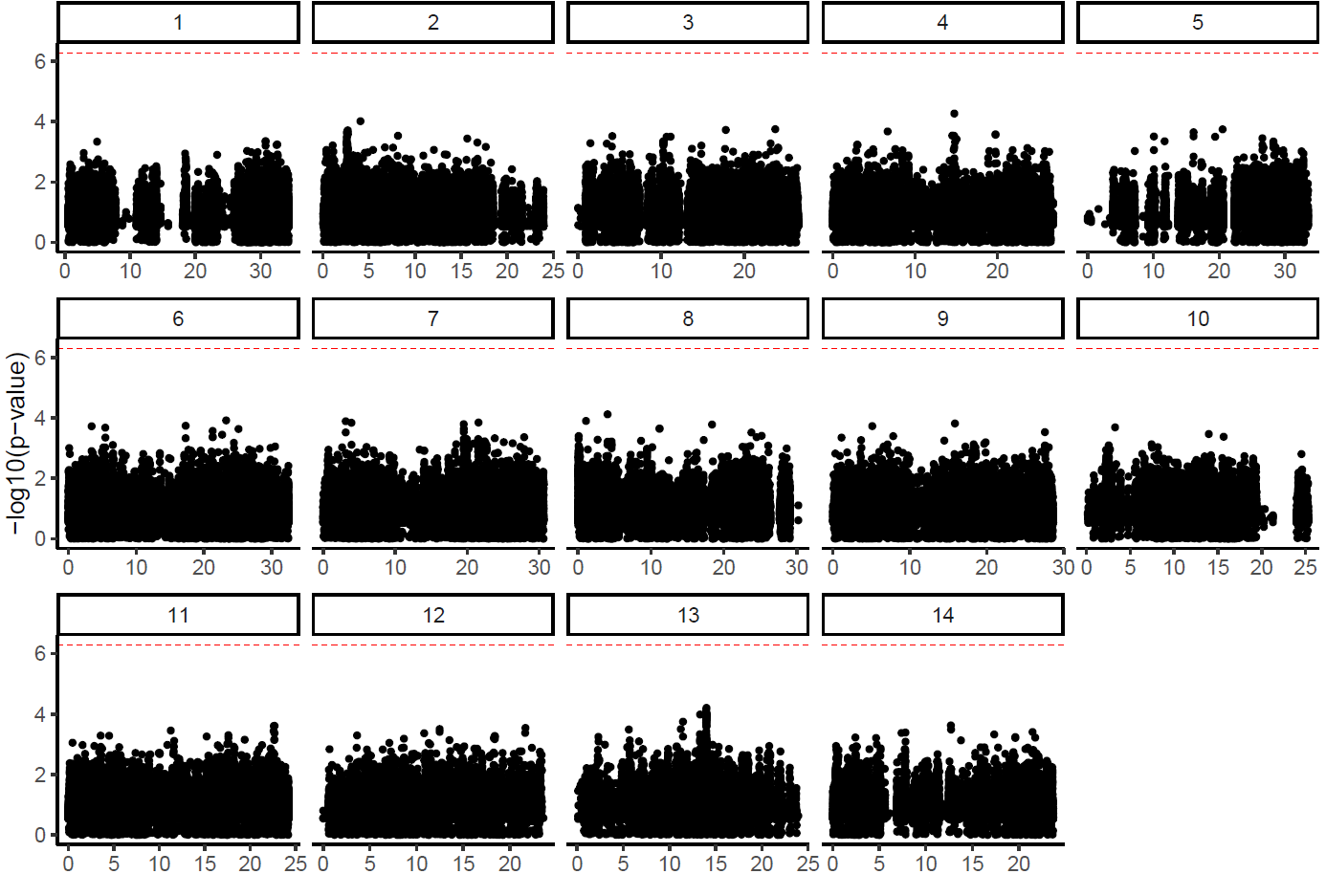

Supplemental Figure 19. Manhattan plot subsetted by chromosomes for bleaching survival GWAS calculated using deep forereef samples only shows no significant loci. Dotted horizontal red line shows genome-wide cutoffs determined through 10,000 permutations of the GLM, randomly shuffling the trait values, extracting the minimum *p*-value for each run, and then taking the value of the 95^th^ percentile of this distribution (Fuller et al., 2020).


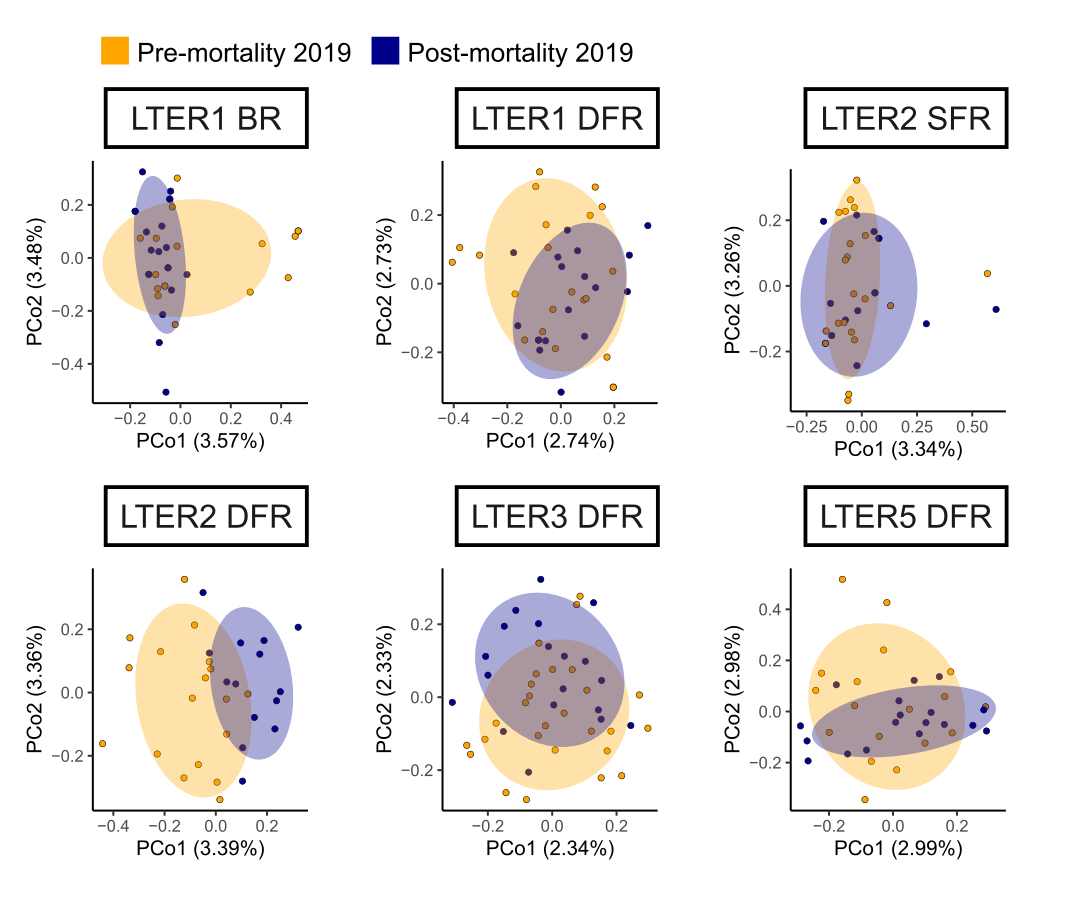


Supplemental Figure 20. Principal Coordinates Analysis (PCoA) plot using a 1 - correlation transformation on the genetic covariance matrix subset for site showing a pattern of post-mortality samples occupying a subset of the genetic variance covered by pre-mortality individuals. Shaded ellipses indicate the 75% confidence region around samples, grouped by pre-/post-mortality. BR: Backreef; SFR: Shallow forereef; DFR: Deep forereef.


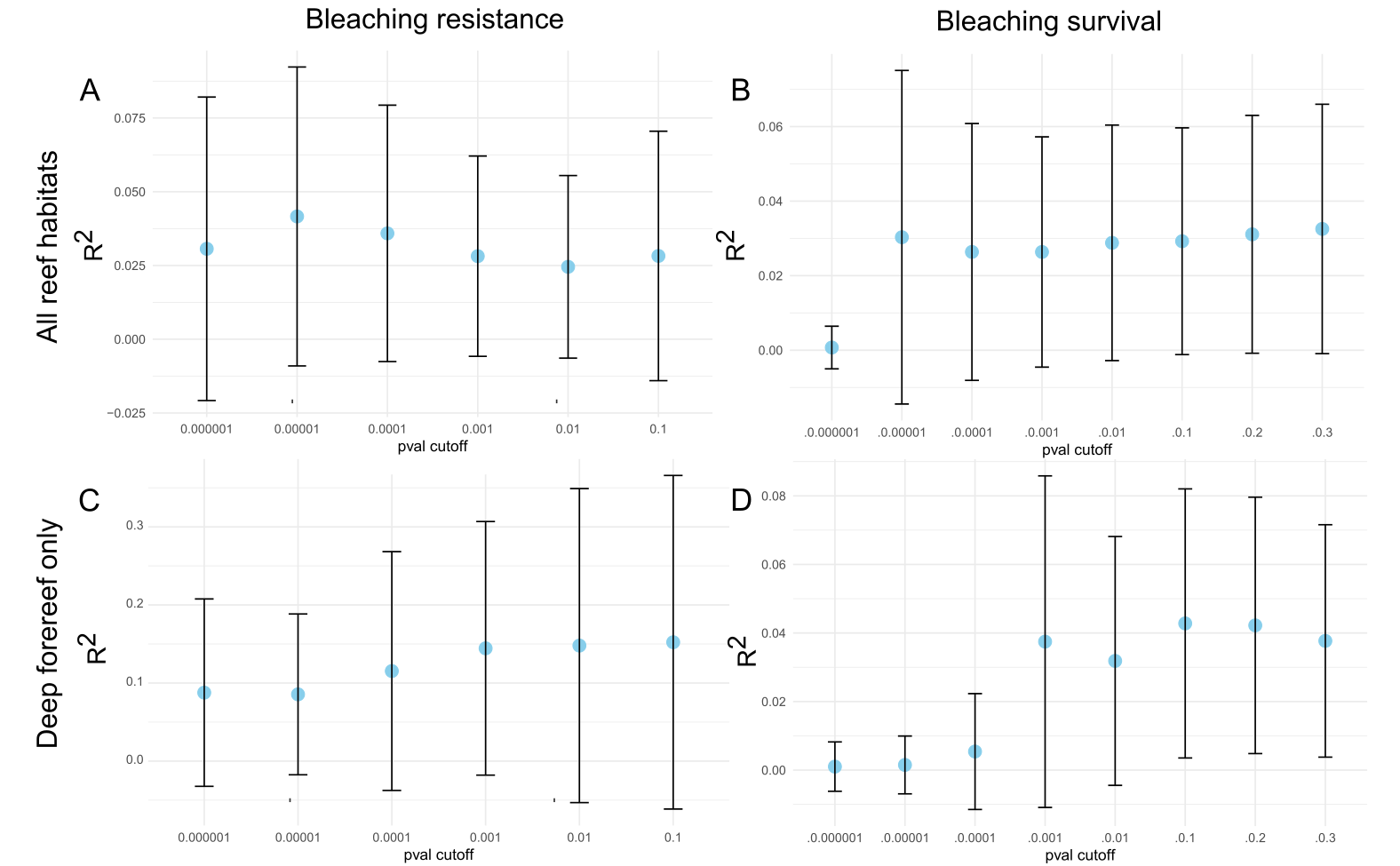


Supplemental Figure 21. Comparison of R^2^ for the 100 partitioned test sets at different GWAS *p*-value thresholds for PGS building. PGS built using backreef, shallow forereef and deep forereef habitats for A) pre-mortality samples taken during the bleaching event with health score as the trait and B) pre- and post-mortality samples with survival as the trait. PGS built using deep forereef habitat only for C) pre-mortality samples taken during the bleaching event with health score as the trait and D) pre- and post-mortality samples with survival as the trait. Plot shows mean and standard deviation of R^2^.


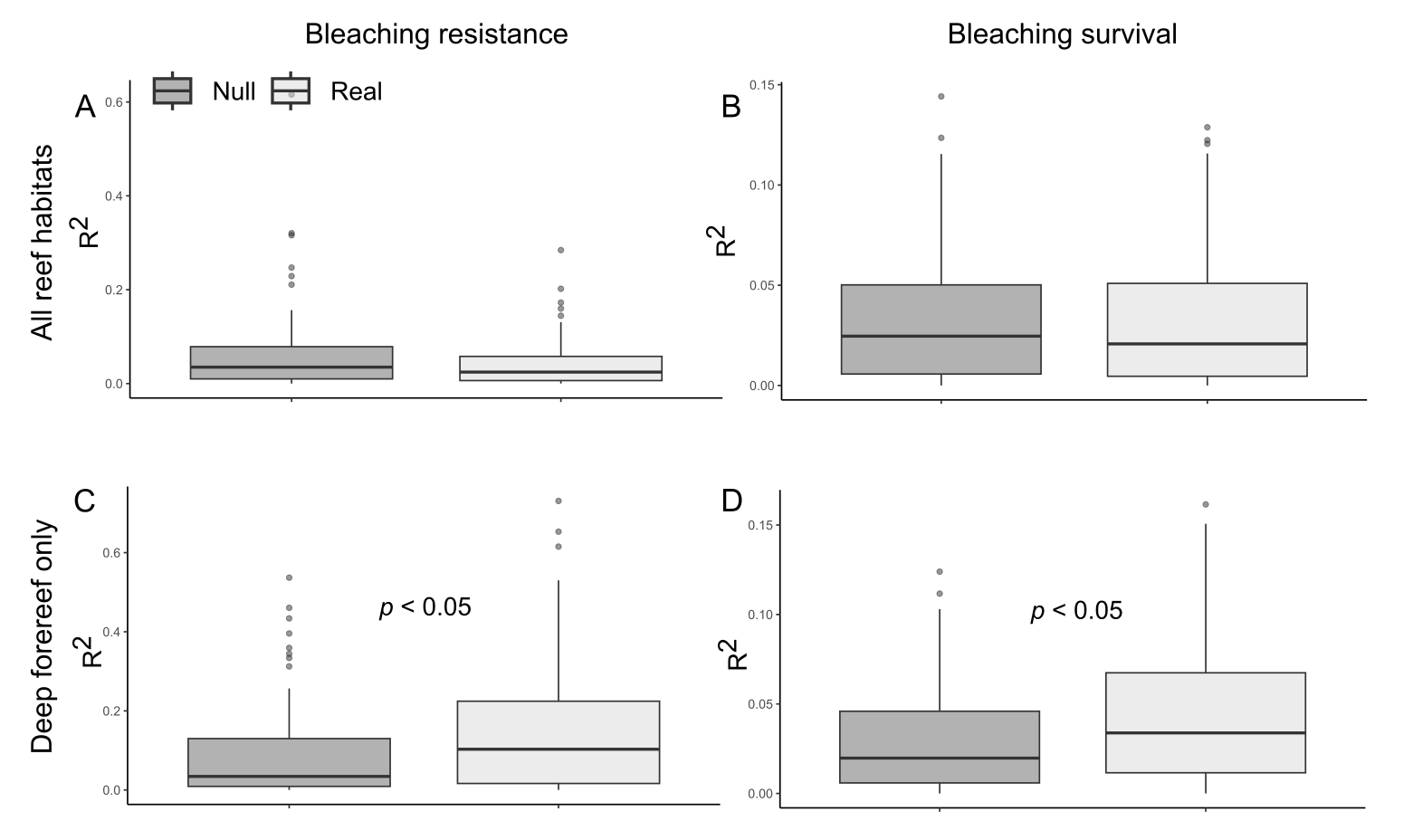


Supplemental Figure 22. Comparison of R^2^ for the 100 partitioned test sets between the real PGS (using optimal *p*-value threshold from Supp. Fig. 15) and a null PGS (randomly selected loci). PGS built using backreef, shallow forereef and deep forereef habitats for A) pre-mortality samples taken during the bleaching event with health score as the trait and B) pre- and post-mortality samples with survival as the trait. PGS built using deep forereef habitat only for C) pre-mortality samples taken during the bleaching event with health score as the trait and D) pre- and post-mortality samples with survival as the trait. Plot shows mean and standard deviation of R^2^.


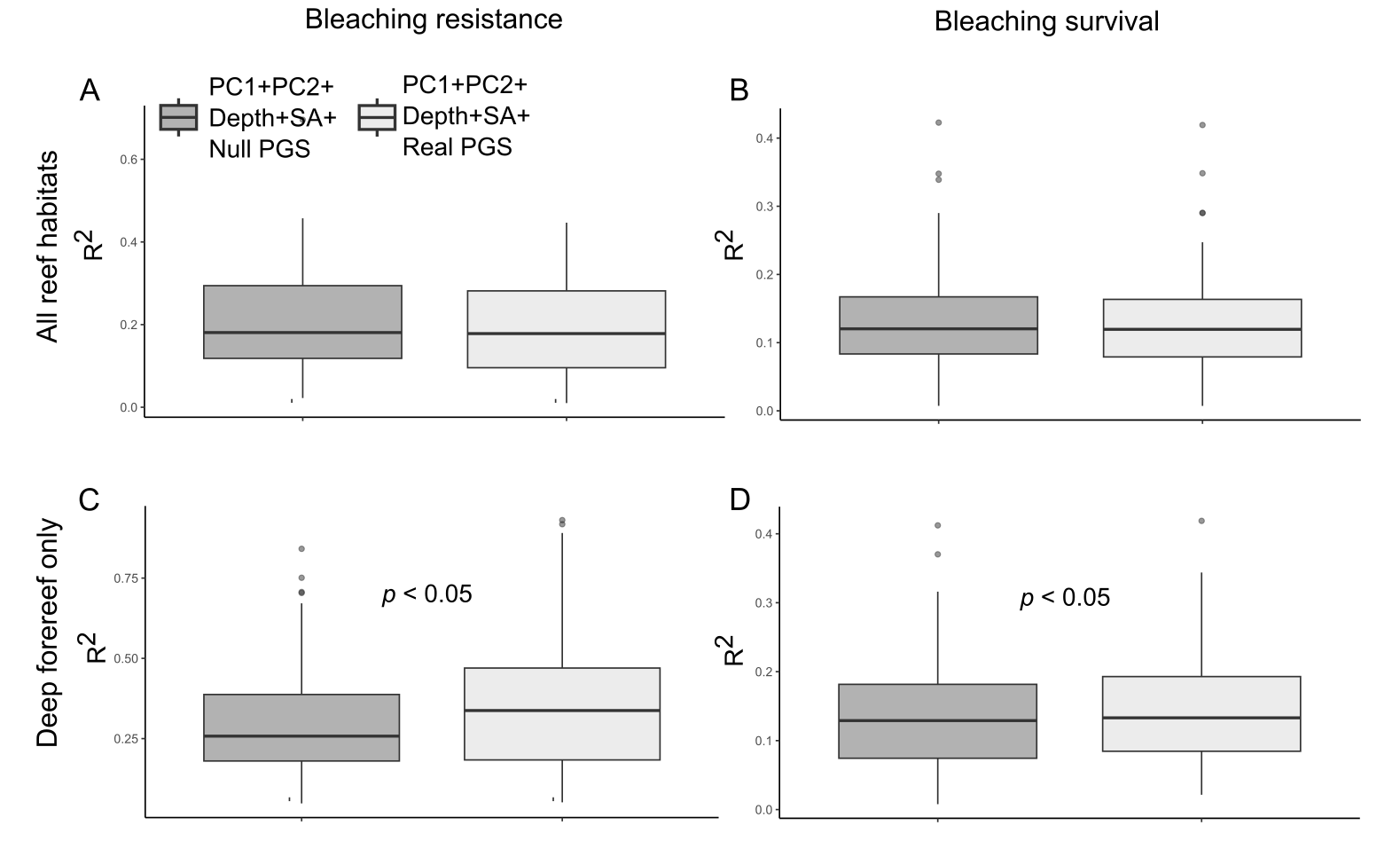


Supplemental Figure 23. Comparison of R^2^ for the 100 partitioned test sets between a model with all covariates except symbiont proportions and the real PGS (using optimal *p*-value threshold from Supp. Fig. 15) versus a model with all covariates except symbiont proportions and a null PGS (randomly selected loci). PGS built using backreef, shallow forereef and deep forereef habitats for A) pre-mortality samples taken during the bleaching event with health score as the trait and B) pre- and post-mortality samples with survival as the trait. PGS built using deep forereef habitat only for C) pre-mortality samples taken during the bleaching event with health score as the trait and D) pre- and post-mortality samples with survival as the trait. Plot shows mean and standard deviation of R^2^. SA: Surface area.


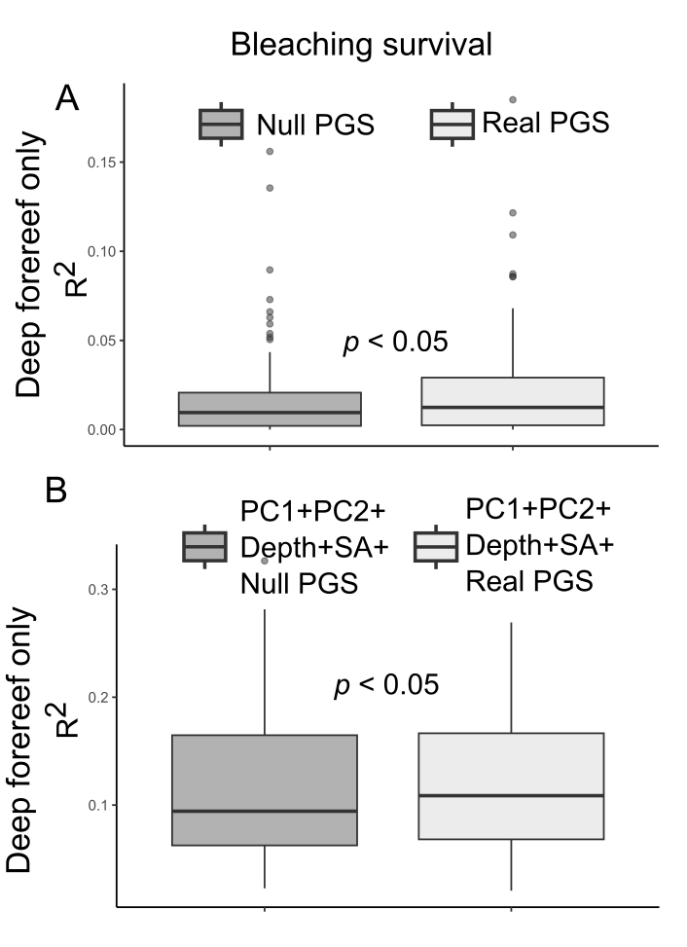


Supplemental Figure 24. No missing data subset. Comparison of R^2^ for the 100 partitioned test sets between A) the real PGS (using optimal *p*-value threshold from Supp. Fig. 15) and a null PGS (randomly selected loci) B) a model with all covariates except symbiont proportions and the real PGS (using optimal *p*-value threshold from Supp. Fig. 15) versus a model with all covariates except symbiont proportions and a null PGS (randomly selected loci). PGS built using deep forereef habitat only for pre- and post-mortality samples with survival as the trait using a subset of loci and samples containing no missing data (Supplemental File 1). Plot shows mean and standard deviation of R^2^.


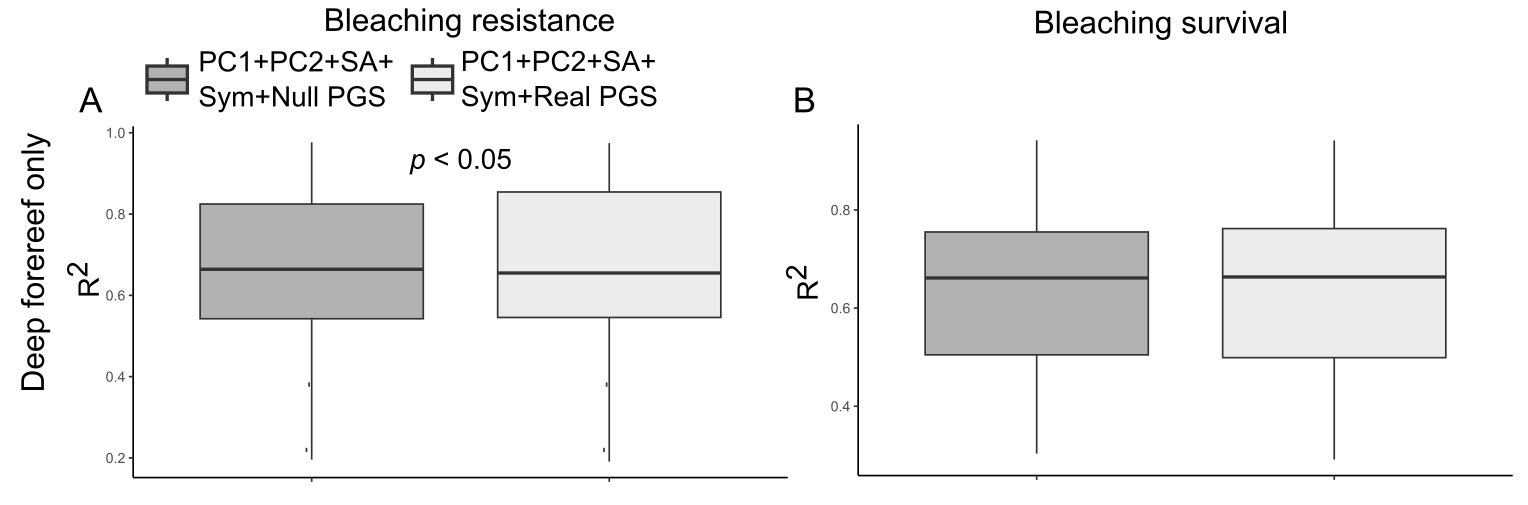


Supplemental Figure 25. Comparison of R^2^ for the 100 partitioned test sets between a model with all covariates (including symbiont proportions) and the real PGS (using optimal *p*-value threshold from Supp. Fig. 15) versus a model with all covariates (including symbiont proportions) and a null PGS (randomly selected loci). PGS built using deep forereef habitat only for A) pre-mortality samples taken during the bleaching event with health score as the trait and B) pre- and post-mortality samples with survival as the trait. Plot shows mean and standard deviation of R^2^.


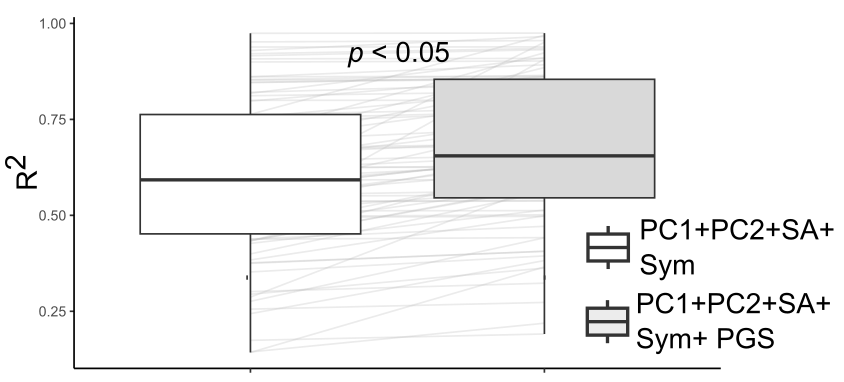


Supplemental Figure 26. Comparison of R^2^ for the 100 partitioned test sets between a model with all covariates (including symbiont proportion) versus a model with all covariates and the real PGS (using optimal *p*-value threshold from Supp. Fig. 15). PGS was built using deep forereef habitat only for pre-mortality samples taken during the bleaching event with health score as the trait. SA: Surface area.


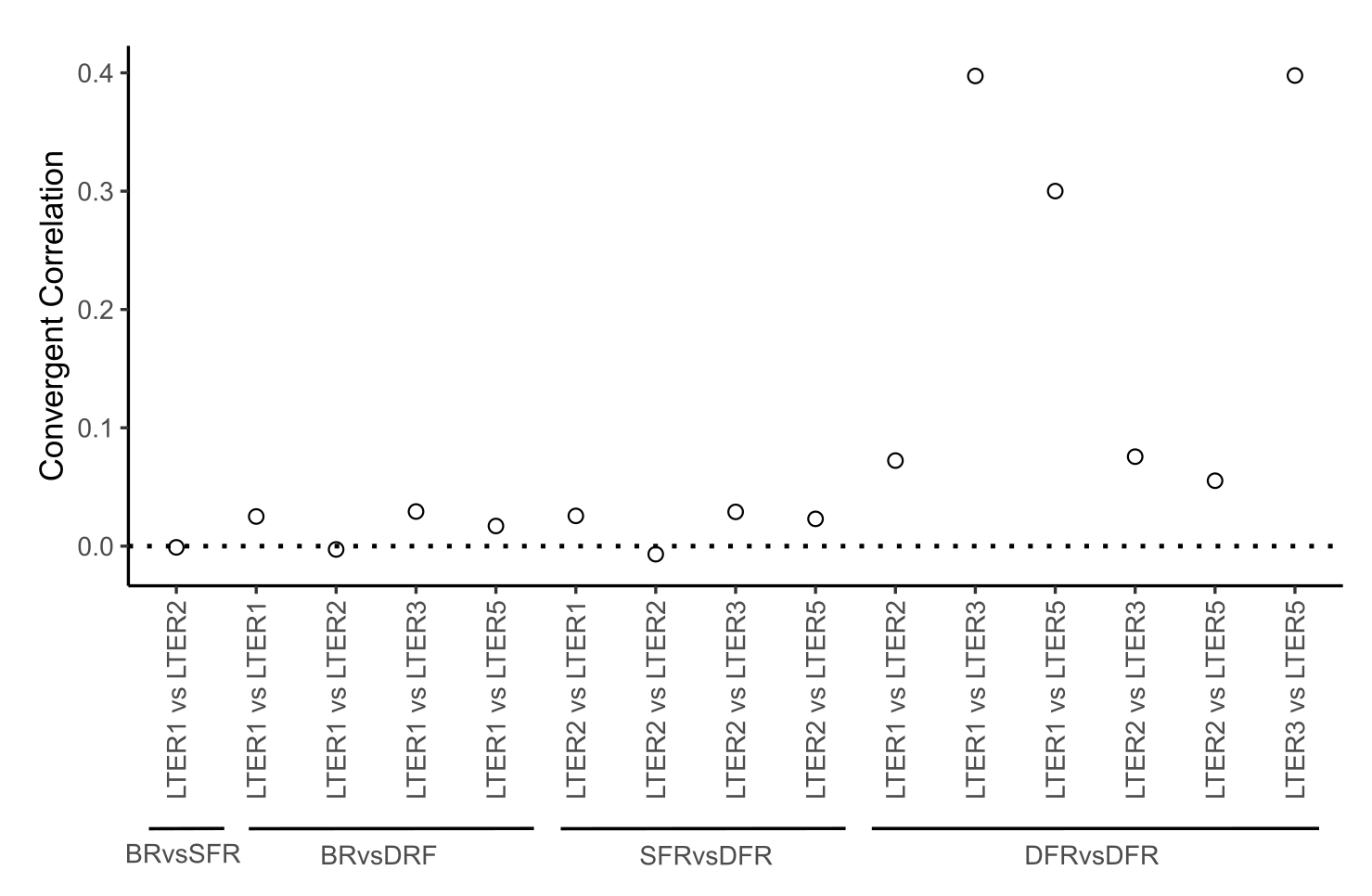


Supplemental Figure 27. Convergent correlation statistic for allele frequency shifts between pre- and post- mortality timepoints using only the deep forereef bleaching survival PGS (Fig. 3C). BR: Backreef; SFR: Shallow forereef; DFR: Deep forereef.


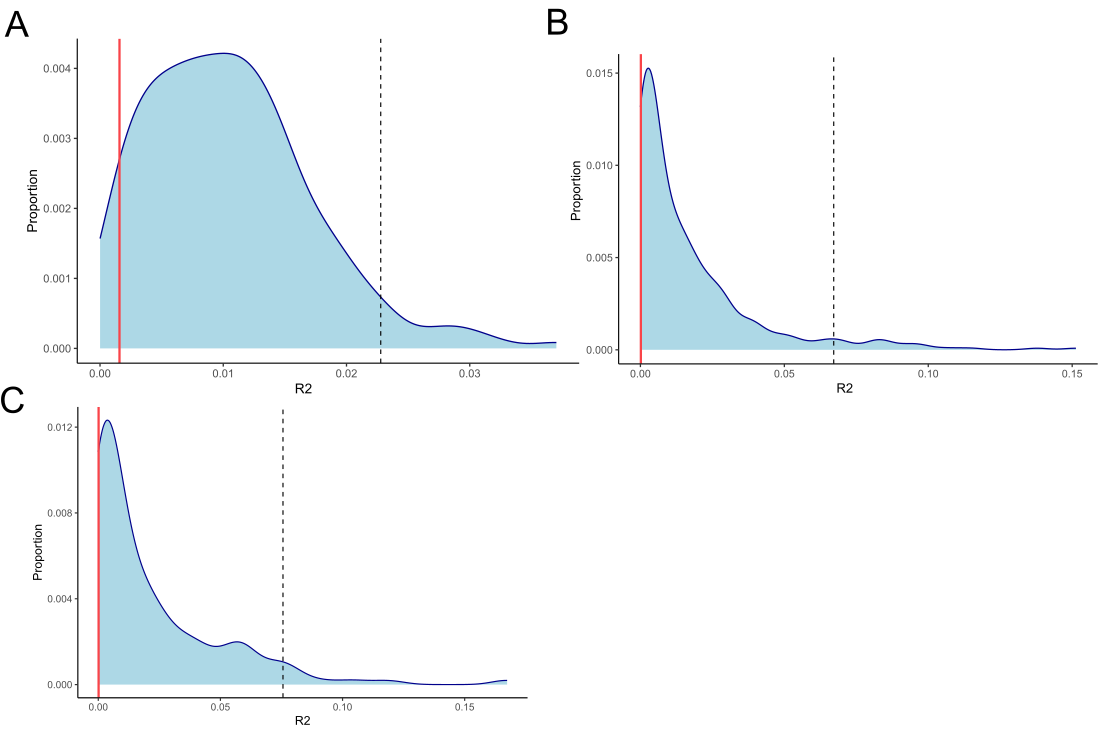


Supplemental Figure 28. Predictive power (R^2^) for backreef bleaching survival PGS for the 500 sets of randomly selected loci (blue distribution) and for the loci passing *p*-value A) < 0.01 B) < 0.001 and C) < 0.0001 thresholds for the deep forereef bleaching survival GWAS. Dotted line shows 95^th^ percentile for the null distribution and red line shows R^2^ for the real PGS.


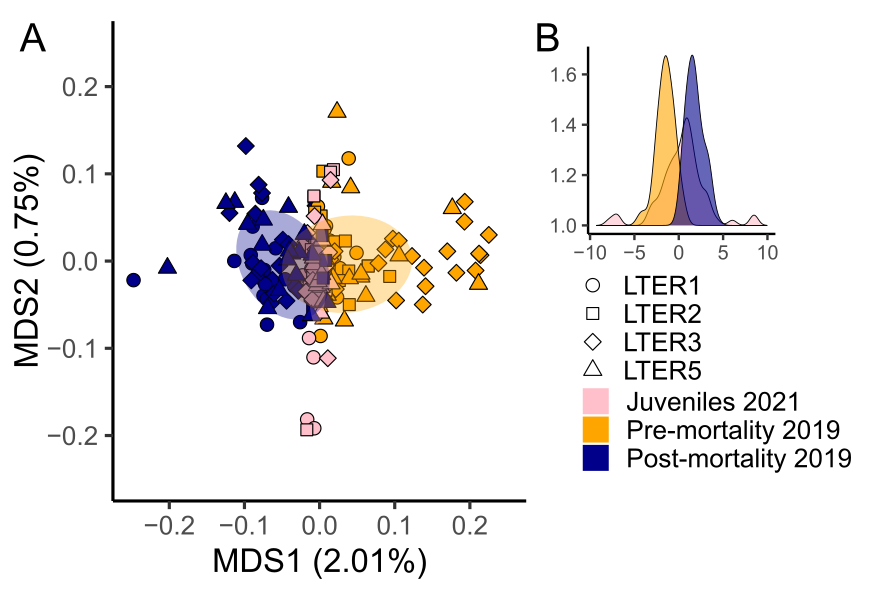


Supplemental Figure 29. MDS (A) and DAPC (B) plots based on genetic covariance matrices from the bleaching survival GWAS outlier loci only, demonstrating the intermediate state of juvenile genotypes.


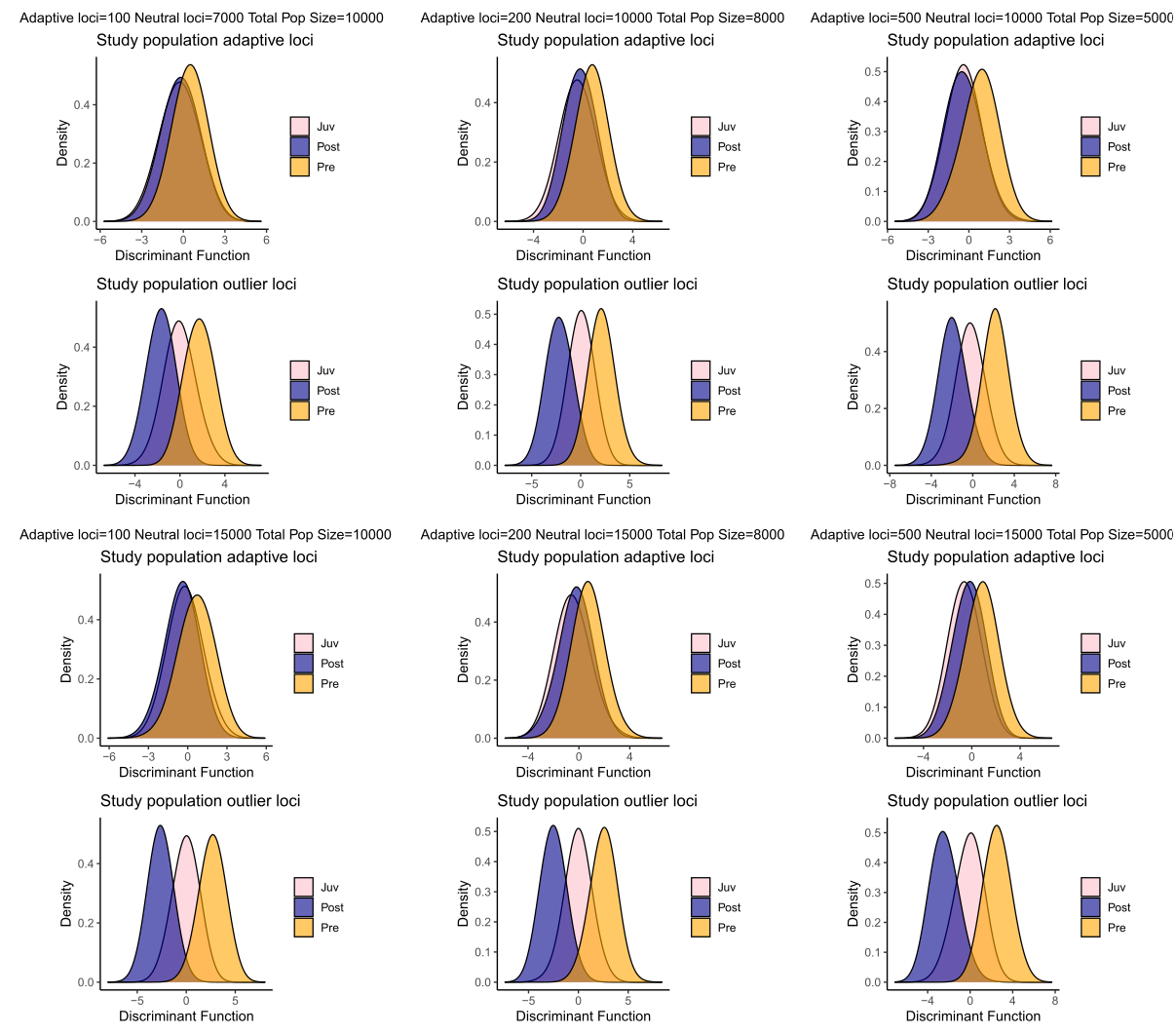

Supplemental Figure 30. Intermediate distribution from juveniles as a function of sampling bias. DAPCs for simulated data across different iterations of adaptive loci (*i.e.*, adaptive loci in the entire population), neutral loci (*i.e.*, neutral loci in the entire population) and population size (*i.e.*, population size of the entire population) examining both adaptive loci (top; true adaptive loci within outlier loci identified by GWAS) and outlier loci (bottom; all loci identified by GWAS).


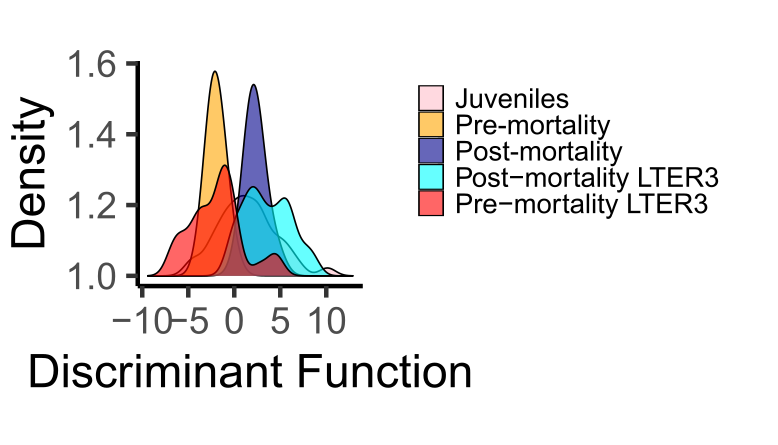
Supplemental Figure 31. DAPC plots based on genetic covariance matrices from the deep forereef bleaching survival GWAS outlier loci plotting LTER3 pre- and post-mortality separately.


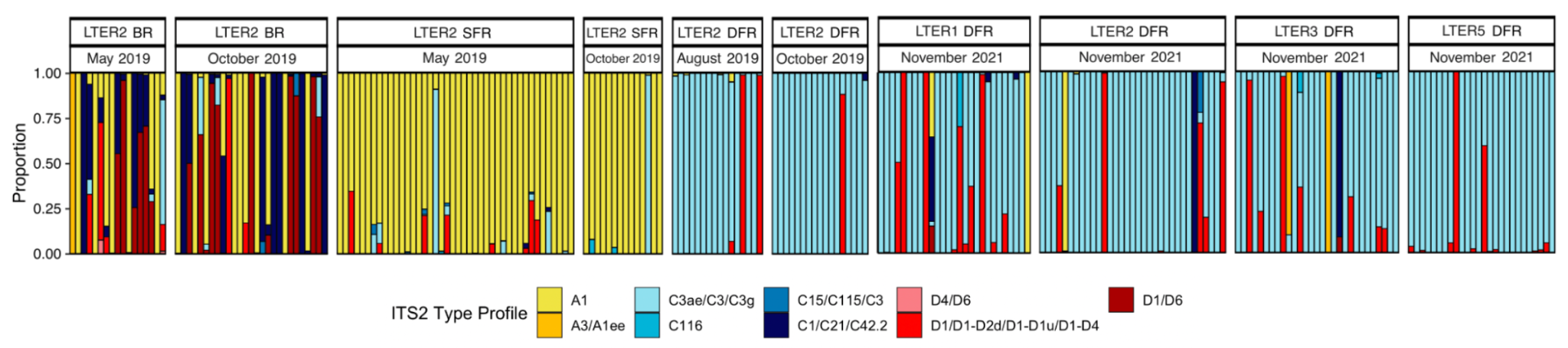

Supplemental Figure 32. Proportion of collapsed ITS2 type profiles from ITS2 metabarcoding data analyzed using SymPortal. Samples include those collected in 2019 at the backreef, shallow forereef, and deep forereef at LTER 2, published in Leinbach et al. (2023), and juveniles collected in 2021 from deep forereef sites (LTER 1, LTER 2, LTER 3, LTER 5). Colored bars correspond to relative abundances of dominant profile types in an individual colony.


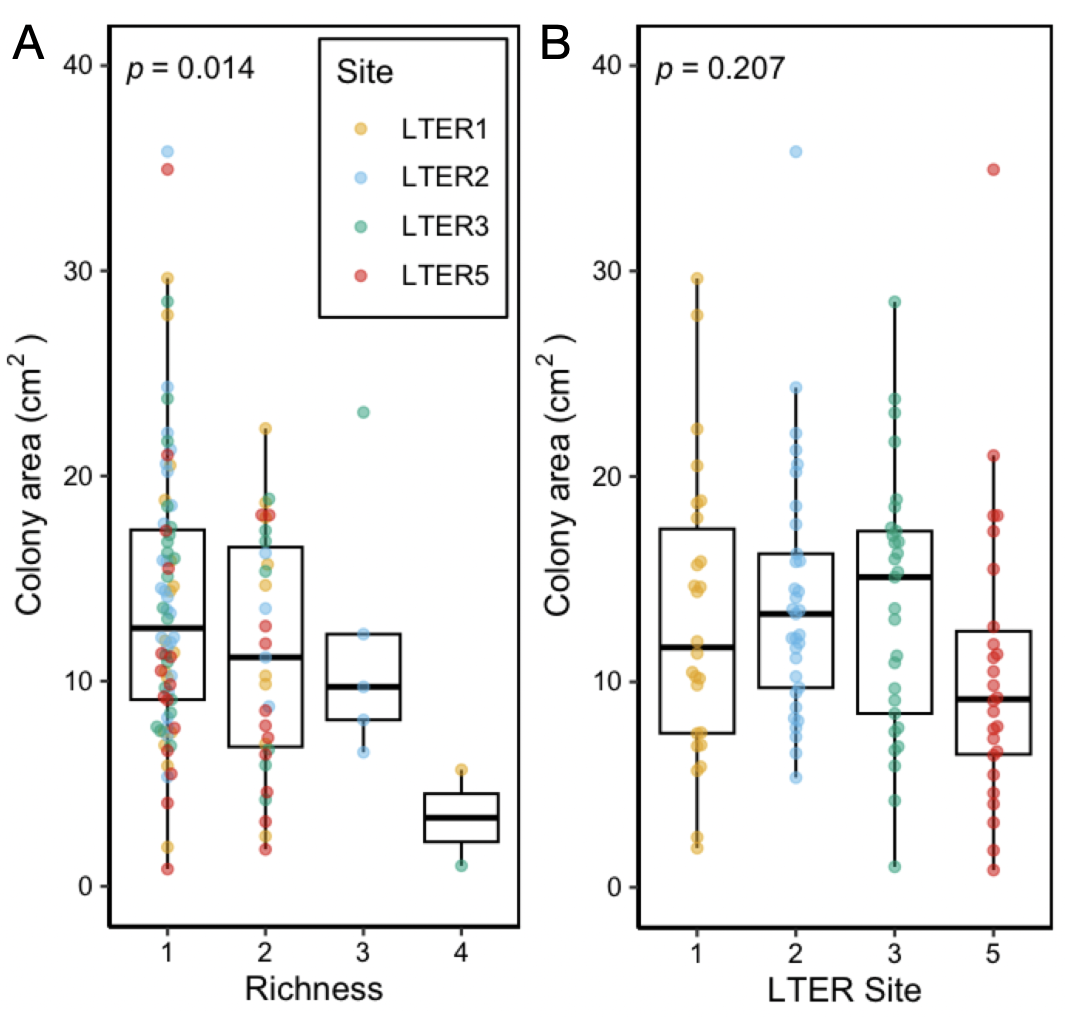


Supplemental Figure 33. Comparisons of juvenile colony area by richness and site (2021 juvenile samples at deep forereef sites only, N = 114). A) Colony area across ITS2 type profile richness. B) Colony area compared between sites. Each dot represents an individual colony.


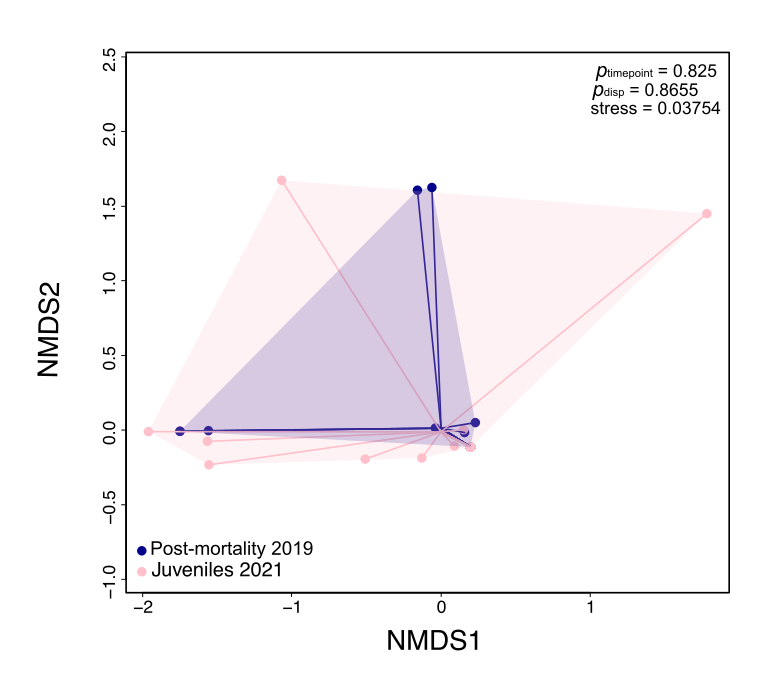
Supplemental Figure 34. Non-metric multidimensional scaling (NMDS) plot of between sample symbiont community structure (LTER 2 deep forereef only) in 2019 and 2021 based on collapsed ITS2 type profiles.


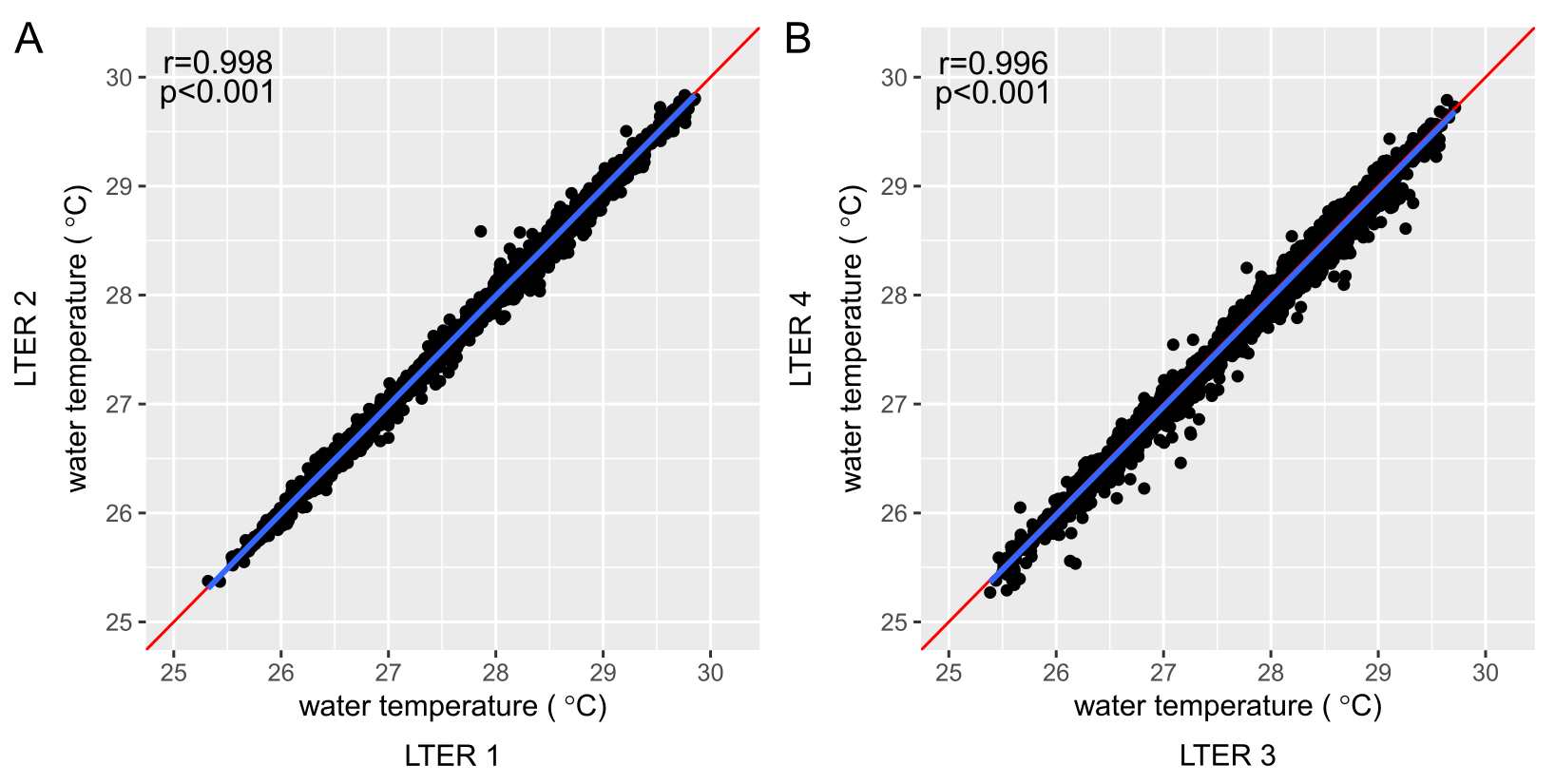


Supplemental Figure 35. Correlation between median daily water temperatures for substituted thermistors for Fig. 1 during periods of available overlapping data from May 2005 to August 2021 for A) North side and B) East side of Mo’orea.


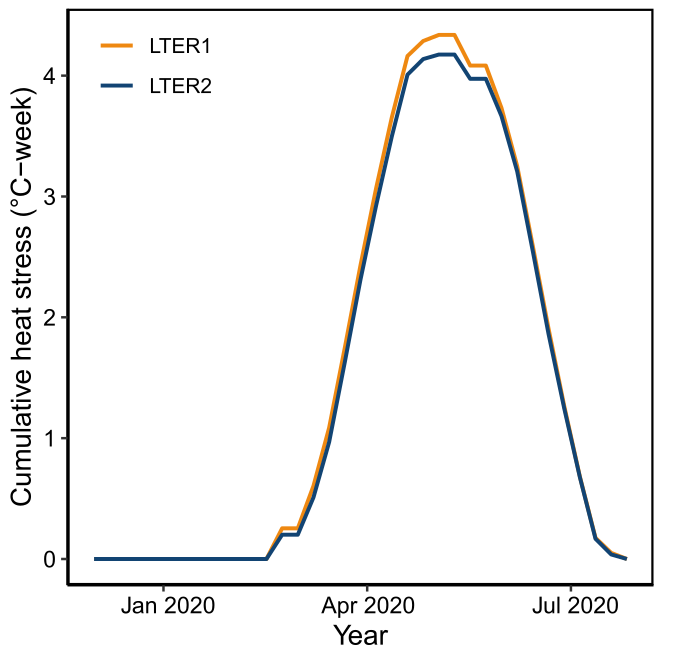


Supplemental Figure 36. Comparison of cumulative heat stress calculated for available overlapping data (*i.e.*, 2020) for substituted thermistors in Fig. 1 during periods of available overlapping data.


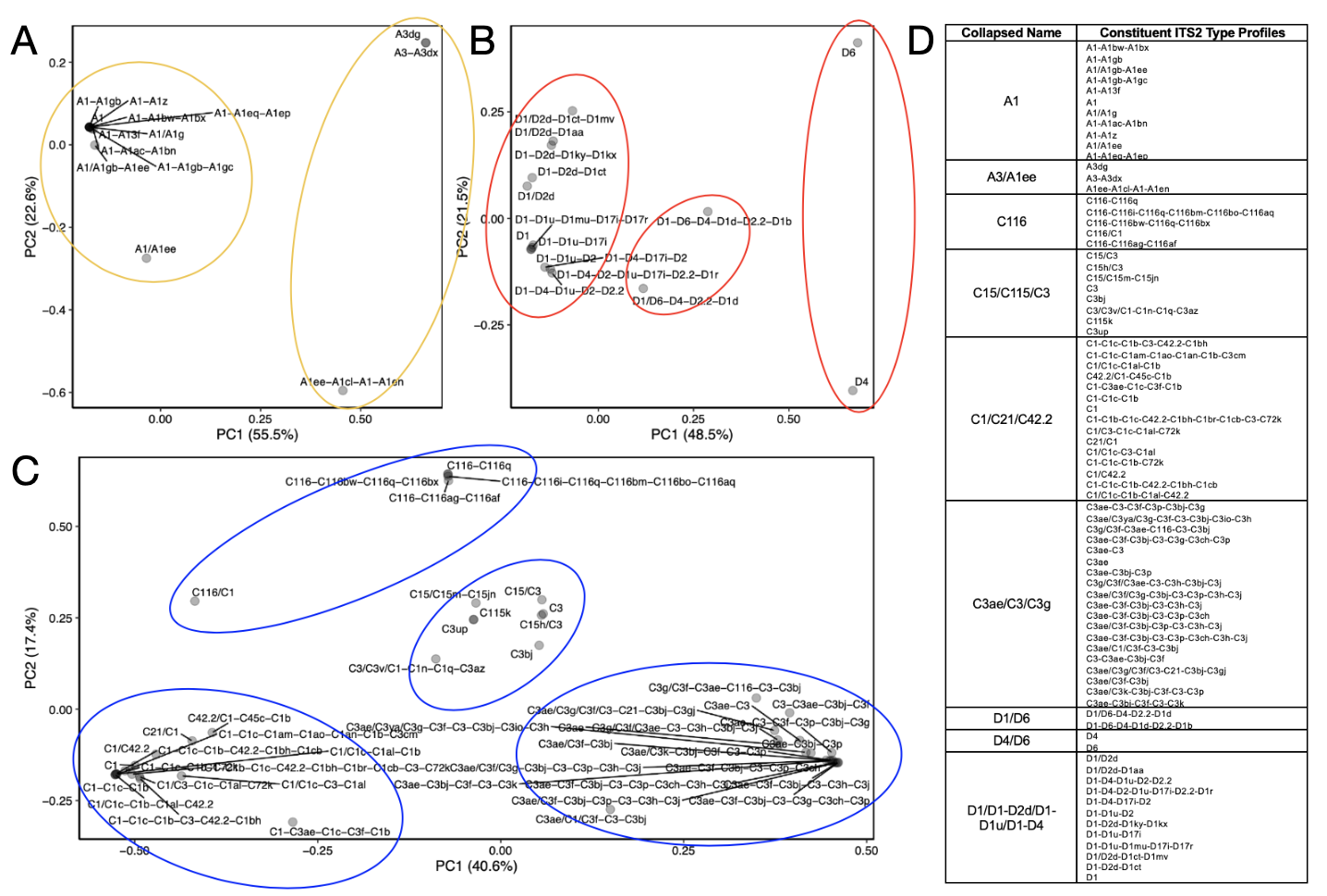


Supplemental Figure 37. Collapsed Symbiodiniaceae ITS2 type profiles of juveniles collected at deep forereef sites in 2021. PCA was conducted based on Bray-Curtis distances provided by SymPortal between A) *Symbiodinium*, B) *Durusdinium*, and C) *Cladocopium* profiles. Elliptical shapes were drawn around profiles based on similarity and/or their distribution along PC1. D) Table of nine collapsed ITS2 type profiles and their constituent ITS2 type profiles (78 profiles total). Collapsed profile names are derived from the dominant defining intragenomic variant (DIV) found within each constituent ITS2 type profile, with slashes separating DIVs by genotype.


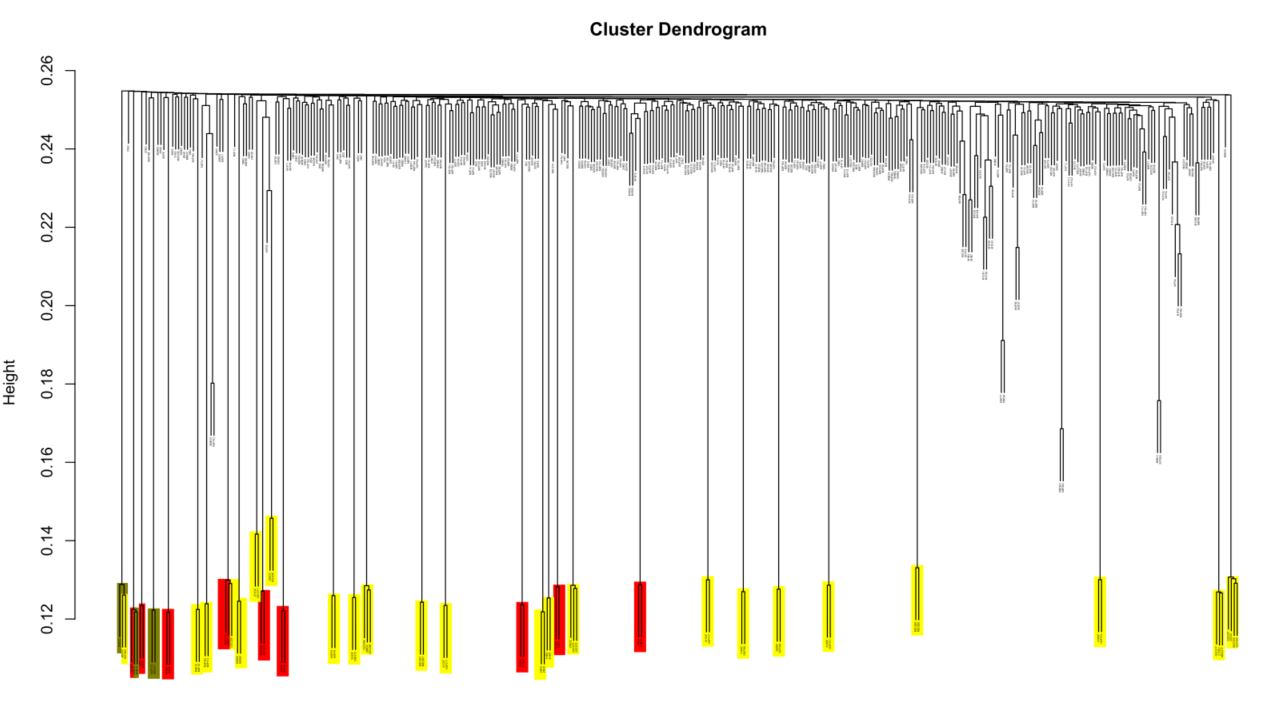


Supplemental Figure 38. Identity by decent dendrogram for all colonies sampled. A height of 0.15 was used to identify clones within timepoints (green) and the same colony or clones across timepoints (red) with technical replicates (yellow).


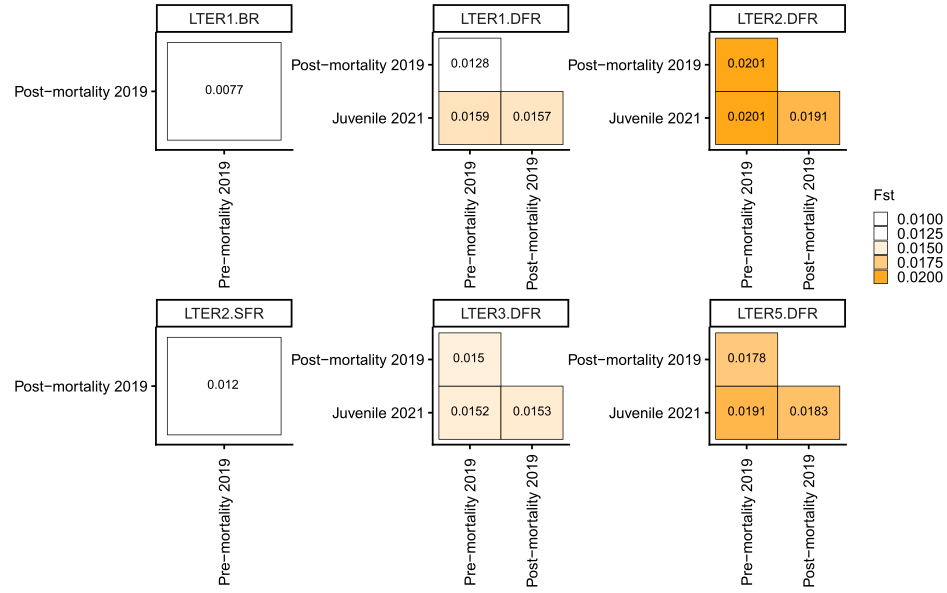


Supplemental Figure 39. Temporal comparisons of pairwise *F*_ST_ within all sites, showing some population structure. BR: Backreef; SFR: Shallow forereef; DFR: Deep forereef.

| **LTER** | **Reef Habitat** | **Collection Timepoint** | **Lat** | **Long** | **Temp data source** | **Bleaching info (N)** | **July 2019 Bleaching & Mortality info (N)** | **WGS data (N) (clones removed)** | **WGS data (N) (half or full sibs)** | **ITS2 (N)** | **Depth (m)** |
| --- | --- | --- | --- | --- | --- | --- | --- | --- | --- | --- | --- |
| LTER 1 | Deep Forereef | May 2019 | -17.475929 | -149.8398389 | LTER 0 | 50 | 23 | 21 | 0 | 0 | 10 |
| LTER 1 | Deep Forereef | October 2019 | -17.475929 | -149.8398389 | LTER 0 | *NA* | *NA* | 21 | 0 | 0 | 10 |
| LTER 1 | Deep Forereef | November 2021 | -17.475929 | -149.8398389 | LTER 0 | *NA* | *NA* | 21 | 4 | 27 | 10 |
| LTER 1 | Shallow Forereef | May 2019 | -17.47628717 | -149.8397745 | LTER 0 | 52 | *NA* | 16 | 4 | 0 | 3-5 |
| LTER 1 | Backreef | May 2019 | -17.4798382 | -149.8400535 | None | 25 | *NA* | 19 | 2 | 0 | 1-3 |
| LTER 1 | Backreef | October 2019 | -17.4798382 | -149.8400535 | None | *NA* | *NA* | 19 | 0 | 0 | 1-3 |
| LTER 2 | Deep Forereef | August 2019 | -17.473111 | -149.817639 | LTER 0 | 66 | *NA* | 0 | 0 | 16 | 10 |
| LTER 2 | Deep Forereef | May 2019 | -17.473111 | -149.817639 | LTER 0 | 65 | 20 | 18 | 0 | 0 | 10 |
| LTER 2 | Deep Forereef | October 2019 | -17.473111 | -149.817639 | LTER 0 | *NA* | *NA* | 14 | 0 | 17 | 14 |
| LTER 2 | Deep Forereef | November 2021 | -17.473111 | -149.817639 | LTER 0 | *NA* | *NA* | 19 | 0 | 33 | 10 |
| LTER 2 | Shallow Forereef | May 2019 | -17.4738068795 | -149.8172635 | LTER 0 | 63 | 20 | 23 | 0 | 42 | 3-5 |
| LTER 2 | Shallow Forereef | October 2019 | -17.4738068795 | -149.8172635 | LTER 0 | *NA* | *NA* | 13 | 0 | 14 | 3-5 |
| LTER 2 | Backreef | May 2019 | -17.476204 | -149.813934 | None | 25 | *NA* | 0 | 0 | 17 | 1-3 |
| LTER 2 | Backreef | October 2019 | -17.476204 | -149.813934 | None | *NA* | *NA* | 0 | 0 | 27 | 1-3 |
| LTER 3 | Deep Forereef | May 2019 | -17.51547255 | -149.7620307 | LTER 4 | 72 | 45 | 28 | 2 | 0 | 10 |
| LTER 3 | Deep Forereef | October 2019 | -17.51547255 | -149.7620307 | LTER 4 | *NA* | *NA* | 19 | 0 | 0 | 10 |
| LTER 3 | Deep Forereef | November 2021 | -17.51547255 | -149.7620307 | LTER 4 | *NA* | *NA* | 21 | 0 | 29 | 10 |
| LTER 3 | Shallow Forereef | May 2019 | -17.513447 | -149.762074 | LTER 4 | 27 | *NA* | 14 | 0 | 0 | 3-5 |
| LTER 5 | Deep Forereef | May 2019 | -17.57837964 | -149.8758341 | LTER 5 | 100 | 75 | 14 | 2 | 0 | 10 |
| LTER 5 | Deep Forereef | October 2019 | -17.57837964 | -149.8758341 | LTER 5 | *NA* | *NA* | 19 | 0 | 0 | 10 |
| LTER 5 | Deep Forereef | November 2021 | -17.57837964 | -149.8758341 | LTER 5 | *NA* | *NA* | 16 | 0 | 26 | 10 |
| LTER 5 | Backreef | May 2019 | 17.57552605 | -149.876156 | LTER 5 | 19 | *NA* | 14 | 0 | 0 | 1-3 |
| TOTAL | *NA* | *NA* | *NA* | *NA* | *NA* | 564 | 183 | 349 | 14 | 248 | *NA* |

Supplemental Table 1. Sample and site information for bleaching surveys, temperature source data, July bleaching and mortality surveys, WGS and ITS2 sequencing. For surveys N corresponds to the number of colonies surveyed, not the number of bleaching or dead corals.

| **Explanatory variable** | **Conditional variables** | **R^2^** | **p-value** |
| --- | --- | --- | --- |
| Dominated by *Symbiodinium* (Yes/No) | Depth + LTER1 + LTER2 + LTER3 + LTER5 | <0.01 | 0.614 |
| Dominated by *Cladocopium* (Yes/No) | Depth + LTER1 + LTER2 + LTER3 + LTER5 | <0.01 | 0.648 |
| Dominated by *Durusdinium* (Yes/No) | Depth + LTER1 + LTER2 + LTER3 + LTER5 | <0.01 | 0.434 |

| **Explanatory variable** | **Conditional variables** | **R^2^** | **p-value** |
| --- | --- | --- | --- |
| Host PCos | Depth + LTER1 + LTER2 + LTER3 + LTER5 | <0.01 | 0.754 |
| Host PCos | None | <0.01 | 0.783 |
| Depth | Host PCos + LTER1 + LTER2 + LTER3 + LTER5 | 0.14 | <0.001 |
| Depth | None | 0.14 | <0.001 |

Supplemental Table 2. RDA model using a combination of explanatory and conditional variables with the response variable as host genetic variation. R^2^ is adjR^2^.

| **Explanatory variable** | **Conditional variables** | **R^2^** | **p-value** |
| --- | --- | --- | --- |
| Dominated by *Symbiodinium* (Yes/No) | Depth + LTER1 + LTER2 + LTER3 + LTER5 | <0.01 | 0.614 |
| Dominated by *Cladocopium* (Yes/No) | Depth + LTER1 + LTER2 + LTER3 + LTER5 | <0.01 | 0.648 |
| Dominated by *Durusdinium* (Yes/No) | Depth + LTER1 + LTER2 + LTER3 + LTER5 | <0.01 | 0.434 |

Supplemental Table 3. RDA model using a combination of explanatory and conditional variables with the response variable as symbiont genera proportions. R^2^ is adjR^2^.

References

Adams, N. E., Bandivadekar, R. R., Battey, C. J., Clark, M. W., Epperly, K., Ruegg, K., Tell, L. A., & Bay, R. A. (2023). Widespread gene flow following range expansion in Anna’s Hummingbird. *Molecular Ecology*, *32*(12), 3089–3101. https://doi.org/10.1111/mec.16928

Anders, S., Huber, W., Nagalakshmi, U., Wang, Z., Waern, K., Shou, C., Raha, D., Gerstein, M., Snyder, M., Mortazavi, A., Williams, B., McCue, K., Schaeffer, L., Wold, B., Robertson, G., Hirst, M., Bainbridge, M., Bilenky, M., Zhao, Y., … Salzberg, S. (2010). Differential expression analysis for sequence count data. *Genome Biology*, *11*(10), R106. https://doi.org/10.1186/gb-2010-11-10-r106

Edmunds, P., & Moorea Coral Reef LTER. (2022). MCR LTER: Coral Reef: Long-term Population and Community Dynamics: Corals, ongoing since 2005. In *LTER Network Member Node.* https://pasta.lternet.edu/package/metadata/eml/knb-lter-mcr/4/39

Evanno, G., Regnaut, S., & Goudet, J. (2005). Detecting the number of clusters of individuals using the software STRUCTURE: A simulation study. *Molecular Ecology*, *14*(8), 2611–2620. https://doi.org/10.1111/j.1365-294X.2005.02553.x

Fifer, J. E., Yasuda, N., Yamakita, T., Bove, C. B., & Davies, S. W. (2022). Genetic divergence and range expansion in a western North Pacific coral. *Science of The Total Environment*, *813*, 152423. https://doi.org/10.1016/j.scitotenv.2021.152423

Fox, E. A., Wright, A. E., Fumagalli, M., & Vieira, G. (2019). ngsLD: evaluating linkage disequilibrium using genotype likelihoods. *Bioinformatics*, 1–22.

Fuller, Z. L., Mocellin, V. J. L., Morris, L. A., Cantin, N., Shepherd, J., Sarre, L., Peng, J., Liao, Y., Pickrell, J., Andolfatto, P., Matz, M., Bay, L. K., & Przeworski, M. (2020). Population genetics of the coroal Acropora millepora: Toward genomic prediction of bleaching. *Science*, *369*(6501). https://doi.org/10.1126/science.aba4674

Korneliussen, T. S., Albrechtsen, A., & Nielsen, R. (2014). ANGSD: Analysis of Next Generation Sequencing Data. *BMC Bioinformatics*, *15*(1), 1–13. https://doi.org/10.1186/s12859-014-0356-4

Leichter, J. J., Seydel, K., & Gotschalk C. (2019). MCR LTER: Coral Reef: Benthic Water Temperature, ongoing since 2005 ver 12. In *Environmental Data Initiative*.

Leinbach, S. E., Speare, K. E., & Strader, M. E. (2023). Reef habitats structure symbiotic microalgal assemblages in corals and contribute to differential heat stress responses. *Coral Reefs*, *42*(1), 205–217. https://doi.org/10.1007/s00338-022-02316-w

Liu, X., & Fu, Y. X. (2020). Stairway Plot 2: demographic history inference with folded SNP frequency spectra. *Genome Biology*, *21*(1), 1–9. https://doi.org/10.1186/s13059-020-02196-9

López-Nandam, E. H., Albright, R., Hanson, E. A., Sheets, E. A., & Palumbi, S. R. (2023). Mutations in coral soma and sperm imply lifelong stem cell renewal and cell lineage selection. *Proceedings of the Royal Society B: Biological Sciences*, *290*(1991). https://doi.org/10.1098/rspb.2022.1766

Okansen, J., Blanchet, F. G., Friendly, M., Kindt, R., Legendre, P., McGlinn, D., Minchin, P. R., O’Hara, R. B., Simpson, G. L., & Solymos, P. (2020). *vegan: community ecology package. R package version 2.5–7*.

Pratchett, M. S., McCowan, D., Maynard, J. A., & Heron, S. F. (2013). Changes in Bleaching Susceptibility among Corals Subject to Ocean Warming and Recurrent Bleaching in Moorea, French Polynesia. *PLoS ONE*, *8*(7). https://doi.org/10.1371/journal.pone.0070443

R Core Team. (2023). *R: A language and environment for statistical computing.* R Foundation for Statistical Computing.

Rasmussen, M. S., Garcia-Erill, G., Korneliussen, T. S., Wiuf, C., & Albrechtsen, A. (2022). Estimation of site frequency spectra from low-coverage sequencing data using stochastic EM reduces overfitting, runtime, and memory usage. *Genetics*, *222*(4). https://doi.org/10.1093/genetics/iyac148

Ripley, B., Venables, B., Bates, D. M., Hornik, K., Gebhardt, A., Firth, D., & Ripley, M. B. (2013). Package ‘mass.’ *Cran r*, *538*, 113–120.

Rose, N. H., Bay, R. A., Morikawa, M. K., Thomas, L., Sheets, E. A., & Palumbi, S. R. (2021). Genomic analysis of distinct bleaching tolerances among cryptic coral species. *Proceedings of the Royal Society B: Biological Sciences*, *288*(1960). https://doi.org/10.1098/rspb.2021.0678

Schneider, C. A., Rasband, W. S., & Eliceiri, K. W. (2012). NIH Image to ImageJ: 25 years of image analysis. In *Nature Methods* (Vol. 9, Issue 7, pp. 671–675). https://doi.org/10.1038/nmeth.2089

Siebeck, U. E., Marshall, N. J., Klüter, A., & Hoegh-Guldberg, O. (2006). Monitoring coral bleaching using a colour reference card. *Coral Reefs*, *25*(3), 453–460. https://doi.org/10.1007/s00338-006-0123-8

Skotte, L., Korneliussen, T. S., & Albrechtsen, A. (2013). Estimating individual admixture proportions from next generation sequencing data. *Genetics*, *195*(3), 693–702. https://doi.org/10.1534/genetics.113.154138

Zhou, X., Appl, A., & Author, S. (2017). A UNIFIED FRAMEWORK FOR VARIANCE COMPONENT ESTIMATION WITH SUMMARY STATISTICS IN GENOME-WIDE ASSOCIATION STUDIES 1 HHS Public Access Author manuscript. *Ann Appl Stat*, *11*(4), 2027–2051. https://doi.org/10.1214/17-AOAS1052SUPP;.pdf

Zhou, X., & Stephens, M. (2012). Genome-wide efficient mixed-model analysis for association studies. *Nature Genetics*, *44*(7), 821–824. https://doi.org/10.1038/ng.2310
